# Trends in *Helicobacter pylori* prevalence, force of infection, and attributable disease burden in China: a systematic review and Bayesian modelling study

**DOI:** 10.1016/j.lanwpc.2026.101979

**Published:** 2026-09-10

**Authors:** Dianqin Sun, Weiran Han, Fernando Alarid-Escudero, Jorge A. Roa-Contreras, Shaoming Wang, Duco T. Mülder, Robert J. Huang, Uri Ladabaum, Manon C.W. Spaander, M. Constanza Camargo, Jin Young Park, Wanqing Chen, Iris Lansdorp-Vogelaar, Jan A.C. Hontelez

**Affiliations:** a Department of Public Health, Erasmus MC, University Medical Center Rotterdam, Rotterdam, the Netherlands; b Department of Health Policy, School of Medicine, Stanford University, Stanford, CA, United States; c Center for Health Policy, Freeman Spogli Institute, Stanford University, CA, United States; d National Cancer Center/National Clinical Research Center for Cancer/Cancer Hospital, Chinese Academy of Medical Sciences and Peking Union Medical College, Beijing, China; e Early Detection, Prevention, and Infections Branch, International Agency for Research on Cancer, Lyon, France; f Division of Gastroenterology and Hepatology, Department of Medicine, Stanford University School of Medicine, Stanford, United States; g Department of Gastroenterology and Hepatology, Erasmus MC, University Medical Center Rotterdam, the Netherlands; h Division of Cancer Epidemiology and Genetics, National Cancer Institute, National Institutes of Health, Rockville, MD, United States; i Heidelberg Institute of Global Health (HIGH), Medical Faculty and University Hospital, Heidelberg University, Heidelberg, Germany

**Keywords:** Helicobacter pylori, Stomach cancer, Bayesian catalytic model, Force of infection, China

## Abstract

**Background:**

*Helicobacter pylori* infection is the leading cause of gastric cancer (GC) and peptic ulcer disease (PUD). China has one of the world's largest populations living with *H. pylori* infection, but long-term age-specific infection dynamics and attributable disease burden remain incompletely characterised.

**Methods:**

We systematically reviewed studies reporting *H*. *pylori* prevalence in China and extracted age-specific prevalence data. We developed a Bayesian catalytic model to jointly synthesise current infection, seropositivity, and ever-infection prevalence, estimating age-specific force of infection (FOI) and prevalence from 1990 to 2023 while accounting for study-level heterogeneity and diagnostic misclassification. Modelled prevalence estimates were combined with published relative risks and national case counts to estimate population attributable fractions (PAFs) and numbers of GC and PUD cases attributable to *H*. *pylori*.

**Findings:**

We included 181 studies in the Bayesian model fitting. Cohorts born after 1980 experienced substantially lower childhood FOI than earlier cohorts, indicating reduced early-life acquisition. However, population prevalence declined only modestly, from 43.1% (95% credible interval 39.1–47.4) in 1990 to 36.3% (33.2–39.6) in 2023. In 2023, estimated PAFs were 70.1% (95% uncertainty interval 51.4–82.4) for non-cardia GC and 49.4% (20.5–70.7) for cardia GC. *H*. *pylori* was associated with approximately 340,000–370,000 GC cases and 160,000–210,000 PUD cases, predominantly duodenal ulcers.

**Interpretation:**

*H*. *pylori* prevalence and attributable disease burden remain high in China. Our estimates suggest a more gradual decline than implied by previous studies, supporting consideration of active *H. pylori* control strategies.

**Funding:**

China Scholarship Council, EU4Health.

**Research in context**

**Evidence before this study**

We searched PubMed and Embase from January 2020 to April 2025 for systematic reviews and meta-analyses on *Helicobacter pylori* prevalence in China, using terms “*H. pylori*”, “*H. pylori*”, “prevalence”, “seroprevalence”, “epidemiology”, “China”, “systematic review”, and “meta-analysis” without language restriction. We identified six relevant meta-analyses reporting a decline in *H. pylori* prevalence in China, from approximately 55–60% to 40–45% between 1990 and 2020. However, these studies had important limitations. Most did not account for key sources of bias, including imperfect diagnostic accuracy and differences between seroprevalence and current infection, and none adequately characterised age-specific patterns, which are critical for an infection acquired predominantly in childhood. The influence of population ageing on overall population burden was also unknown. We also searched for studies that estimated the *H. pylori*-attributable disease burden in China. We found no studies that estimated age-cohort-specific force of infection (FOI) for *H. pylori* in China or quantified attributable burden using age-specific *H. pylori* prevalence.

**Added value of this study**

To our knowledge, this is the first study to estimate age-specific FOI for *H. pylori* in China and the first to jointly model current infection, seropositivity, and ever-infection data within a unified Bayesian framework for *H. pylori*. We developed and validated a Bayesian catalytic model that accounts for diagnostic misclassification, antibody waning, and period-varying treatment dynamics. We identified a marked reduction in childhood FOI among cohorts born after 1980, likely reflecting improved sanitation, water supply, and reduced household crowding following China's economic reforms. Despite declining childhood infection, overall population prevalence remained stable at approximately 40% through 2023. Using relative risks from the China Kadoorie Biobank and fully propagating parameter uncertainty, we estimated population attributable fractions and attributable cases for gastric cancer and peptic ulcer disease by subtype. Contrary to the more optimistic trajectory implied by earlier prevalence reviews, our results show that the absolute burden of *H. pylori*-attributable gastric cancer and peptic ulcer disease in China has remained high and changed little over the past three decades. In 2023, *H. pylori* contributed to 340,000–370,000 gastric cancer cases and 160,000–210,000 peptic ulcer disease cases.

**Implications of all the available evidence**

Despite declining childhood FOI, the absolute burden of *H. pylori*-attributable gastric cancer and peptic ulcer disease in China remains substantial and is unlikely to decline rapidly without active intervention. These findings support calls for more active *H. pylori* control in high-burden areas, including organised screen-and-treat strategies and family-based approaches, as reflected in recent international and national guidelines. This study provides evidence directly relevant to current national and international policy discussions on *H. pylori* control, and offers a modelling approach that can be applied to other countries.

## Introduction

Over 40% of adults worldwide are infected with *Helicobacter pylori* (*H. pylori*), the leading cause of gastric cancer (GC).^1^^,^^2^ *H. pylori* infection is typically acquired in childhood. Although transient infection or spontaneous clearance can occur, particularly in infants and young children, established infection generally persists throughout life unless eradicated.^3^ Most infections remain asymptomatic, so only a small fraction of people seek testing or receive eradication therapy.^4^ As a result, a large reservoir of chronic infection can accumulate silently, sustaining substantial preventable disease burden.^5^ The International Agency for Research on Cancer (IARC) estimated that in 2020 alone, about 850,000 cancer cases worldwide were attributable to *H. pylori* infection.^6^ This exceeds the burden from any other cancer-causing infection, including human papillomavirus and hepatitis B and C viruses.^6^ Beyond carcinogenesis, *H. pylori* infection is a main risk factor for peptic ulcer disease (PUD),^7^^,^^8^ further increasing the clinical and economic burden.^9^^,^^10^

China has one of the world's largest populations living with *H. pylori* infection and accounts for about 35% of global GC incident cases in 2024.^11^ Given the burden and the established benefits of eradication for preventing GC and PUD,^12^, ^13^, ^14^ international and Chinese guidelines have increasingly endorsed more active control, including population screen-and-treat of *H. pylori*.^6^^,^^7^^,^^15^ Designing such strategies requires more than a single pooled prevalence estimate. Policy planning depends on understanding how *H. pylori* prevalence varies by age, calendar period, and birth cohort, and how those patterns translate into preventable disease burden. However, current evidence remains insufficient.

*H. pylori* prevalence changes rapidly in childhood within birth cohorts and also differs substantially across cohorts at the same age.^3^ Yet most previous prevalence meta-analyses^2^^,^^16^ did not account for age structure when assessing temporal trends, and many did not extract detailed age-subgroup results from the original studies. In addition, published prevalence estimates are derived from heterogeneous outcome definitions and diagnostic approaches, including current-infection prevalence, seroprevalence, and ever-infection measures. Current-infection prevalence reflects active bacterial infection, whereas seroprevalence reflects detectable antibodies. Because antibodies can persist after clearance, seropositivity does not necessarily indicate active infection. Ever-infection measures aim to capture any current or previous infection. These quantities are distinct and should not be pooled as if they were interchangeable.

This study reconstructs age, period, and cohort patterns of *H. pylori* infection in Mainland China from 1990 to 2023 by jointly modelling current-infection prevalence, seroprevalence, and ever-infection data. We estimate the force of infection (FOI) and the prevalence of *H. pylori*, and quantify the attributable burden of GC and PUD, propagating uncertainty throughout the analysis. Together, these results aim to inform policies for *H. pylori* infection screening and treatment in Mainland China and provide a transferable modelling framework that could be adapted to other high-burden areas.

## Methods

### Study design

**Fig. 1:**
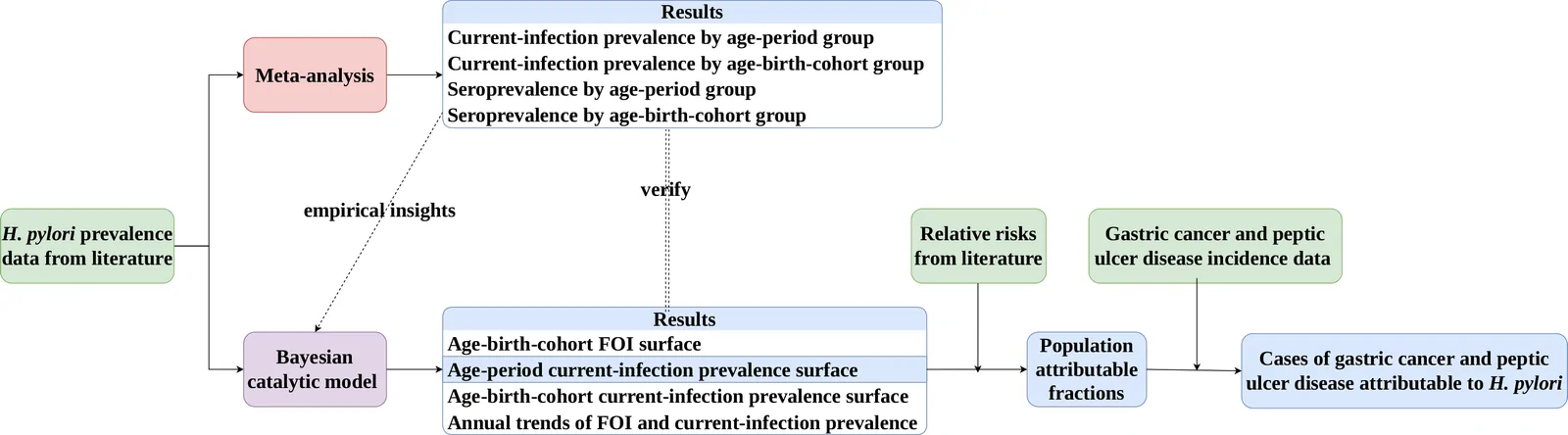
**Workflow of this study**.

We used a multi-step framework (Fig. 1). First, we performed a systematic search for studies reporting *H. pylori* prevalence in China. Second, we conducted meta-analyses to obtain empirical age-period and age-birth-cohort prevalence patterns. These insights informed a Bayesian catalytic model to estimate FOI and prevalence by age and calendar year while accounting for within- and between-study uncertainty and heterogeneity in detection methods across studies. Finally, we combined posterior estimates of current infection prevalence from the Bayesian model with literature-based relative risks and case counts to estimate the population attributable fraction (PAF) and the number of GC and PUD cases attributable to *H. pylori*. This study is reported in accordance with Guidelines for Accurate and Transparent Health Estimates Reporting (GATHER).^17^

### Data sources of *H. pylori* prevalence

#### Literature search

**Fig. 2:**
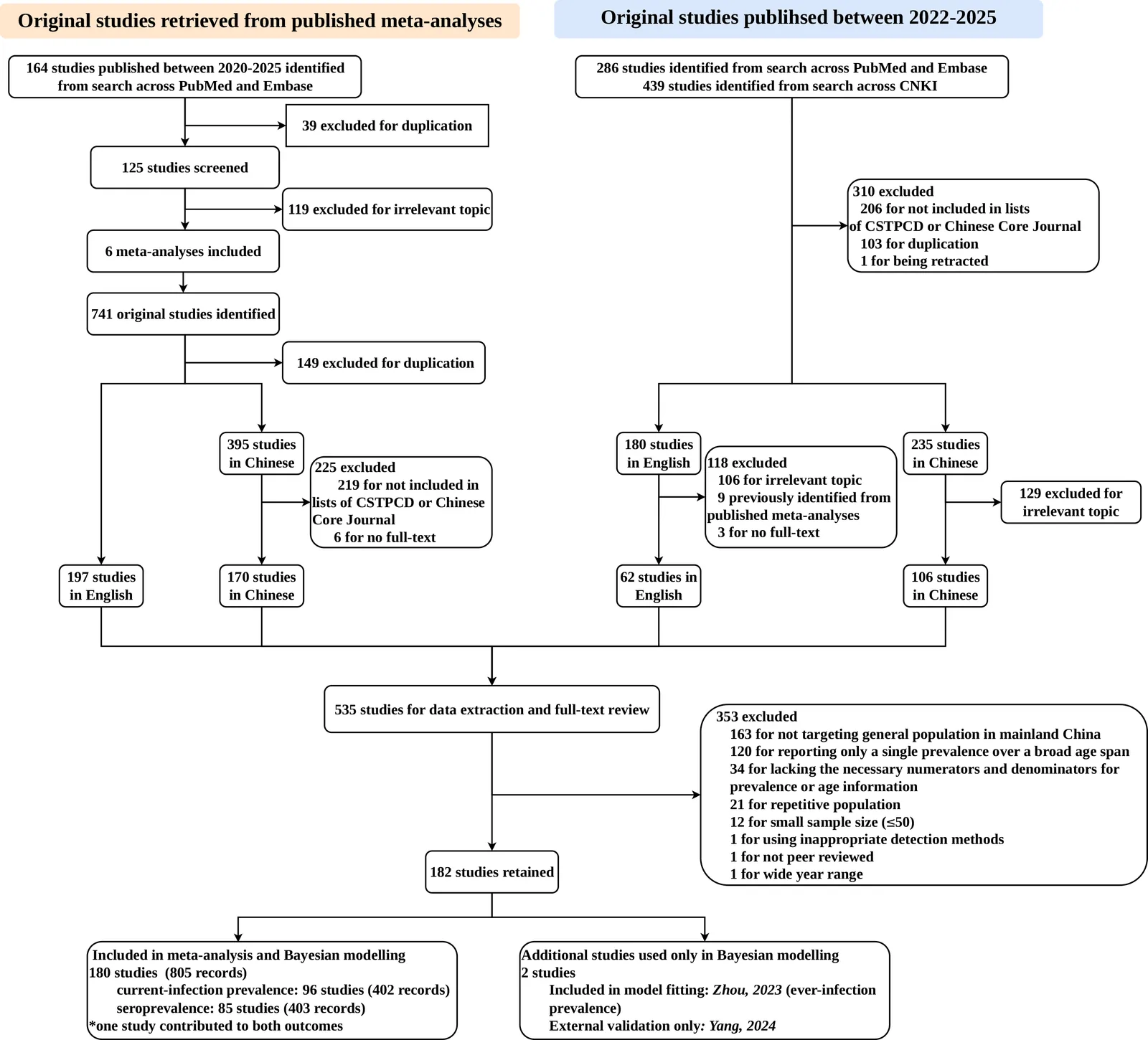
**Flowchart of literature selection.** Studies were identified from published meta-analyses and an updated database search. In total, 180 studies were included in the meta-analysis, with one additional study included in Bayesian model fitting and another reserved for external validation. CNKI, China National Knowledge Infrastructure; CSTPCD, China Scientific and Technical Papers and Citations Database.

We first searched PubMed and Embase for systematic reviews or meta-analyses reporting on *H. pylori* prevalence in China, restricting to publications from January 2020 to April 2025 (Fig. 2). The search strategies are in Table S1. We included reviews that reported prevalence data for the general population in China and provided sufficient detail on the original studies. Six meta-analyses^1^^,^^2^^,^^16^^,^^18^, ^19^, ^20^ were identified, and their characteristics are summarised in Table S2. From these meta-analyses, we extracted the original studies relevant to mainland China.

Since the most recent original studies covered by the included meta-analyses extended only through 2022, we performed an updated search of PubMed, Embase, and China National Knowledge Infrastructure (CNKI) for original studies on *H. pylori* prevalence in China published from 2022 to June 17, 2025. The search strategies for this updated search are listed in Table S3.

#### Initial selection

Two reviewers (D.S. and W.H.) independently conducted initial screening of titles and abstracts. Discrepancies were resolved by discussion. We included studies reporting *H. pylori* prevalence in populations from mainland China. We excluded studies reporting only *H. pylori* antibiotic resistance or *H. pylori* incidence, as well as studies without full text. To ensure minimum reporting and publication standards, Chinese-language papers were eligible only if published in journals indexed in the China Scientific and Technical Papers and Citation Database (CSTPCD) or Chinese Core Journal Database.

#### Data extraction

For each included study after initial selection, we used a semi-automated workflow with Gemini 2.5 Pro (Vertex AI) to extract study metadata, detection methods, age-specific prevalence, and subgroup data from full-text PDFs and stored them in structured tables. We then performed targeted checks for three methodological items: whether age-specific data were available only in Supplementary Materials, whether prevalence estimates had been adjusted for diagnostic accuracy, and how prior *H. pylori* eradication therapy was handled. Prompts, extraction rules, and quality-control procedures are provided in the Supplementary Methods.

#### Record selection and quality assessment

Two reviewers (D.S. and W.H.) independently conducted record selection based on the extracted data. We included only community-based and routine health examination surveys in mainland China to represent the general population, and excluded records that: 1) targeted selected occupational groups or patients with digestive diseases or symptoms; 2) used inappropriate detection methods (through saliva, gastric juice, or urine); 3) lacked the necessary numerators and denominators for estimating prevalence; 4) covered survey periods of 10 years or longer; 5) reported prevalence over a broad age span only and lacked details on age-specific estimates (See Supplementary Methods for prespecified age-band criteria). We further restricted to records with age point ≤100, a practical upper boundary for the population relevant to *H. pylori* burden control, and sample size >50. All extracted data were manually checked. The quality of each study was assessed using a risk of bias tool for prevalence studies developed by Hoy et al. (Table S4).^21^

### Meta-analysis of current infection prevalence and seroprevalence

We conducted separate meta-analyses for current-infection prevalence and seroprevalence (defined in Table S5). To capture temporal and birth-cohort patterns, we conducted separate meta-analyses for each outcome stratified by age-period and age-birth-cohort strata. Within each stratum, study-specific prevalence was pooled on the logit scale using inverse-variance random-effects models. Pooled logits and 95% confidence intervals were back transformed to the proportion scale.

To account for varying sensitivity and specificity of different detection methods (Table S5), we also adjusted each study's observed prevalence for the diagnostic accuracy using the Rogan-Gladen approach.^22^ Details on the adjustment method are provided in the Supplementary Methods. The corrected prevalence and its standard error were then pooled using the same approach described above.

Between-study heterogeneity was quantified using the between-study variance (*τ*^*2*^) and the *I*^*2*^ statistic. We reported the meta-analysis results for both reported and diagnostic-accuracy-corrected prevalence. Analyses were conducted with epiR, metagen, and metaprop packages in R 4.3.2.

### Bayesian estimation of *H*. *pylori* infection dynamics

We developed a Bayesian catalytic model to infer age- and period-specific dynamics of *H. pylori* infection. The model estimated the FOI for each birth cohort, defined as the number of new infections per susceptible person-year, and simulated transitions as each cohort aged. Individuals began uninfected (U), could become infected (I), could clear infection after treatment and remain seropositive (C) for a period, and could eventually serorevert^23^, ^24^, ^25^ (N) (Figure S1). The resulting state trajectories provided age-specific probabilities of current infection, seropositivity, and ever infection. These predictions were mapped to each study record according to age, survey year, prevalence outcome, and diagnostic method. Model parameters were estimated by fitting the predictions to the corresponding record-level numerators and denominators extracted from individual studies.

The model incorporated age-birth-cohort-specific FOI, period-varying treatment hazards, a constant antibody waning rate ω, and diagnostic misclassification. Let *a* denote age and *c* the birth cohort. As validated by Alarid-Escudero et al.^26^*, H. pylori* cumulative infection function for cohort *c* could be parameterised as:

$$F\left(a,c\right)=\alpha_{c}\left[1-e^{-\gamma_{c}a}\right]$$

where the asymptote $\alpha_{c}$ represents cohort-specific upper bound on the proportion ever infected and $\gamma_{c}$ governs how rapidly infection accumulates with age among those who are eventually infected. The implied FOI is

$$\lambda \left(a,c\right)=\frac{\alpha_{c}{\gamma_{c}e}^{-\gamma_{c}a}}{{1-\alpha_{c}+\alpha }_{c}e^{-\gamma_{c}a}}$$

**Fig. 3:**
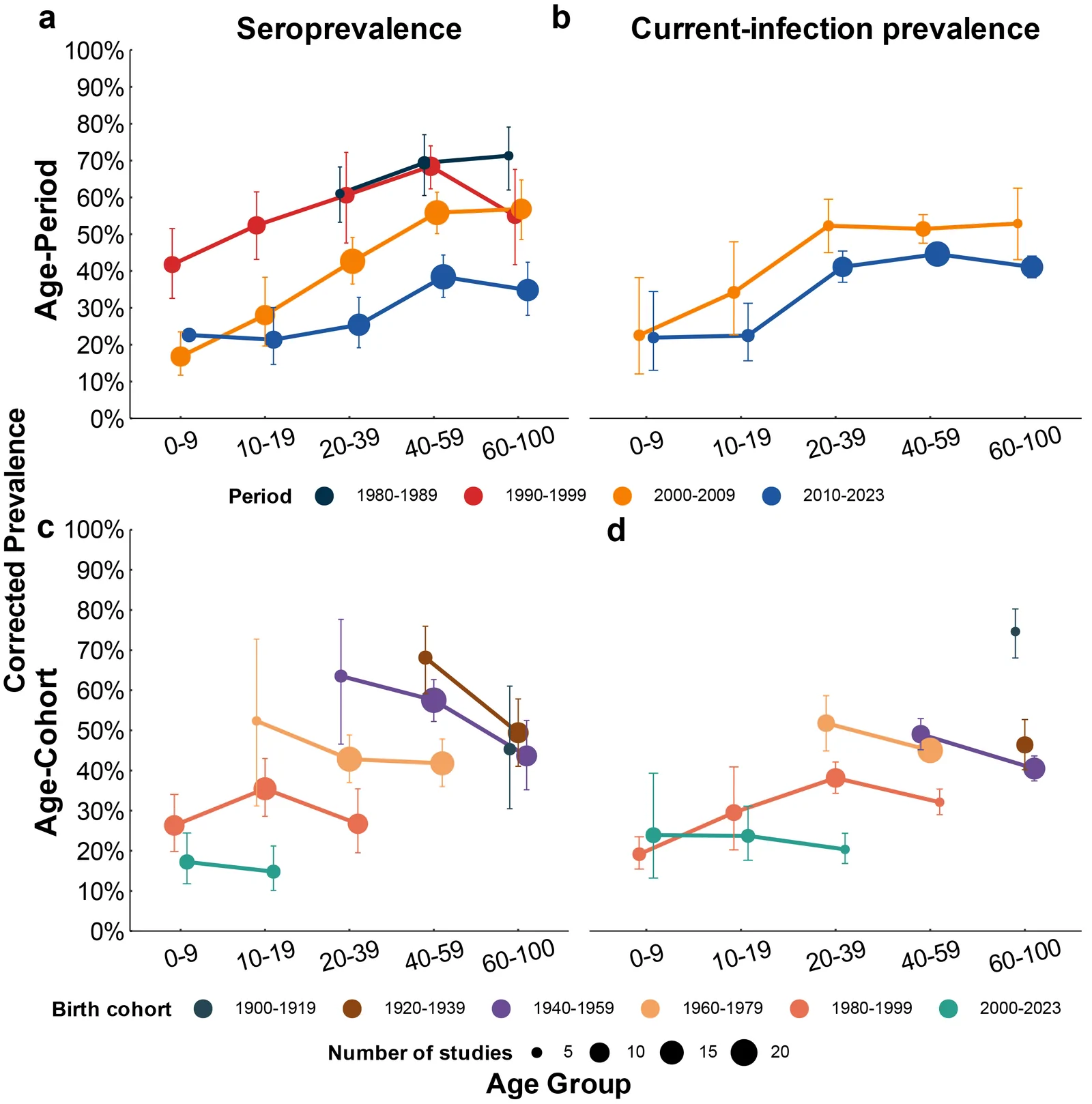
**Age-period and age-cohort patterns of *Helicobacter pylori* prevalence in China, 1980–2023.** Panels a and b show pooled age-period-specific seroprevalence and current-infection prevalence, respectively, estimated through meta-analysis after correction for diagnostic test accuracy. Panels c and d show corresponding pooled age-cohort-specific estimates. Lines represent pooled prevalence trends for each survey period (a, b) or birth cohort (c, d). Points indicate pooled estimates and error bars represent 95% confidence intervals. Point sizes are proportional to the number of studies contributing to each estimate.

The results of preceding meta-analysis (Fig. 3) informed the structural specification of the cohort trend: later birth cohorts had progressively lower maximum fractions ever infected. We therefore modelled the asymptote $\alpha_{c}$ as a monotonic decreasing function of birth cohort, while constraining γ to be constant across cohorts for identifiability.

We developed four competing Bayesian catalytic models (Figure S1) that differed in two structural dimensions: (A) the rate from infection to treatment, parameterised as either infection-time-independent or infection-time-dependent; and (B) the cohort-specific asymptote $\alpha_{c}$, parameterised using either monotone piecewise-linear or I-spline. Mathematical details of these model structures and model priors are provided in the Supplementary Methods.

Observed positives and totals were modelled with a beta-binomial likelihood to account for extra noise within studies. Additionally, a study-level random effect was applied on the logit of the true prevalence to account for between-study heterogeneity. Models were fitted using Hamiltonian Monte Carlo (No-U-Turn sampler) and evaluated using Pareto-smoothed importance sampling leave-one-out cross-validation (PSIS-LOO) for predictive performance and parsimony. Convergence was assessed using rank-normalised (R-hat <1.01), effective sample sizes, and divergent-transition diagnostics. Sampling settings and model comparison are described in the Supplementary Methods. If PSIS-LOO did not clearly discriminate among the four models, we selected the model combining an I-spline cohort asymptote with an infection-time-dependent treatment rate as the default. The I-spline avoids abrupt slope changes at fixed knots, and the infection-time-dependent specification reflects that treatment-seeking is driven by symptom development (Supplementary Methods).

For the preferred model, posterior summaries were obtained from all retained draws after convergence. To assess parameter identifiability within the selected model, we calculated pairwise Pearson correlations across joint posterior draws. Model fit was assessed by comparing predicted and observed prevalence by outcome type and age-period group. Additionally, external validation was performed against age-specific prevalence from a nationwide survey in mainland China.^27^ This large-scale survey had a stratified, multi-stage sampling method, ensuring good representativeness of the population. The nationwide survey reported only age-specific prevalence estimates without the corresponding numbers tested and positive. It was therefore excluded from both the preceding meta-analysis and all stages of model development and was used solely for independent external validation.

We then derived age- and cohort-specific FOI and age- and period-specific current-infection prevalence surfaces by integrating the model equations over age. Age- and cohort-specific current-infection prevalence was also summarised. Annual age-specific population counts for China from 1980 to 2023 were retrieved from the Global Burden of Disease (GBD) 2023 database.^28^ Using the population counts and the posterior prevalence estimates, we calculated the annual current-infection crude prevalence for children (<20 years), adults (≥20 years), and the total population. Age-standardised prevalence was calculated using the World Health Organization (WHO) world standard population.^29^

For all model-based estimates, we drew 5000 posterior samples and report posterior medians with 95% credible intervals. All analyses were conducted with cmdstanr, posterior, and loo packages in R 4.3.2.

### Estimating *H*. *pylori* attributable disease burden

We estimated the *H*. *pylori* attributable disease burden for the population aged 20 or older. Previous studies suggested varying effect sizes of *H. pylori* infection across GC subtypes (cardia and non-cardia)^30^ and PUD (gastric ulcers (GU) and duodenal ulcers (DU)).^31^ Therefore, we estimated the age- and period-specific PAF for each disease subtype separately using Levin's formula.

$${PAF}_{a,t,k}=\frac{I_{a-L,t-L}\left({RR}_{k}-1\right)}{I_{a-L,t-L}\left({RR}_{k}-1\right)+1}$$

where *k* indexes subtype (GC: cardia, non-cardia; PUD: GU, DU), $I_{a-L,t-L}$ is the current-infection prevalence in the corresponding lagged age-period cell. We assumed a latency of 10 years for GC and 5 years for PUD. These lags were chosen to approximate the exposure windows most relevant to etiologic risk estimation: for GC, evidence suggests that *H. pylori* measured at least 10 years before diagnosis better captures the relevant infection history,^32^ whereas ulcer outcomes respond more quickly to infection status. $RR_{k}$ is the subtype-specific relative risk (RR). Because *H. pylori* associations with gastric cancer, especially by subsite, vary across regions,^33^ we used China-specific RRs of *H. pylori* infection for cardia and non-cardia GC,^34^ GU, and DU^31^ from the China Kadoorie Biobank, one of the largest prospective cohorts in China (Table S6).

Age-specific numbers of incident cases with 95% uncertainty intervals (UIs) of GC and PUD in China from 1990 to 2023 were extracted from the GBD study 2023.^28^^,^^35^ However, age-, period, and subtype-specific incidence data for GC were not available. Aggregated data from the National Cancer Registry of China indicate that non-cardia GC represents 50%–70% of GC cases in 2010s.^36^^,^^37^ For PUD, the proportion of cases that are DU was about 50%–70%, based on large-scale Chinese studies.^27^^,^^38^ Accordingly, we considered two subtype-mixing scenarios for GC (50/50 and 70/30 non-cardia/cardia) and two for PUD (50/50 and 70/30 DU/GU). Subtype proportions were held constant over time within each scenario. Each case was assigned to only one subtype with proportions summing to one.

With the age-period- specific incident case numbers and the PAFs for each subtype, we calculated the age-period-subtype- specific attributable cases as follows:

$$A_{a,t,k}={\text{PAF}}_{a,t,k}\times C_{a,t,k}$$

$$A_{\text{overall}}\left(t\right)=\sum_{k}\sum_{a}A_{a,t,k}\text{.}$$

$${\text{PAF}}_{t,k}=\frac{\sum_{a}A_{a,t,k}}{\sum_{a}C_{a,t,k}}$$

Where $C_{a,t,k}$ refers to the incident cases for a specific age, period, and disease subtype. We propagated uncertainty from 1) posterior draws of age-period prevalence, 2) uncertainty in subtype-specific RRs, and 3) the reported uncertainty intervals for age-specific case counts in GBD by Monte Carlo sampling (5000 draws). These results were summarised as medians and 95% UIs. To separate the influence of changes in the population age structure, we calculated PAFs with WHO standard population weights.^29^

### Sensitivity analysis

We repeated the full analysis using an alternative catalytic model (Figure S1) that changed 1) the functional form for the cohort asymptote $\alpha_{c}$ (monotone piecewise-linear vs. monotone I-spline) and 2) the clearance specification (constant vs. infection-time-dependent). We recomputed FOI, prevalence surfaces, PAFs, and attributable cases following the same procedure as in the main analysis.

### Ethics approval

Ethics approval and individual informed consent were not required because this study used only previously published, aggregate, non-identifiable data.

### Role of the funding source

The funders of the study had no role in study design, data collection, data analysis, data interpretation, or writing of the report.

## Results

### Characteristics of included studies

From six published meta-analyses, 741 original studies were initially identified, yielding 197 English-language and 170 Chinese-language studies after initial selection (Fig. 2). An updated search for original studies published between 2022 and 2025 identified 725 studies; after initial screening, 62 English and 106 Chinese studies remained. After combining both sources and removing duplicates, 535 studies underwent full-text review. Of these, 353 were excluded because they did not target the general population in mainland China or for other prespecified reasons.

After full-text review, 180 unique studies (805 records) were eligible for the meta-analysis. These comprised 96 studies contributing 402 current-infection records and 85 studies contributing 403 seroprevalence records; one study contributed both outcome types. In addition, one study^39^ reporting ever-infection prevalence based on combined urea breath test and recalled eradication history was included only in the Bayesian catalytic model, bringing the total number of studies in Bayesian model fitting to 181. A separate nationwide survey^27^ lacked numerators and denominators and was therefore excluded from the meta-analysis and Bayesian model fitting, but was used as an external validation target.

Among these studies, birth years spanned 1911–2018, ages ranged from 0 to 93, and survey year covered 1983–2023 (Table S7). All 31 provinces in Mailand China were covered (Table S8). All surveys conducted in the earlier years (1980–2000) relied on serology tests. Under our inclusion criteria, all included studies met the internal-validity items and were therefore assessed as having low risk of bias for this domain (Table S4). In contrast, most studies (179/181) were judged to have high risk of bias for at least one external-validity item (Table S9).

### Meta-analysis of *H. pylori* prevalence

Our meta-analysis of corrected prevalence showed significant temporal changes in both seroprevalence (Fig. 3a) and current-infection prevalence (Fig. 3b), particularly after 2000. Seroprevalence increased with age in all survey periods. In earlier periods (1980–2000), adult seroprevalence was approximately 60%, with a gradual decline in more recent years. By 2010–2023, about 40% of adults were infected (Table S10).

In the age-cohort perspective, seroprevalence no longer rose continuously with age. Instead, it remained broadly stable after age 20 within each birth cohort, and for cohorts born between 1900 and 1960 it declined at older ages (Fig. 3c). Later birth cohorts reached a lower peak prevalence than earlier cohorts.

Between-study heterogeneity was high in most age-period-by-outcome and age-cohort-by-outcome strata, with I^2^ above 50% (Table S10). The overall patterns remained consistent when we examined the reported prevalence without being corrected for diagnostic misclassification (Figure S2, Table S11).

### Estimations of *H*. *pylori* infection dynamics

The four competing Bayesian catalytic models achieved similar predictive performance (Table S12). All four models showed satisfactory convergence, with maximum rank-normalised R-hat values ranging from 1.0012 to 1.0044 and no divergent transitions. Models 3 and 4 had the lowest effective number of parameters, suggesting that their shared infection-time-dependent treatment structure may have reduced effective flexibility and sensitivity to individual observations. Model 4 was selected based on its comparable predictive performance, smooth I-spline cohort specification, and biologically plausible treatment structure.

The selected model reproduced the observed prevalence data well. Model predictions generally aligned well with observed data across age groups, periods, and outcome types (Figure S3). For the external validation, the model predictions were closely aligned with the observed data across all age groups (Figure S4). For most age groups, the model 95% CrIs also fell within the observed 95% confidence intervals, indicating a good fit. Moderate posterior correlations were observed between the timing and maximum rate of treatment scale-up and between the late-cohort asymptote and age-related acquisition rate, indicating partial parameter identifiability (Figure S5).

**Fig. 4:**
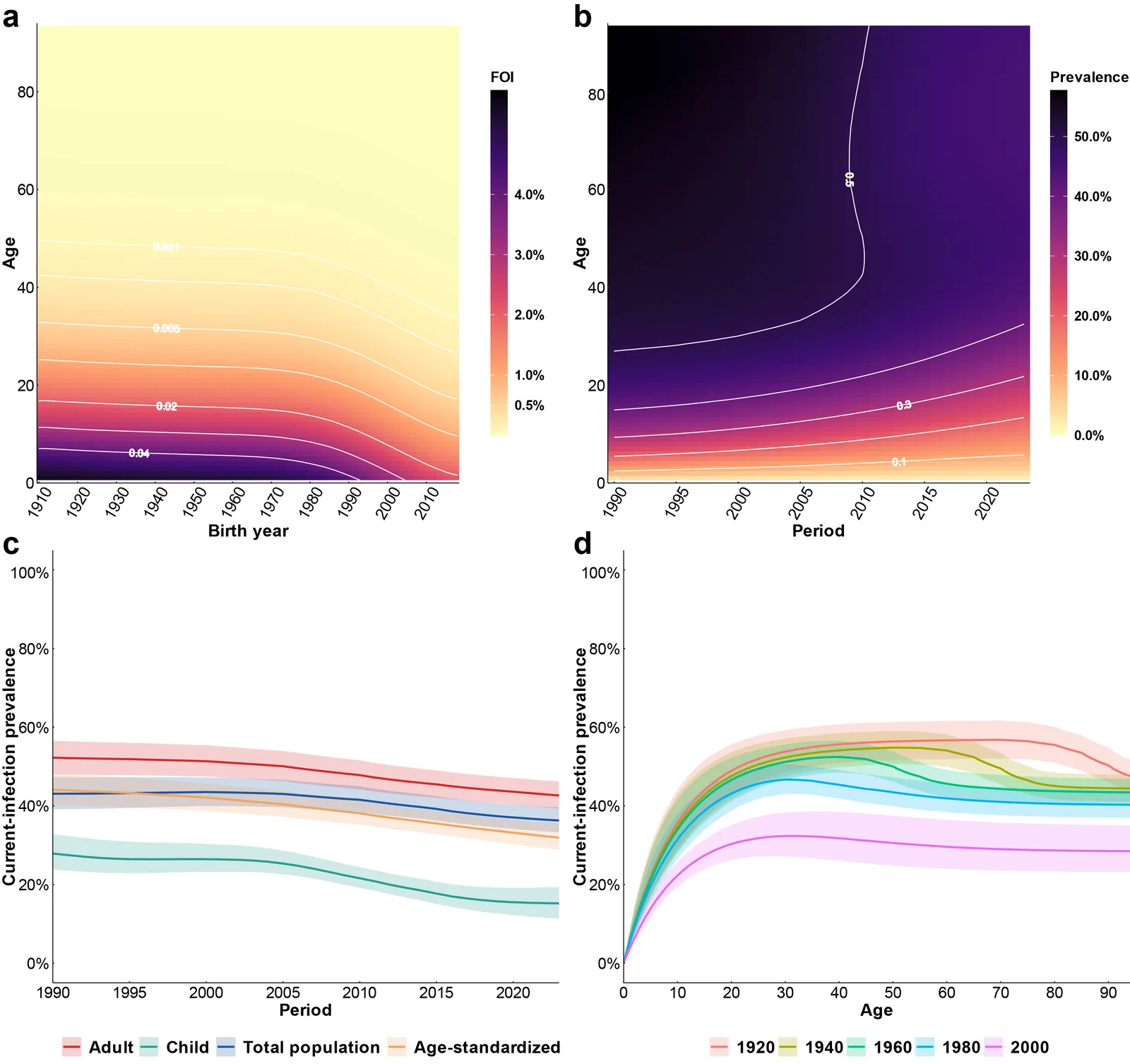
**Age-birth cohort-specific***Helicobacter pylori***force of infection, age-period-specific current-infection prevalence, annual current-infection prevalence, and age-birth cohort-specific current-infection prevalence in China.** Panel a shows posterior median FOI, where colours indicate the FOI (% per year), and white contour lines mark FOI levels (labelled). Panel b shows posterior median current-infection prevalence, where colours show the proportion infected, and white contour lines mark prevalence levels (labelled). In panels c and d, lines represent posterior medians of current-infection prevalence and shaded ribbons indicate 95% credible intervals.

Higher FOI rates were observed in earlier birth cohorts and younger ages (Fig. 4a). For the 1920 cohort, annual FOI declined from 3.21% at age 10 to 1.56% at age 20 and 0.27% at age 40. Corresponding estimates for the 2000 cohort were 1.72%, 0.75%, and 0.12%, respectively. A marked shift was apparent after the 1980 birth cohort, with substantially lower FOI in childhood than in earlier cohorts. FOI in the youngest ages of recent birth cohorts was similar to that observed at age 20 among cohorts born in the 1910s.

In the calendar-period analysis, the model revealed a clear decline in current-infection prevalence among children after the year 2005, whereas adult and overall population prevalence changed more modestly (Fig. 4b and c). Overall population prevalence declined from 43.1% (95% CrI 39.1, 47.4) in 1990 to 36.3% (33.2, 39.6) in 2023. Age-standardised prevalence declined more than the crude prevalence. In the birth-cohort view, the model estimated that current-infection prevalence rose before age 20 and then remained stable, with declines at older ages among cohorts born in the 1920s and 1940s (Fig. 4d). This trend was consistent with findings from our meta-analysis (Fig. 3c). In contrast, for the latest birth cohorts born after 1980, the reduction was concentrated in younger ages.

### *H*. *pylori* attributable disease burden

**Fig. 5:**
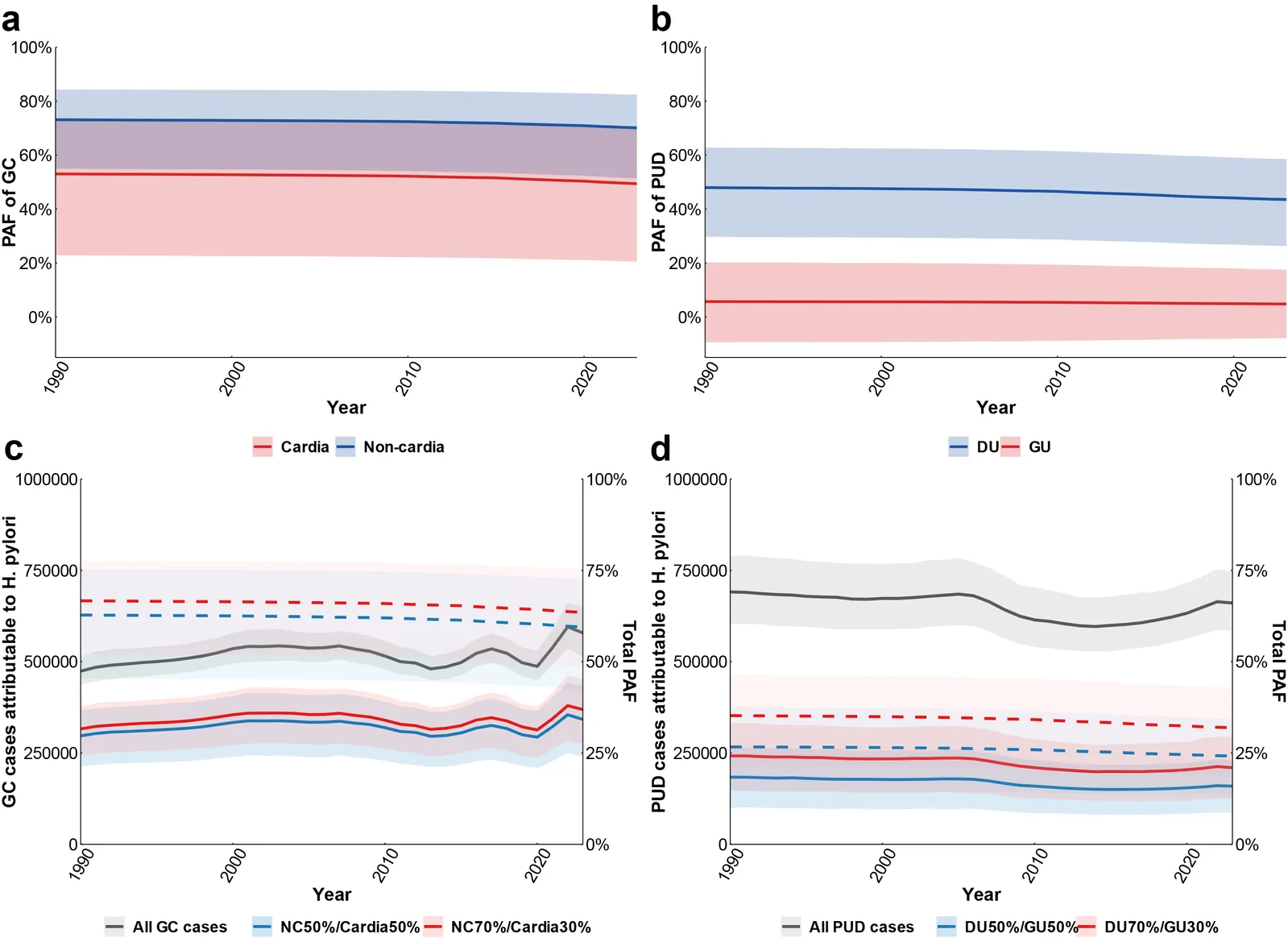
***Helicobacter pylori*-population attributable fractions and cases for gastric cancer and peptic ulcer disease in China, 1990–2023.** Panels a and b show median PAFs (solid lines) with 95% uncertainty intervals (shaded ribbons) for gastric cancer (GC) by subsite, and peptic ulcer disease (PUD) by subtype. Panels c and d show median numbers (solid lines) with 95% uncertainty intervals (shaded ribbons) of cases attributable to *H. pylori* for GC and PUD. Grey line shows total cases. Coloured solid/dashed lines show attributable cases/PAF under different scenarios of subtype composition. The 95% credible interval of the PAF for gastric ulcers includes 0 because the 95% confidence interval for the relative risk of *H. pylori* includes 1. PAF, population attributable fraction; GC, gastric cancer; PUD, peptic-ulcer disease; DU, duodenal ulcer; GU, gastric ulcer; NC, non-cardia.

Reflecting the stable trends in *H. pylori* prevalence in the total population before 2010 (Fig. 4c), the PAFs for both non-cardia and cardia GC showed little temporal variation (Fig. 5a). In 2023, the PAF was 70.1% (95% UI: 51.4%, 82.4%) for non-cardia GC and 49.4% (20.5%, 70.7%) for cardia GC (Fig. 5a). Under the two bounding subtype-mixing scenarios, estimates of attributable GC burden were stable over time, ranging from 341,870 (95% UI: 240,131, 434,064) to 368,995 (274,495, 452,764) cases in 2023 (Fig. 5c).

For PUD, PAFs for both DU and GU declined slightly over time, consistent with the more rapid response of PUD epidemiology to changes in *H. pylori* prevalence (Fig. 5b). In 2023, the PAF was 43.5% (95% UI: 26.3%, 58.5%) for DU and 4.8% (−7.8%, 17.5%) for GU. Under the 50:50 and 70:30 case-mix scenarios, respectively, attributable DU cases were 143,836 (95% UI: 85,563, 200,318) and 201,001 (117,997, 277,457), whereas GU cases were 15,866 (−25,715, 58,212) and 9241 (−16,317, 35,189); both GU intervals included zero. Across these scenarios, the combined PUD burden was approximately 160,000–210,000 cases (Fig. 5d).

### Sensitivity analysis

The patterns of *H. pylori* infection dynamics observed in the alternative model remained generally consistent with those of the primary model (Figure S6). Both models showed a noticeable transition in the FOI after the 1980s, but the decline was sharper in the alternative model, likely due to the piecewise-linear specification (Figure S6). For current-infection prevalence, the alternative model indicated modestly higher infection rates for older age groups in earlier years. Besides, it showed a steeper decline in current-infection prevalence for these age groups after 2005 compared to the primary model (Figure S6).

The largest differences between the two models appeared in regions with limited observed data, such as ages above 20 years for the 2000 birth cohort and ages above 40 years for the 1980 birth cohort. In these regions, the alternative model predicted a consistently steeper decline in current-infection prevalence across birth cohorts (Figure S6). Despite these differences in extrapolated prevalence patterns, estimates of PAFs and attributable cases for GC and PUD were similar in the two models (Fig. 5, Figure S7).

## Discussion

This study combined age-period and age-cohort stratified meta-analysis with a Bayesian catalytic model to reconstruct age-, period-, and cohort-specific dynamics of *H. pylori* infection in China. First, both the meta-analysis and the Bayesian model indicated that most infections occurred in childhood, with FOI declining substantially and remaining low in adulthood. Second, cohorts born after 1980 had lower infection prevalence. Third, despite declining infection among children and adolescents, overall adult prevalence remained around 40%. The *H. pylori*-attributable burdens of GC and PUD remained substantial, with an estimated 340,000–370,000 GC cases and 160,000–210,000 PUD cases in 2023. The estimated PUD burden primarily reflected DU, whereas the GU-specific burden remained compatible with zero. Given the stable adult prevalence and population ageing through the 2030s, absolute *H. pylori*-attributable GC and PUD cases are unlikely to decline rapidly without active interventions. Findings from both the meta-analysis and the Bayesian transmission model were consistent, and the model results were robust across different model specifications.

From a calendar-period perspective, our model suggested a slower and more modest decline than previous meta-analyses in China.^2^^,^^16^^,^^19^ One possible reason is that earlier prevalence surveys in the 1990s were disproportionately conducted among adults and relied more on serology, whereas more recent studies increasingly included younger age groups and used other detection methods. This shifting composition of the evidence base over time can exaggerate the apparent downward trend in pooled estimates. This pattern is also visible in the underlying data summarised in prior meta-analyses,^16^ but is rarely discussed or accounted for. We did observe a clear reduction in infection among children and adolescents after the mid-2000s, but adult prevalence remained broadly stable. After age standardisation, the decline was more apparent, indicating that population ageing helped sustain the crude population-level burden.

From a birth-cohort view, the meta-analysis indicated that prevalence was largely flat after age 20, and the model estimated that FOI declined substantially and remained low throughout adulthood. This aligns with prior evidence that most infections are acquired in childhood.^3^^,^^26^^,^^40^, ^41^, ^42^ For cohorts born in 1900–1960, both meta-analysis and Bayesian model suggested some decline in prevalence at older ages, which may partly reflect the scale up of *H. pylori* eradication therapy since 1990s.^6^

We also observed a clear reduction in FOI among individuals born after 1980. China's reform and opening-up policy, beginning in 1978, was followed by rapid improvements in living conditions, including water supply, reduced household crowding,^43^ and improved hygiene practices in households and schools.^44^ These structural changes likely reduced close-contact *H. pylori* transmission in childhood, resulting in lower lifetime infection fractions in later birth cohorts. These findings suggest that modernisation can reshape *H. pylori* transmission dynamics even before medical interventions reach scale.

These cohort gains in *H. pylori* prevalence reduction, however, have not yet translated into a sharp decline in population-level *H. pylori* attributable disease burden. Because GC and PUD risk is concentrated at older ages, the benefits of reduced childhood acquisition are delayed, and demographic ageing increases the contribution of older, highly infected cohorts. As a result, adult prevalence remains high and the absolute *H. pylori*–attributable burden is sustained despite improving infection dynamics in children. More active control of *H. pylori* infection could reduce this burden. The benefits of *H. pylori* eradication in preventing GC and PUD are well-established.^12^, ^13^, ^14^ Both Chinese and international guidelines increasingly recommend population screening and treatment for *H. pylori* in appropriate settings.^6^^,^^7^^,^^15^ Although China lacks a national policy on *H. pylori* control, a recent Chinese expert consensus recommends a family-based screen-and-treat approach, which may be more efficient and practical.^15^ South Korea is conducting a large-scale trial within the National Cancer Screening Program to assess the efficacy of *H. pylori* treatment in reducing GC risk in the general population,^45^ and Japan now reimburses eradication therapy for patients with endoscopically confirmed *H. pylori*-associated gastritis.^46^ Western countries with relatively low *H. pylori* prevalence are also becoming more active in *H. pylori* control. The European Council has suggested that European Union countries with a high GC burden consider screening and treatment for *H. pylori.*^47^ The American College of Gastroenterology guideline for *H. pylori* treatment recommended screen-and-treat for high-risk racial groups.^48^

These diverse approaches underscore that implementing a population-level strategy requires careful design.^6^ Our age- and birth-cohort-specific prevalence estimates do not directly determine the optimal programme design, but they provide the baseline inputs needed to evaluate it. First, they identify which cohorts currently carry the greatest infection burden and therefore where screening is most likely to detect infections. Second, when combined with evidence on GC natural history and age-specific eradication benefits, these estimates allow decision models to project the downstream health effects of screening at different ages. Third, they also determine the expected number of individuals eligible for treatment under each strategy, which is critical for estimating antibiotic use, resistance-related concerns,^49^^,^^50^ programme costs, and budget impact. In this way, our study provides the epidemiological foundation on which modelling can compare candidate strategies and quantify their benefits, harms, costs, and uncertainty.^51^^,^^52^

This study has several limitations. First, China has substantial geographical variation in *H. pylori* prevalence, disease burden, and healthcare access, and substantial between-study heterogeneity was observed. Although the random-effects meta-analysis and the Bayesian catalytic model's study-level random effects and beta-binomial likelihood accommodated this variability statistically, they could not explain its sources or eliminate potential selection and measurement biases. Our estimates represent national averages and may therefore mask important variation between regions or ethnicities. They should not be used directly to determine regional resource allocation. Such decisions will require dedicated analyses using systematically collected, locally representative data tailored to the relevant policy context. Second, although we accounted for broad differences in diagnostic accuracy and distinguished seropositivity from current infection, assay-specific performance, evolving laboratory procedures, and cut-off values were not consistent across the study period.^53^ Residual measurement error cannot be excluded. Third, some parameters were only partially identifiable; the resulting uncertainty and posterior dependence were reflected in the credible intervals and propagated into the uncertainty intervals. Separately, the available data could not clearly distinguish alternative structural assumptions about treatment dynamics because cohorts born around the introduction of eradication therapy are only now entering middle age. Sensitivity analyses indicated that alternative assumptions did not materially affect our results, but this limitation could affect long-term projections, which we did not undertake. Fourth, *H. pylori* infection and nonsteroidal anti-inflammatory drugs (NSAIDs) use have been shown to have a synergistic interaction to increase the risk of PUD.^8^ Our estimation of PAF on PUD did not incorporate the temporal trends of NSAIDs use in China, which may lead to an underestimation of the attributable burden. Fifth, incidence data by anatomical subtype were not available. We therefore used bounding assumptions for GC (cardia/non-cardia) and PUD (GU/DU) to estimate the attributable disease burden, which introduced additional uncertainty. These scenarios did not capture temporal changes in subtype composition, which could affect the exact burden estimates. However, any time-varying composition within the assumed bounds would produce estimates between the two scenario curves, which showed limited separation and similar temporal trends.

This study is China-specific, yet the findings are likely to generalise beyond China. Modernisation since the 1980s has lifted China from a low-income to an upper-middle-income country, making its experience informative for other countries undergoing similar transitions. For low-income countries today, investments in water and sanitation, and reduced household crowding are expected to produce durable declines in *H. pylori* transmission and to enhance the effectiveness of eradication therapy.^3^ At the same time, interpretation of recent trends must account for age structure. Much of the prior optimism about *H. pylori* in China was from aggregated rates that did not account for age structure. Our results offered a clearer view. Infection in children is declining, whereas adults remain highly infected, and demographic ageing sustains a high absolute burden. This pattern is likely to hold beyond China, especially in East and Southeast Asia, where rapid socioeconomic developments have lowered infection in younger cohorts,^54^ while large, highly infected adult cohorts continue to age into the GC risk window.^55^

Using fine-grained age- and period-specific prevalence data, our study applied a Bayesian catalytic model that integrates current infection, seropositivity, and ever-infection data accounting for diagnostic misclassification and antibody waning, thereby providing a more complete picture of *H. pylori* dynamics than prevalence pooling alone. Model estimates were corroborated by findings of our age-period and age-cohort meta-analyses and validated with an external high-quality national study. Our study can inform planning for *H. pylori* infection screen-and-treat in Mainland China and provide a methodological framework for estimating *H. pylori* dynamics in other high-risk areas.

## Contributors

DS, ILV, and JH conceptualised the study. DS and WH collected the data; DS curated the data; DS and WH analysed the data; and FAE, JRC, SW, and DT interpreted the data. DS and WH were involved in data visualisation. FAE, RJH, UL, MS, MCC, JYP, WC, ILV, and JH provided supervision. DS and ILV acquired funding. DS wrote the original draft of the manuscript, which was reviewed and edited by all authors. All authors critically revised the article for important intellectual content. DS, WH, and JH accessed and verified the data. All authors had full access to all the data in the study and had final responsibility for the decision to submit for publication.

## Data sharing statement

The extracted *H. pylori* prevalence input dataset and the model output estimates are provided in Appendix. Data on gastric cancer and peptic ulcer disease are publicly available in the Global Burden of Disease results portal (https://vizhub.healthdata.org/gbd-results/). Analytic code used to generate the estimates is available from the corresponding author upon reasonable request.

## Disclaimer

The contributions of the authors who are affiliated with the National Institutes of Health (NIH) are considered works of the U.S. government; however, the findings and conclusions presented in this article are those of the authors and do not necessarily reflect the views of the NIH or the U.S. Department of Health and Human Services. Where authors are identified as personnel of the International Agency for Research on Cancer/World Health Organization, the authors alone are responsible for the views expressed in this article and they do not necessarily represent the decisions, policy, or views of the International Agency for Research on Cancer/World Health Organization.

## Declaration of generative AI and AI-assisted technologies

During the preparation of this work the authors used ChatGPT 5.4 to polish the language, generate code for data visualisation, and improve the overall clarity of the text. The authors used Gemini 2.5pro to extract information from included original studies. After using this tool, the authors reviewed and edited the content as needed and take full responsibility for the content of the published article.

## Editor note

The translated Abstract in Chinese was submitted by the authors and we reproduce it as supplied. It has not been peer reviewed. Our editorial processes have only been applied to the original abstract in English, which should serve as reference for this manuscript.

## Declaration of interests

UL reports personal consulting fees from Guardant, Freenome, Neptune Medical, Leerink/Medacorp, William Blair, Guidepoint, Medial EarlySign, Geneoscopy, Medtronic, Kohler Ventures, and SanguiBio, and holds unexercised stock options in Universal Dx, Lumiinus, Cylinder Health, and SanguiBio, all outside the submitted work. He declares no other competing interests. MS reports participation in a data safety monitoring board or advisory board for the GISTAR study, outside the submitted work. She declares no other competing interests. FAE reports research funding to his institution from the NCI Cancer Intervention and Surveillance Modeling Network gastric-cancer program, outside the submitted work. He declares no other competing interests. The other authors declare no competing interests.

## Appendix A Supplementary data

Appendix Clean

Abstract in Chinese
