# Supplementary material for: Trends in Helicobacter pylori prevalence, force of infection, and attributable disease burden in China: a systematic review and Bayesian modelling study
Source: Lancet Reg Health West Pac. 2026 Sep 10;74:101979. doi: 10.1016/j.lanwpc.2026.101979 (PMC13584135; doi:10.1016/j.lanwpc.2026.101979)
Supplement: Supplementary file 1 [file mmc1.docx]

**Supplementary Materials for**

**Trends in *Helicobacter pylori* prevalence, force of infection, and attributable disease burden in China: a** **systematic review and Bayesian modelling study**

Dianqin Sun,^1^ Weiran Han,^1^ Fernando Alarid-Escudero,^2,3^ Jorge A. Roa-Contreras,^2^ Shaoming Wang,^4^ Duco T. Mülder,^1,8^ Robert J. Huang,^5^ Uri Ladabaum,^5^ Manon C. W. Spaander,^6^ M. Constanza Camargo,^7^ Jin Young Park,^8^ Wanqing Chen,^4^ Iris Lansdorp-Vogelaar,^1^ Jan A.C. Hontelez^1,9^

1 Department of Public Health, Erasmus MC, University Medical Center Rotterdam, Rotterdam, the Netherlands

2 Department of Health Policy, School of Medicine, Stanford University, Stanford, CA, United States

3 Center for Health Policy, Freeman Spogli Institute, Stanford University, CA, United States

4 National Cancer Center/National Clinical Research Center for Cancer/Cancer Hospital, Chinese Academy of Medical Sciences and Peking Union Medical College, Beijing, China

5 Division of Gastroenterology and Hepatology, Department of Medicine, Stanford University School of Medicine, Stanford, United States

6 Department of Gastroenterology and Hepatology, Erasmus MC, University Medical Center Rotterdam, the Netherlands

7 Division of Cancer Epidemiology and Genetics, National Cancer Institute, National Institutes of Health, Rockville, MD, United States

8 Early Detection, Prevention, and Infections Branch, International Agency for Research on Cancer, Lyon, France

9 Heidelberg Institute of Global Health (HIGH), Medical Faculty and University Hospital, Heidelberg University, Heidelberg, Germany

Table of Contents

[Supplementary Methods	4](#_Toc235185923)

[Literature search	4](#_Toc235185924)

[Table S1. Search strategy for systematic reviews or meta-analyses on *H. pylori* prevalence in China	4](#_Toc235185925)

[Table S2. Characteristics of selected meta-analyses on *H. pylori* prevalence in China	5](#_Toc235185926)

[Table S3. Search strategy for original studies on *H. pylori* prevalence in China	6](#_Toc235185927)

[Data extraction	7](#_Toc235185928)

[Extraction prompt for Gemini 2.5 Pro	8](#_Toc235185929)

[Method check prompt for Gemini 2.5 Pro	10](#_Toc235185930)

[Data cleaning and record selection based on age-band width	12](#_Toc235185931)

[Risk of bias checklist	13](#_Toc235185932)

[Table S4. Risk of bias checklist from Hoy et al., 2012^7^	13](#_Toc235185933)

[Rogan Gladen approach for test-accuracy adjustment	14](#_Toc235185934)

[Table S5. Sensitivity and specificity of different detection methods^a9^	14](#_Toc235185935)

[Structure of the Bayesian catalytic model	15](#_Toc235185936)

[Figure S1. Structure of the Bayesian catalytic model for *Helicobacter pylori* infection dynamics.	15](#_Toc235185937)

[Competing model structures	15](#_Toc235185938)

[Common model structures	17](#_Toc235185939)

[Bayesian estimation	19](#_Toc235185940)

[Likelihood	19](#_Toc235185941)

[Model fitting and comparison	19](#_Toc235185942)

[Relative risks of *Helicobacter pylori* infection	20](#_Toc235185943)

[Table S6. Relative risks of *Helicobacter pylori* infection used in the calculation of population attributable fraction^19,20^	20](#_Toc235185944)

[References	21](#_Toc235185945)

[Supplementary Results	23](#_Toc235185946)

[Tables	23](#_Toc235185947)

[Table S7. Characteristics of included studies used for meta-analysis and model training	23](#_Toc235185948)

[Table S8. Number of included studies and records by region and province in Mainland China	35](#_Toc235185949)

[Table S9. Risk of bias assessment for included studies used for meta-analysis and model training	36](#_Toc235185950)

[Table S10. Results of meta-analysis of corrected prevalence.	41](#_Toc235185951)

[Table S11. Results of meta-analysis of reported prevalence	43](#_Toc235185952)

[Table S12. Priors, results, and performance of four competing models	45](#_Toc235185953)

[Figures	46](#_Toc235185954)

[Figure S2. Age-period and age-cohort patterns of reported *Helicobacter pylori* prevalence in China, 1980-2023.	46](#_Toc235185955)

[Figure S3. Comparison between observed and model-predicted *Helicobacter pylori* A) seroprevalence, B) current-infection prevalence, and C) ever-infection prevalence.	48](#_Toc235185956)

[Figure S4. Comparison between observed and model-predicted *Helicobacter pylori* for the validation target^12^	49](#_Toc235185957)

[Figure S5. Posterior correlations among parameters in Model 4	50](#_Toc235185958)

[Figure S6. Estimates of *Helicobacter pylori* dynamics from the alternative Bayesian catalytic model	51](#_Toc235185959)

[Figure S7. Estimates of *Helicobacter pylori* associated disease burden from the alternative Bayesian catalytic model	52](#_Toc235185960)

[References	53](#_Toc235185961)

# Supplementary Methods

## Literature search

### Table S1. Search strategy for systematic reviews or meta-analyses on *H. pylori* prevalence in China

| **Databases** | **Search terms** | **Number of publications** |
| --- | --- | --- |
| Embase | ('helicobacter pylori'/exp OR 'helicobacter pylori':ti,ab,kw OR 'h pylori':ti,ab,kw) AND ('prevalence'/exp OR 'prevalence':ti,ab,kw) AND ('china'/exp OR 'china':ti,ab,kw OR 'chinese':ti,ab,kw OR 'asia':ti,ab,kw OR 'asian':ti,ab,kw OR 'global':ti,ab,kw OR 'world':ti,ab,kw OR 'worldwide':ti,ab,kw) AND ([systematic review]/lim OR [meta analysis]/lim OR 'scoping review') AND [2022-2025]/py | 54 |
| PubMed | ( "Helicobacter pylori"[Mesh] OR "Helicobacter pylori"[tiab] ) AND ( "Prevalence"[Mesh] OR prevalence[tiab] ) AND ( "China"[Mesh] OR China[tiab] OR "Asia"[Mesh] OR Asia[tiab] OR global[tiab] OR worldwide[tiab] ) AND ( systematic[sb] OR meta-analysis[pt] OR "scoping review"[tiab]) Filters: from 2020 - 2025 | 110 |

### Table S2. Characteristics of selected meta-analyses on *H. pylori* prevalence in China

| **Publication year** | | **First author** | | **Areas** | | **Whether including papers in Chinese** | | **Number of papers about China** | | **Inclusion and exclusion criteria regarding population representativeness and detection methods** | |  |
| --- | --- | --- | --- | --- | --- | --- | --- | --- | --- | --- | --- | --- |
|  | 2020 | | Mengmeng Li^1^ | | China | | Yes | | 98 | | - Including original studies reporting *H. pylori* prevalence in adults (aged 18 and above) from mainland China. - Excluding focusing exclusively on select groups (populations with certain socioeconomic/occupational statuses, the elderly, migrants, or patients with digestive diseases/symptoms). - Excluding studies with unspecified or inappropriate detection methods (e.g., using saliva tests) and those reporting prevalence only for children or not separating adult data. | |
|  | 2022 | | Shuai Ren ^2^ | | China | | Yes | | 412 | | - Including studies reflecting the general population through community surveys or hospital‐based physical examinations. - Including studies using one or more acceptable diagnostic tests (serology, stool antigen test, urea breath test, rapid urease test, histology, culture) | |
|  | 2022 | | Changzheng Yuan^3^ | | Worldwide | | No | | 17 | | - Including studies that reported the prevalence of *H pylori* infection in children aged 18 years or younger. - Excluding studies with samples that are purposely selected or are clearly unrepresentative of the general pediatric population (e.g., studies focusing on children with type 1 diabetes or obesity). | |
|  | 2023 | | Yunhao Li^4^ | | Worldwide | | No | | 37 | | - Excluding studies restricted to specific subgroups such as patients with cancer, pregnant individuals, or immunodeficient subjects. - Excluding studies that fail to state the prevalence of H. pylori or do not specify the diagnostic methods used. | |
|  | 2024 | | Yi-Chu Chen^5^ | | Worldwide | | No | | 162 | | - Including cross‑sectional studies or control groups from case‑control studies. - Excluding studies not representing the general population (e.g., family‑based studies, those from H. pylori eradication trials, patients with peptic ulcer disease). - Excluding studies reporting prevalence in non‑standard samples (saliva, gastric juice, or urine). | |
|  | 2024 | | Chelsea S. Taylor^6^ | | Worldwide | | No | | 10 | | - Including studies that reported the prevalence of non-migrant participants either designated asymptomatic or with no mention of gastrointestinal symptoms. - Including studies using serology. | |

### Table S3. Search strategy for original studies on *H. pylori* prevalence in China

| **Databases** | **Search terms** | **Number of publications** |
| --- | --- | --- |
| Embase | ('helicobacter pylori'/exp OR 'helicobacter pylori':ti,ab,kw OR 'h pylori':ti,ab,kw) AND ('prevalence'/exp OR 'prevalence':ti,ab,kw) AND ('china'/exp OR 'china':ti,ab,kw OR 'chinese':ti,ab,kw) AND [2022-2025]/py | 170 |
| PubMed | ( "Helicobacter pylori"[Mesh] OR "Helicobacter pylori"[tiab] ) AND ( "Prevalence"[Mesh] OR prevalence[tiab] ) AND ( "China"[Mesh] OR China[tiab] OR Chinese[tiab]) Filters: from 2022 - 2025 | 116 |
| CNKI | (SU='幽门螺旋杆菌' OR SU='幽门螺杆菌' OR SU='Hp' OR SU='Helicobacter pylori') AND (SU='感染率' OR SU='流行病学' OR SU='患病率' OR SU='检出率' OR SU='阳性率') Filters: from 2022 - 2025 | 439 |

## Data extraction

The extractor was instructed to capture study metadata, detection methods, age-specific prevalence, and subgroup-specific estimates. It generated standard flags to trigger manual review in the following situations: (1) missing N or P; (2) prevalence data reported only in figures; (3) non-standard or overlapping age-group definitions; (4) multiple prevalence series (e.g., different diagnostic tests or time points); (5) uncertain extraction; and (6) no eligible age-specific data. All flagged records were manually checked and corrected as needed.

We additionally conducted a second-pass extraction to verify three methodological items for each paper: (1) whether age-specific prevalence was reported only in supplementary materials; (2) whether prevalence estimates were adjusted for test accuracy; and (3) how prior H. pylori eradication therapy was handled. When these issues were identified, we applied the following rules. First, if age-specific prevalence data were available only in supplementary materials, we manually retrieved the age-specific numerators and denominators from those files. Second, if a study reported prevalence estimates corrected for test accuracy, we extracted the original unadjusted estimates. Third, if participants with a history of eradication therapy were included and classified as positive, we treated the reported measure as ever-infection prevalence.

We piloted the pipeline on 50 papers and iteratively refined the prompts until outputs were appropriate for structured extraction at scale. As part of this pilot, we randomly audited 10 English-language and 10 Chinese-language papers for field-level manual verification against the full texts. Satisfactory extraction was defined at the paper level as correct extraction of every prespecified item, including study characteristics, population description, diagnostic method, age group, number tested, and number positive. An error in any item constituted failure at the paper level. All 20 audited papers met this criterion, corresponding to 100% paper-level exact agreement.

During eligibility assessment, we identified two records in which the extracted population descriptions lacked detail and corrected them through direct review of the full texts. All data retained after full-text eligibility assessment were subsequently manually verified against the original articles. Final verification identified one study in which participants were classified as positive, negative, or indeterminate, but Gemini had extracted the denominator using only the positive and negative groups. We corrected the denominator to include all three groups. Manual review also identified three articles that analysed subsets of participants from larger included surveys. This overlap was not apparent from the population descriptions extracted by Gemini, the overlapping records were removed to avoid duplication.

### Extraction prompt for Gemini 2.5 Pro

***Objective:** Analyze the provided scientific manuscript PDF about Helicobacter pylori epidemiology to extract specific metadata, prevalence data, and cohort characteristics for a meta-analysis. Prioritize data from tables.*

***Manuscript Identifier:** {pdf_filename}*

***Extraction Tasks:***

*1. **Identify Metadata:***

**	`Journal_Name`, `First_Author`, `Study_Year`, `Survey_Year`, `Study_Location`, `Study_Population_Description`, `Diagnostic_Method`, `Language`: Extract as previously defined.*

**	`Overall_Age_Statistics`: Extract age statistics (Mean, SD, Range) for the **entire study population**.*

**	`Overall_Prevalence`: Extract the single, overall prevalence (`N`, `P`, `Percent`) for the **entire study population**.*

*2. **Extract Detailed Prevalence Data (by Subgroup):***

**	**Primary Target:** Search tables and text for prevalence data for specific subgroups (e.g., by sex, location, occupation).*

**	**Structure:** Report this data as a list of "data set" objects under the `Prevalence_Data` key.*

**	**CRITICAL RULE:** Create a "data set" object for a subgroup **ONLY IF** the manuscript provides **EITHER (A) age-stratified prevalence data OR (B) overall age statistics (mean, SD, or range) for that specific subgroup.***

**	**(A) CORRECT (Age-Stratified):** A table shows prevalence for Males broken down by age groups (20-29, 30-39, etc.). This is a valid subgroup.*

**	**(B) CORRECT (Overall Age Stats):** A table shows that for 'Urban' residents, the mean age was 42.5 years and overall prevalence was 45%. This is also a valid subgroup.*

**	**INCORRECT:** The text says "prevalence in rural residents was 38%" but gives **no age stratification and no age statistics (mean, sd, range)** for the rural group. **DO NOT** create a subgroup object for this. It lacks the required age context.*

**	**For each valid data set, extract the following UNIFIED structure:***

**	`Subgroup_Description`: A clear string describing the subgroup. (e.g., "Sex: Male", "Region: Urban").*

**	`Subgroup_Age_Statistics`: An object for the overall age stats of **this subgroup**. Use `{{"Mean": null, "SD": null, "Range": null}}` if not provided for the subgroup.*

**	`Data_Points`: A list of objects containing all prevalence data points for the subgroup.*

**	This list should contain one object for each **age-stratified row**.*

**	**NEW RULE:** If the paper also provides summary/total numbers (N, P, Percent) for the entire subgroup, add one final object to this list with the `Age_Group` field set to the special string `"Overall"`.*

**	For subgroups that *only* have overall numbers (Case B), this list will contain a single object where `Age_Group` is `"Overall"`.*

**	Each object in the list must contain:*

**	`Age_Group`: The age group (e.g., "0-4", ">=60") or the special value `"Overall"`.*

**	`Sample_Size_N`: Total subjects for that row.*

**	`Positive_Cases_P`: Positive subjects for that row.*

**	`Reported_Prevalence_Percent`: The reported percentage for that row.*

*3. **Handle Special Cases & Flagging:*

**	`FLAG_MISSING_N_OR_P`: If any row in `Data_Points` is missing N or P.*

**	`FLAG_NO_AGE_SPECIFIC_DATA`: Apply if no subgroups with age-stratification or age-statistics are found. If relevant data is referenced to supplementary material which was not available, describe it in `Notes`.*

**	`FLAG_DATA_IN_FIGURE`**: If you determine that the primary age-specific prevalence data is presented graphically in a figure and not detailed adequately in tables or text. Briefly describe the figure content in `Notes`.*

**	`FLAG_NON_STANDARD_AGE_GROUP`**: If age groups are vaguely defined (e.g., "children", "adults", "elderly" without ranges) or use non-standard/overlapping ranges. Extract the group name as reported.*

**	`FLAG_MULTIPLE_PREVALENCE_MEASURES`**: If the paper reports multiple distinct sets of prevalence data (e.g., based on different diagnostic tests giving different results, different time points, or significantly different sub-populations within the same age group). Extract the primary/most relevant set if clear, otherwise flag it and briefly describe the situation in `Notes`.*

**	`FLAG_UNCERTAIN_EXTRACTION`**: If you are uncertain about the accuracy or completeness of the extracted numerical data (N, P) for any reason (e.g., complex table format, conflicting numbers).*

*4. **Output Format:***

**	Produce a single, valid JSON object (RFC 8259). Pay strict attention to syntax, especially commas between list elements.*

**	**JSON Structure Example (Reflecting the new unified model):***

*```json*

*{{{{*

*"Paper_ID": "example_paper.pdf",*

*"Journal_Name": "Journal of Epidemiology",*

*"First_Author": "Jane Doe",*

*"Study_Year": 2022,*

*"Survey_Year": "2019-2021",*

*"Study_Location": "Nationwide",*

*"Study_Population_Description": "General adult population",*

*"Diagnostic_Method": ["UBT"],*

*"Language": "English",*

*"Overall_Age_Statistics": {{{{"Mean": 45.2, "SD": 11.3, "Range": "18-75"}}}},*

*"Overall_Prevalence": {{{{"Sample_Size_N": 2000, "Positive_Cases_P": 840, "Prevalence_Percent": 42.0}}}},*

*"Prevalence_Data": [*

*{{{{*

*"Subgroup_Description": "Sex: Male",*

*"Subgroup_Age_Statistics": {{{{"Mean": 46.1, "SD": 10.8, "Range": null}}}},*

*"Data_Points": [*

*{{{{*

*"Age_Group": "20-29",*

*"Sample_Size_N": 250,*

*"Positive_Cases_P": 80,*

*"Reported_Prevalence_Percent": 32.0*

*}}}},*

*{{{{*

*"Age_Group": "30-39",*

*"Sample_Size_N": 280,*

*"Positive_Cases_P": null,*

*"Reported_Prevalence_Percent": 41.1*

*}}}},*

*{{{{*

*"Age_Group": "Overall",*

*"Sample_Size_N": 1050,*

*"Positive_Cases_P": 472,*

*"Reported_Prevalence_Percent": 45.0*

*}}}}*

*]*

*}}}},*

*{{{{*

*"Subgroup_Description": "Region: Urban",*

*"Subgroup_Age_Statistics": {{{{"Mean": 42.5, "SD": 12.1, "Range": "18-70"}}}},*

*"Data_Points": [*

*{{{{*

*"Age_Group": "Overall",*

*"Sample_Size_N": 850,*

*"Positive_Cases_P": 382,*

*"Reported_Prevalence_Percent": 44.9*

*}}}}*

*]*

*}}}}*

*],*

*"Extraction_Flags": ["FLAG_MISSING_N_OR_P"],*

*"Notes": "P value for males age 30-39 was not provided in Table 2."*

*}}}}*

*```*

**	The output must ONLY be the JSON object.*

### Method check prompt for Gemini 2.5 Pro

***Objective:** Analyze the provided scientific manuscript PDF about *Helicobacter pylori* epidemiology to extract key bibliographic metadata and confirm three methodological items (Triad Checks). Focus on methods, results, tables, footnotes, and supplementary references.*

***Manuscript Identifier:** {pdf_filename}*

*---*

*### 1. Basic Study Metadata*

*Extract only:*

*- `Journal_Name`*

*- `First_Author`*

*- `Study_Year`*

*- `Language`*

*If any item is unclear or not explicitly stated, assign `"Unknown"`.*

*---*

*### 2. Triad Checks (Required)*

*For each item below, answer `"Yes"`, `"No"`, or `"Unknown"`, and include a **short evidence snippet or description** from the text.*

*#### A. Supplementary or Appendix Prevalence Data*

*- `Supplementary_Prevalence_Present`: Did the paper indicate that prevalence data (especially age-specific data) were reported in **supplementary materials or appendix**?*

*- `Supplementary_Details`: Brief description or citation (e.g., “Supplementary Table S2”, “Appendix A”, “Online-only data”).*

***Search cues:** “Supplementary”, “Appendix”, “Table S”, “Online-only”, “eTable”, “Supplementary Figure”.*

*---*

*#### B. Correction for Diagnostic Test Accuracy (Se/Sp)*

*- `SeSp_Correction_Applied`: Did authors adjust the reported prevalence for **test misclassification** (imperfect sensitivity/specificity)?*

*- `SeSp_Correction_Method`: Short phrase describing the method or statement (e.g., “Rogan–Gladen correction”, “Bayesian latent class model”).*

*If no correction mentioned, return `null`.*

***Search cues:** “sensitivity”, “specificity”, “misclassification”, “Rogan–Gladen”, “adjusted prevalence”, “test accuracy”.*

*---*

*#### C. Handling of Prior *H. pylori* Eradication Treatment*

*- `Prior_Treatment_Handled`: Did the study **exclude**, **adjust for**, **report**, or otherwise **address** participants with previous eradication therapy?*

*Include situations where **treated individuals were still counted as infected**.*

*- `Prior_Treatment_Strategy`: e.g., “Excluded previously eradicated”, “Included treated as infected”, “Adjusted in regression”, “Not handled”.*

*- `Prior_Treatment_Details`: Short evidence excerpt (e.g., “Participants who had received eradication therapy were included and classified as positive”).*

***Search cues:** “eradication therapy”, “previous treatment”, “prior eradication”, “history of *H. pylori* treatment”, “triple therapy”, “antibiotic therapy”, “PPI + clarithromycin”.*

*---*

*### 3. Flagging*

*If relevant, include:*

*- `FLAG_UNCERTAIN_EXTRACTION`: Extraction uncertain due to ambiguous or incomplete information.*

*---*

*### 4. Output Format*

*Return a **single valid JSON object** (RFC 8259). Do not include explanations outside JSON.*

*#### Example:*

*```json*

*{*

*"Paper_ID": "example_paper.pdf",*

*"Journal_Name": "Journal of Epidemiology",*

*"First_Author": "Jane Doe",*

*"Study_Year": 2021,*

*"Language": "English",*

*"Triad_Checks": {*

*"Supplementary_Prevalence_Present": "Yes",*

*"Supplementary_Details": "Supplementary Table S2 lists age-specific prevalence by sex.",*

*"SeSp_Correction_Applied": "No",*

*"SeSp_Correction_Method": null,*

*"Prior_Treatment_Handled": "Yes",*

*"Prior_Treatment_Strategy": "Included treated individuals as infected",*

*"Prior_Treatment_Details": "Participants who had received eradication therapy were counted as positive."*

*},*

*"Extraction_Flags": ["FLAG_UNCERTAIN_EXTRACTION"],*

*"Notes": "Supplementary tables were referenced but not contained in this PDF."*

*}*

**	The output must ONLY be the JSON object.*

## Data cleaning and record selection based on age-band width

**For age bounds,** if an explicit age range was reported, we used its lower and upper limits. If only the mean and standard deviation (SD) were reported, we approximated bounds as **mean ± 2.58×SD**. For one-sided bounds (e.g., “<10”, “≥60”), we first removed those records with upper limits > 10 or lower limit <60 and created the missing bound by **±5 years.** The **age point** used for analysis was chosen in this order: 1) reported mean if available; otherwise, 2) the midpoint of the age range; for one-sided bounds, 3) boundary **±2.5 years**. When the survey year was missing, we imputed it as “**study publication year-2**”. For prevalence calculations, to avoid zero-cell issues we applied a small continuity correction (**set number of positive cases=0 to 1**) when there is no positive cases.

Given that *H. pylori* prevalence changes rapidly at young ages and varies by birth cohort, we retained strata only when age-band width met prespecified limits**: ≤5 years when the age point was ≤10 years; ≤10 years for >10–30 years; ≤20 years for >30–60 years; and ≤30 years for >60 years.**

## Risk of bias checklist

### Table S4. Risk of bias checklist from Hoy et al., 2012^7^

| **Risk of bias item** | **Criteria for answers^a^** |
| --- | --- |
| ***External Validity*** |  |
| **1.** Was the study’s target population a close representation of the national population in relation to relevant variables, e.g. age, sex, occupation? | Score 0 (Low risk): The study was a national survey, OR a multi-site study explicitly covering multiple provinces or regions across China (e.g., covering both urban and rural areas, or eastern/central/western regions).  Score 1 (High risk): The study was conducted in a single province, single city, single center, or single institution, with no evidence of broader national coverage. One province or one city alone is NOT sufficient for Low risk. |
| **2.** Was the sampling frame a true or close representation of the target population? | Score 0 (Low risk): Probability-based, stratified, cluster, or systematic sampling frame covering the defined target population.  Score 1 (High risk): Sampling frame clearly limited to a subgroup of the target population (e.g., one neighbourhood, one institution). |
| **3.** Was some form of random selection used to select the sample, OR, was a census undertaken? | Score 0 (Low risk): Some form of random selection was used OR everyone in the target population was sampled.  Score 1 (High risk): No random selection was used AND NOT everyone in the target population was sampled. |
| **4.** Was the likelihood of non-response bias minimal? | Score 0 (Low risk): Response/participation rate >=75%, OR a formal analysis showed no significant difference between participants and non-participants.  Score 1 (High risk): Response rate <75% AND no analysis of non-participant characteristics reported. If no response rate is reported at all, score 1. |
| ***Internal Validity*** |  |
| **5.** Were data collected directly from the subjects (as opposed to a proxy)? | Under our inclusion criteria, all included studies met the internal-validity items and were therefore assessed as having low risk of bias for this domain. |
| **6.** Was an acceptable case definition used in the study? |  |
| **7.** Was the study instrument that measured the parameter of interest shown to have reliability and validity (if necessary)? |  |
| **8.** Was the same mode of data collection used for all subjects? |  |
| **9.** Was the length of the shortest prevalence period for the parameter of interest appropriate? |  |
| **10.** Were the numerator(s) and denominator(s) for the parameter of interest appropriate? |  |

a: the criteria was adapted based on our study aim

## Rogan Gladen approach for test-accuracy adjustment

For each study record, we calculated the observed prevalence using the Rogan-Gladen formula^8^:

$${prevalence}_{adjusted}=\frac{observed prevalence+specificity-1}{sensitivity+specificity-1}$$

Test-specific values of sensitivity and specificity were obtained from the summary in a Chinese consensus report and listed in **Table S5**.^9^ After adjustment, we estimated the uncertainty of each adjusted prevalence using the Wilson score method as implemented in the epiR package. Records with reported prevalence lower than (1 – specificity), for which the adjustment yields invalid estimates, were excluded from the meta-analysis.

### Table S5. Sensitivity and specificity of different detection methods^a9^

| **Measured prevalence type** | **Detection methods** | **Sensitivity (%)** | **Specificity (%)** |
| --- | --- | --- | --- |
| Current-infection prevalence | Urea breath test (UBT, including C^14^ UBT and C^13^ UBT) | 94.8 | 95.0 |
|  | Stool antigen test | 84.5 | 93.4 |
|  | String test with qPCR^18^ | 77.2 | 93.1 |
|  | Rapid urease test | 92.0 | 93.2 |
|  | Histology | 89.3 | 97.7 |
|  | Culture^9^ | 77.9 | 100.0 |
|  | Histology; Urea breath test (1/2) | 99.4 | 92.8 |
|  | Histology; Rapid urease test (2/2) | 82.2 | 99.8 |
|  | Stool antigen test; Urea breath test (2/2) | 80.1 | 99.7 |
|  | Serology; Urea breath test (2/2) | 77.1 | 99.2 |
|  | Serology; Stool antigen test; Urea breath test (2/3) | 95.6 | 97.9 |
|  | Serology; Stool antigen test; Urea breath test (3/3) | 65.1 | 99.9 |
| Seroprevalence | Serology (including immunoblot, ELISA, and Immunofluorescence assays) | 81.3 | 84.2 |
|  | Serology; Urea breath test (1/2) | 99.0 | 80.0 |
|  | Serology; Stool antigen test; Urea breath test (1/3) | 99.8 | 74.7 |
|  | Serology; Rapid urease test; Urea breath test (1/3) | 99.9 | 74.6 |

a: all the combinations were calculated assuming test independence

ELISA: enzyme-linked immunosorbent assay; PCR: polymerase chain reaction

## ****Structure of the Bayesian catalytic model****

### Figure S1. ****Structure of the Bayesian catalytic model for**** Helicobacter pylori ****infection dynamics.****

Individuals begin uninfected (U) and acquire infection at an age-and-cohort-specific force of infection. The cohort-specific asymptote was parameterized using either piecewise-linear (B1) or I-spline (B2) functions. The model includes two alternative structures for progression from infection to treatment (dashed box). In structure A1, all infected individuals clear at a period-dependent rate, independent of infection duration. In structure A2, infected individuals first progress through an infection-time-dependent process before becoming eligible for treatment at the symptomatic state (S), which then clears at a period-dependent rate. After clearance, individuals remain seropositive (C) before seroreversion (N) at constant rate. Current infection corresponds to the infected and symptomatic states, seropositivity to infected, symptomatic, and cleared-but-seropositive states, and ever infection to all except the uninfected state.

### Competing model structures

**A. Rate from infection to treatment**

*A1. Infection-time-independent rate (exponential dwell)*

In this specification, the transition from current infection to treatment was dependent only on calendar year $\rho(t)$ and independent of infection time. Let *a* denote age and *c* the birth cohort (so calendar time $t=c+a$). The underlying ordinary differential equations (ODEs) were:

$$\frac{d}{da}U\left( a,c \right)=-\lambda\left( a,c \right)U\left( a,c \right),$$

$$\frac{d}{da}I\left( a,c \right)=\lambda\left( a,c \right)U\left( a,c \right)-\rho\left( t \right)I(a,c),$$

$$\frac{d}{da}C\left( a,c \right)=\rho\left( t \right)I\left( a,c \right)-\omega C(a,c),$$

$$\frac{d}{da}N\left( a,c \right)=\omega C(a,c),$$

$$U\left( 0,c \right)=1, I\left( 0,c \right)=C\left( 0,c \right)=N\left( 0,c \right)=0$$

where $U\left( a,c \right)$, $I\left( a,c \right)$, $C\left( a,c \right)$, $N\left( a,c \right)$ are the probabilities of being uninfected, infected, cleared but still seropositive, and seroreversed, respectively. This structure implies an exponential distribution of dwell times from infection to clearance.

*A2. Infection-time-dependent rate (Erlang dwell)*

Most *H. pylori* infections are asymptomatic, and very few asymptomatic individuals in China seek for test-and-treatment of H. pylori. Only about 15-20% of infected cases eventually develop dyspepsia or ulcer.^10,11^ We therefore split new infections into a persistent component (80%) and a progression component (20%). For those who progress, we assumed a rising hazard since infection. We modeled the underlying dwelling time with an Erlang distribution of *J=6* phases and rate $\theta$=J/mean=6/35 per year. We set the mean time from infection to symptoms to 35 years to place most symptom onset in mid-adulthood, consistent with childhood acquisition of *H.pylori* and the observed peak of peptic ulcer incidence at ages 40-60.^12,13^ The erlang distribution was equivalent to the sum of 6 exponential ($\theta$) waiting times. This preserves our Markov structure, enabling stable, closed-form segment updates while matching the clinically plausible rise in hazard over time.

The underlying ODEs are:

$$\frac{d}{da}U\left( a,c \right)=-\lambda\left( a,c \right)U\left( a,c \right),$$

$$\frac{d}{da}I_{persistent}\left( a,c \right)=-p_{persistent}\lambda\left( a,c \right)U\left( a,c \right),$$

$$\frac{d}{da}I_{1}\left( a,c \right)=p_{symptom}\lambda\left( a,c \right)U\left( a,c \right)-\theta I_{1}\left( a,c \right),$$

$$\frac{d}{da}I_{j}\left( a,c \right)=\theta I_{j-1}\left( a,c \right)-\theta I_{j} \left( j=2,\ldots.,6 \right),$$

$$\frac{d}{da}S\left( a,c \right)=\theta I_{6}\left( a,c \right)-\rho\left( t \right)S\left( a,c \right)$$

$$\frac{d}{da}C\left( a,c \right)=\rho\left( t \right)S\left( a,c \right)-\omega C\left( a,c \right),$$

$$\frac{d}{da}N\left( a,c \right)=\omega C\left( a,c \right),$$

$$U\left( 0,c \right)=1, I\left( 0,c \right)=S\left( 0,c \right)=C\left( 0,c \right)=N\left( 0,c \right)=0$$

This structure simulated an **infection-time-dependent hazard** from infection to treatment eligibility (symptoms), while period treatment $\rho(t)$ still controls the clearance rate after the chain completion (S). In computation, we evolved these states by clearance segments (described below). We used closed-form updates for existing mass and 5-point Gauss-Legendre quadrature for new inflows within each segment. Gauss-Legendre can achieve very small errors on smooth integrands such as products of exponentials and low-degree polynomials just like our case.

**B. Cohort-specific asymptote** $\boldsymbol{\alpha}_{\boldsymbol{c}}$

*B1. piecewise-linear*

We modeled logit $\alpha_{c}$ as a monotone piecewise-linear with change-points in 1950 and 1980.

$$logit(\alpha_{c})=\left\{ \begin{aligned} \beta_{0}-m_{1}\left( c-1950 \right)/10, &c\leq1950, \\ \beta_{0}-m_{2}\left( c-1950 \right)/10, &1950<c\leq1980, \\ \beta_{0}-m_{2}\left( 1980-1950 \right)/10-m_{3}(c-1980)/10, &c>1980, \end{aligned} \right.$$

where $\beta_{0}$ is the intercept, $m_{1},m_{2},m_{3}\geq0$, This specification captures well-known cohort declines. The knot 1950 marks the early years of the newly established People’s Republic of China, initiating infrastructural changes. The knot 1980 aligns with China’s “Reform and Opening up” policies implemented through the early 1980s, which transformed living conditions, urbanization, and crowding patterns.

To ensure non-negative slopes and sample unconstrained reals, we did the below transformation:

$$m_{k}=\log\left( {1+e}^{m_{k}^{raw}} \right) (k=1,2,3)$$

*B2. I-spline*

However, the piecewise-linear might lead to abrupt change and not be flexible enough, so we tested another assumption that the $\alpha_{c}$ followed a **monotone decreasing** using an I-spline basis

$$\alpha_{c}=\alpha_{hi}-(\alpha_{hi}-\alpha_{lo})\sum_{k=1}^{K} w_{k}I_{k}(c)$$

where $I_{k}(c)$ is a monotone I-spline basis scaled to $[0,1]$, the weights $w_{k}$ are non-negative and sum to one, and $0<\alpha_{lo}<\alpha_{hi}<1$. This ensures $\alpha_{c}$ decreases smoothly for later birth cohorts. We set terciles of the birth years as two internal knots and used degree=2 (quadratic I-spline).

### Common model structures

**Period-varying treatment hazards**

Since 1990s, H. pylori has been established as a causal pathogen and standardized eradication regimens then entered guidelines and routine practice. To capture the rapid scale-up of eradication therapy after the early 1990s, the annual treatment hazard is set to zero before 1990 and increased thereafter following a logistic curve evaluated at 5-year blocks:

$$\rho(t)=\frac{\rho_{max}}{1+e^{-\frac{t-t_{50}}{5}}}$$

where $\rho_{\max}$ is the maximum annual hazard, $t_{50}$ is the mid-point year of expansion. To have stable sampling and easily place priors, we did the probability and hazard transformation:

$$\rho_{max}=-\log\left( 1-f_{max} \right)$$

$f_{max}$ is the maximum probability per year and parameterized as below, which ensures a range between 0 and 1.

$$f_{max}=\frac{e^{\rho_{logit\_fmax}}}{1+e^{\rho_{logit\_fmax}}}$$

**Seroreversion**

Following successful *H. pylori* eradication, treated individuals were assumed to remain seropositive before eventually seroconverting. We modeled the time from cure to seroreversion using an exponential distribution with rate $\omega$, such that the probability of remaining seropositive $t$years after cure is $\exp(-\omega t)$. We assigned a weakly informative prior, $omega\sim\mathrm{Lognormal}\left( \log\left( 0.48 \right),\text{ }0.4 \right)$(**Table S11**). This prior places the median of omega at 0.48 per year, corresponding to an average seropositive duration of approximately 2.1 years, while allowing substantial uncertainty. It was chosen to be broadly consistent with empirical evidence that roughly 50% of cured patients remain seropositive at 12 months, 15–45% at 24 months^14–16^.

**Diagnostic misclassification**

For each observation $i$, current-infection tests are positive with probability

$$p_{i}=\text{Se}_{i}\text{ }P_{I}+(1-\text{Sp}_{i})(1-P_{I})$$

where $P_{I}$ is the modelled true current-infection prevalence, $\text{Se}_{i}$ and $\text{Sp}_{i}$correspond to test sensitivity and specificity. Serology tests detect both current infection and cleared-but-seropositive states using the same misclassification logic. For ever-infection observations, we considered the misclassification due to imperfect tests and recall bias (Se=0.90, Sp=0.98) to reflect that individuals may be counted as ever infected if either the current test or recall indicates eradication history.

**Study-level random effects**

We used two parameters to control the study-level random effects.

$$z_{study}\mathcal{\sim N}\left( 0,1 \right)$$

$$u_{study}{=\tau z}_{study}$$

where each study gets its $z_{study}$, indicating the study-level deviations. $\tau$ is a single scalar>0 and measures between-study heterogeneity level. This two-parameter setting was more stable than simply sampling a study-specific deviation from a general normal distribution.

## Bayesian estimation

### Likelihood

For study $i$, let $y_{i}$be the observed number of positives among $n_{i}$tested individuals. We assumed

$$\theta_{i}\sim\mathrm{Beta}(\phi p_{i},\phi(1-p_{i})),y_{i}\mid\theta_{i}\sim\mathrm{Binomial}(n_{i},\theta_{i}),$$

where $p_{i}$is the modelled mean positive proportion and $\phi$is a precision parameter. Marginally, this implies

$$y_{i}\sim\mathrm{BetaBinomial}(n_{i},\phi p_{i},\phi(1-p_{i})).$$

Integrating over $\theta_{i}$yields the Beta-Binomial likelihood

$$\Pr(y_{i}\mid n_{i},p_{i},\phi)=\binom{n_{i}}{y_{i}}\frac{B\text{ }\left( y_{i}+\phi p_{i},\text{ }n_{i}-y_{i}+\phi(1-p_{i}) \right)}{B\text{ }\left( \phi p_{i},\text{ }\phi(1-p_{i}) \right)}.$$

where $B(\cdot,\cdot)$denotes the beta function, and the full likelihood was the product over observations. This parameterization centers the likelihood at the model-predicted proportion $p_{i}$while allowing extra-binomial variation through $\phi$. Weakly informative priors were specified as shown in **Table S11**.

### Model fitting and comparison

Each model was fitted using the No-U-Turn sampler with four Markov chains (5000 iterations each, 5000 warm-up, average acceptance probability = 0.99, maximum tree depth= 12) to ensure high acceptance and avoid divergences. Convergence was confirmed by effective sample sizes, rank-normalized statistics (R-hat < 1.01), and absence of divergent transitions. Model performance was evaluated using Pareto-smoothed importance sampling leave-one-out cross-validation (PSIS-LOO), which approximates leave-one-out cross-validation by re-weighting posterior draws.^17^ This method avoids the computing burden of refitting models while still generating an estimate of out-of-sample predictive accuracy. For each model, we reported the expected log predictive density (ELPD), its standard error, and the effective number of parameters. ELPD is the sum of the expected log-likelihood across all observations, with higher values indicating better predictive performance. The effective number of parameters is calculated by summing, across observations, the differences between their predictive fit when included in model fitting and when treated as unseen data. Larger values indicate that the model adapts more closely to individual observations and therefore has greater effective flexibility.

## Relative risks of *Helicobacter pylori* infection

### Table S6. Relative risks of *Helicobacter pylori* infection used in the calculation of population attributable fraction^19,20^

| **Disease** | **Subtype** | **Relative risks (95% confidence intervals)^a^** |
| --- | --- | --- |
| Gastric cancer^20^ | Cardia | 3.06 (1.54, 6.10) |
|  | Non-cardia | 5.94 (3.25, 10.86) |
| Peptic ulcer disease^19^ | Gastric ulcer | 1.11 (0.84, 1.47) |
|  | Duodenal ulcer | 2.71 (1.79,4.08) |

a: We used reported hazard ratios to approximate relative risks here.

## References

1.	Li M, Sun Y, Yang J, et al. Time trends and other sources of variation in helicobacter pylori infection in mainland China: A systematic review and meta-analysis. *Helicobacter*. 2020;25(5):e12729. doi:10.1111/hel.12729

2.	Ren S, Cai P, Liu Y, et al. Prevalence of *Helicobacter pylori* infection in China: A systematic review and meta‐analysis. *J Gastroenterol Hepatol*. 2022;37(3):464-470. doi:10.1111/jgh.15751

3.	Yuan C, Adeloye D, Luk TT, et al. The global prevalence of and factors associated with helicobacter pylori infection in children: A systematic review and meta-analysis. *Lancet Child Adolesc Health*. 2022;6(3):185-194. doi:10.1016/S2352-4642(21)00400-4

4.	Li Y, Choi H, Leung K, Jiang F, Graham DY, Leung WK. Global prevalence of helicobacter pylori infection between 1980 and 2022: A systematic review and meta-analysis. *Lancet Gastroenterol Hepatol*. 2023;8(6):553-564. doi:10.1016/S2468-1253(23)00070-5

5.	Chen YC, Malfertheiner P, Yu HT, et al. Global prevalence of helicobacter pylori infection and incidence of gastric cancer between 1980 and 2022. *Gastroenterology*. 2024;166(4):605-619. doi:10.1053/j.gastro.2023.12.022

6.	Taylor CS, McMahon MV, Ward ZJ, et al. Birth cohort and age-specific trends in global helicobacter pylori seroprevalence: A scoping review. *Lancet Reg Health – Am*. 2025;41. doi:10.1016/j.lana.2024.100877

7.	Hoy D, Brooks P, Woolf A, et al. Assessing risk of bias in prevalence studies: modification of an existing tool and evidence of interrater agreement. *J Clin Epidemiol*. 2012;65(9):934-939. doi:10.1016/j.jclinepi.2011.11.014

8.	ROGAN WJ, GLADEN B. Estimating prevalence from the results of a screening test. *Am J Epidemiol*. 1978;107(1):71-76. doi:10.1093/oxfordjournals.aje.a112510

9.	Ding SZ, Du YQ, Lu H, et al. Chinese Consensus Report on Family-Based Helicobacter pylori Infection Control and Management (2021 Edition). *Gut*. 2022;71(2):238-253. doi:10.1136/gutjnl-2021-325630

10.	Malfertheiner P, Camargo MC, El-Omar E, et al. Helicobacter pylori infection. *Nat Rev Dis Primer*. 2023;9(1):1-24. doi:10.1038/s41572-023-00431-8

11.	Dore MP, Pes GM, Bassotti G, Usai-Satta P. Dyspepsia: When and how to test for helicobacter pylori infection. *Gastroenterol Res Pract*. 2016;2016:8463614. doi:10.1155/2016/8463614

12.	Yang H, Zhang M, Li H, et al. Prevalence of common upper gastrointestinal diseases in Chinese adults aged 18-64 years. *Sci Bull*. 2024;69(24):3889-3898. doi:10.1016/j.scib.2024.07.048

13.	Xie X, Ren K, Zhou Z, Dang C, Zhang H. The global, regional and national burden of peptic ulcer disease from 1990 to 2019: A population-based study. *BMC Gastroenterol*. 2022;22(1):58. doi:10.1186/s12876-022-02130-2

14.	Miernyk KM, Bruden DL, Bruce MG, et al. Dynamics of helicobacter pylori-specific immunoglobulin G for 2 years after successful eradication of helicobacter pylori infection in an American Indian and alaska native population. *Clin Vaccine Immunol*. 2007;14(1):85-86. doi:10.1128/CVI.00253-06

15.	Kato S, Furuyama N, Ozawa K, Ohnuma K, Iinuma K. Long-term follow-up study of serum immunoglobulin G and immunoglobulin a antibodies after helicobacter pyloriEradication. *Pediatrics*. 1999;104(2):e22. doi:10.1542/peds.104.2.e22

16.	Lee JH, Kim N, Chung JI, et al. Long-term follow up of helicobacter pylori IgG serology after eradication and reinfection rate of H. pylori in south korea. *Helicobacter*. 2008;13(4):288-294. doi:10.1111/j.1523-5378.2008.00616.x

17.	Vehtari A, Gelman A, Gabry J. Practical Bayesian model evaluation using leave-one-out cross-validation and WAIC. *Stat Comput*. 2017;27(5):1413-1432. doi:10.1007/s11222-016-9696-4

18.	Si YT, Xiong XS, Zhang SK, et al. Detection of Helicobacter pylori Infection and Antibiotic Resistance via String Test Coupled With qPCR Method: A Comparative Methodological Study. *J Gastroenterol Hepatol*. 2025;40(7):1763-1771. doi:10.1111/jgh.16962

19.	Kartsonaki C, Yao P, Butt J, et al. Infectious pathogens and risk of esophageal, gastric and duodenal cancers and ulcers in China: A case-cohort study. *Int J Cancer*. 2024;154(8):1423-1432. doi:10.1002/ijc.34814

20.	Yang L, Kartsonaki C, Yao P, et al. The relative and attributable risks of cardia and non-cardia gastric cancer associated with helicobacter pylori infection in China: A case-cohort study. *Lancet Public Health*. 2021;6(12):e888-e896. doi:10.1016/s2468-2667(21)00164-x

# Supplementary Results

## Tables

### Table S7. Characteristics of included studies used for meta-analysis and model training

| **First author** | **Publication year** | **Survey year** | **Detecting methods** | **Subgroup** | **Observed data (age groups: positive cases/sample size)** |
| --- | --- | --- | --- | --- | --- |
| Forman^1^ | 1990 | 1983 | Serology (antibody) |  | 60~64: 175/280; 55~59: 205/318; 50~54: 214/319; 45~49: 180/312; 40~44: 150/271; 35~39: 213/382 |
| Chen^2^ | 1993 |  | Serology (antibody) |  | 7~8: 37/68 |
| Zhang^3^ | 1995 | 1992.12-1993.2 | Serology (antibody) |  | 20~29: 42/66 |
| Liu^4^ | 1996 |  | Serology (antibody) | Healthy controls (exclude medical stuff) | >70: 30/92; 60~69: 53/112; 50~59: 52/104; 40~49: 69/146; 30~39: 61/132; 20~29: 48/116 |
| Ma^5^ | 1998 | 1994 | Serology (antibody) |  | 55~64: 48/77; 45~54: 51/81; 35~44: 96/131 |
| Zhang^6^ | 1998 |  | Serology (antibody) | Location: High-incidence area (Linqu County) | 50~59: 47/60; 40~49: 80/110 |
|  |  |  |  | Location: Low-incidence area (Cangshan County) | 50~59: 32/57; 40~49: 43/70 |
| Tang^7^ | 1999 | 1998.11 | Serology (antibody) |  | 80~89: 34/60 |
| Wong^8^ | 1999 | 1993-1994 | Serology (antibody) | Residents in Changle | 56~65: 179/216; 46~55: 387/494; 36~45: 605/746 |
| Xu^9^ | 1999 |  | Serology (antibody) |  | 9: 65/167; 8: 51/146; 7: 51/165; 13~14: 25/81; 12: 79/167; 11: 110/226; 10: 77/167 |
| Zhang^10^ | 1999 | 1996-1997 | Serology (antibody) |  | 61~70: 108/179; 51~60: 140/239; 41~50: 291/425; 30~40: 428/615 |
| Chen^11^ | 2000 | 1998-1999 | Serology (antibody) |  | 3~6: 36/1014; 7~14: 102/408; 18~22: 94/264 |
| Liu^12^ | 2000 | 1998-09-1999-05 | Serology (antibody) |  | 50~80: 140/200; 4~6: 20/100; 30~50: 116/200 |
| Brown^13^ | 2001 | 1997-1998 | Serology (antibody) |  | 55~59: 170/299; 45~54: 537/893; 40~44: 530/848; 61~69: 273/478; 35~39: 484/770 |
| Zhou^14^ | 2001 |  | Serology (antibody) |  | 9: 32/77; 8: 40/77; 7: 30/77; 12: 70/127; 11: 70/134; 10: 45/84 |
| Chen^15^ | 2002 | 1990 | Serology (antibody) | Jiading | 70~85: 37/88; 60~69: 77/116; 50~59: 83/121; 40~49: 110/168; 30~39: 117/182; 20~29: 86/147; 10~19: 71/124 |
| Niu^16^ | 2003 | 2001 | Serology (antibody) |  | 6~7: 21/51; 5: 71/185; 4: 61/183; 2~3: 19/61 |
| Chen^17^ | 2003 | 1990 and 2001 | Serology (antibody) | Survey Year: 1990 (Urban Shanghai) | 70~87: 30/92; 60~69: 53/112; 50~59: 52/104; 40~49: 53/112; 30~39: 61/132; 20~29: 48/116; 10~19: 47/110 |
|  |  |  |  | Survey Year: 2001 (Urban Shanghai) | 70~87: 67/95; 60~69: 93/147; 50~59: 110/151; 40~49: 322/460; 30~39: 152/251; 20~29: 88/168; 10~19: 21/53 |
| Wang^18^ | 2003 | 2002 | Serology (antibody) |  | 9~12: 72/235; 5~8: 52/210; 13~14: 92/246; 1~4: 32/216; 1: 8/93 |
| Xue^19^ | 2003 |  | Serology (antibody) |  | 9: 36/78; 8: 31/77; 7: 26/75; 11: 68/130; 10: 43/85 |
| Zeng^20^ | 2003 |  | Serology (antibody) | Region: Guangdong (Low prevalence), Group: Controls | 21.2 (SD 1.35): 67/192 |
|  |  |  |  | Region: Shanxi (High prevalence), Group: Controls | 21.8 (SD 1.95): 83/169 |
| Zhang^21^ | 2003 | 1999-2000 | Serology (antibody) | Xi'an Suburban Area - Hp infection (HpU-IgG) | 13~14: 32/57; 11: 28/55; 10: 41/72 |
|  |  |  |  | Xi'an Urban Area - Hp infection (HpU-IgG) | 9: 36/67; 8: 39/69; 7: 33/57; 3: 20/57; 12: 31/53; 11: 41/61; 10: 43/65 |
| Li^22^ | 2004 | 2000-2001 | Serology (antibody) |  | 3~6: 98/448 |
| Mei^23^ | 2004 |  | Stool antigen test; Urea breath test |  | 9: 22/104; 8: 19/102; 7: 21/108; 6: 12/95; 5: 11/97; 4: 7/85; 3: 12/79; 12: 26/86; 11: 43/175; 10: 33/143 |
| Wang^24^ | 2004 | 2001-2002 | Serology (antibody) | Ethnic group: Mulam (Guangxi Luocheng, China) | 7~14: 44/76 |
|  |  |  |  | Ethnic group: Zhuang (Guangxi Luocheng, China) | 7~14: 35/54 |
| Wang^25^ | 2004 | 2001.11-2002.10 | Serology (antibody) |  | 50~71: 57/89; 40~49: 53/85; 30~39: 71/126; 20~29: 41/76; 18~19: 43/81; 13~17: 33/68 |
| Zhuang^26^ | 2004 | 2002-2004 | Serology (antibody) |  | 3~6: 253/817; <=3: 17/57 |
| Cao^27^ | 2005 |  | Serology (antibody); Urea breath test | Location: Lanzhou | 60~70: 67/113; 50~59: 47/76; 40~49: 61/108; 30~39: 41/73; 20~29: 39/67 |
|  |  |  |  | Location: Tianzhu | 60~73: 102/137; 50~59: 195/257; 40~49: 247/330; 30~39: 380/475; 20~29: 108/144 |
| Chen^28^ | 2005 | 2003-2004 | Serology (antibody); Stool antigen test; Urea breath test |  | 55~59: 44/57; 50~54: 98/133; 45~49: 178/264; 40~44: 163/249; 35~39: 129/217; 30~34: 208/317; 25~29: 242/347; 20~24: 98/146; 15~19: 28/63 |
| Gu ^29^ | 2005 | 2002- 2004 | Serology (antibody) |  | 9~12: 50/130; 5~8: 36/110; 3~4: 13/60 |
| Liu^30^ | 2006 | 2000- 2001 | Biopsy/Histology; Rapid urease test2 |  | 56(± 2): 16/97 |
| Xiang^31^ | 2006 |  | Biopsy/Histology |  | 40~49: 108/291; 30~39: 130/412; 20~29: 76/289; 15~19: 20/105; 50~75: 142/395 |
| Chen^32^ | 2007 | 2003 | Serology (antibody) |  | 50~59: 94/163; 5~9: 24/105; 40~49: 129/204; 30~39: 107/196; 20~29: 135/253; 10~19: 68/185; 1~4: 35/180 |
| Lu^33^ | 2007 | 2005 | Serology (antibody) |  | 80~87: 114/186; 70~79: 316/462; 60~69: 350/524 |
| Nie^34^ | 2007 | 2002 | Urea breath test |  | 45~54: 62/101; 55~64: 30/64; 35~44: 38/77 |
| Xu ^35^ | 2007 |  | Serology (antibody) |  | 18~23: 95/276 |
| Shi^36^ | 2008 | 2004-2005 | Serology (antibody); Urea breath test |  | 60~69: 107/180; 50~59: 239/372; 40~49: 171/297; 30~39: 193/287; 20~29: 47/84; 70~100: 64/102 |
| Cheng^37^ | 2009 | 2003 | Urea breath test |  | 61~70: 50/94; 51~60: 164/369; 41~50: 181/397; 31~40: 110/222; 11~20: 24/52 |
| Guo ^38^ | 2009 | 2006 | Serology (antibody) |  | 50~60: 805/1279; 40~50: 767/1259; 30~40: 789/1274; 20~30: 812/1310 |
| He ^39^ | 2009 | 2007-2009 | Serology (antibody) |  | 50~59: 98/182; 40~49: 94/186; 30~39: 110/256; 20~29: 96/240; 15~19: 30/105; 60~81: 102/195 |
| Li^40^ | 2009 | 2004 | Serology (antibody); Urea breath test |  | 9: 336/1729; 8: 212/1097; 7: 157/904; 6: 73/486; 12~15: 165/698; 11: 395/1619; 10: 307/1628 |
| Wang ^41^ | 2009 | 2007-2008 | Serology (antibody) |  | 51~60: 822/1284; 41~50: 980/1840; 31~40: 676/1556 |
| Wu ^42^ | 2009 | 2005-2007 | Serology (antibody); Urea breath test |  | 90~95: 246/426 |
| Xi^43^ | 2009 |  | Serology (antibody) |  | 9: 158/805; 8: 155/854; 7: 232/1410; 12: 316/1296; 11: 115/518; 10: 293/1418; 6: 70/481 |
| Yang^44^ | 2009 | 2006 | Serology (antibody) | Sex: Female | 40~59: 95/159; ≥60: 67/113 |
|  |  |  |  | Sex: Male | 40~59: 97/163; ≥60: 92/157 |
| Zeng ^45^ | 2009 | 2008 | Serology (antibody) |  | 50~59: 248/519; 40~49: 310/757; 30~39: 357/1097; 20~29: 147/518 |
| Zhang ^46^ | 2009 | 2006 | Stool antigen test | Adults in Muping County (2006) | 60~79: 55/104; 50~59: 111/208; 40~49: 102/214 |
|  |  |  |  | Adults in Yanqing County (2006) | 60~79: 47/110; 50~59: 95/215; 40~49: 66/178 |
|  |  |  |  | Children in Muping County (2006) | 8~9: 32/122; 14~15: 55/131; 12~13: 59/142; 10~11: 50/125 |
|  |  |  |  | Children in Yanqing County (2006) | 8~9: 20/130; 14~15: 37/125; 12~13: 37/125; 10~11: 38/136 |
| Zhang^47^ | 2009 | 2007- 2008 | Stool antigen test |  | 6~9: 169/240; 3~5: 68/99; 15~18: 120/159; 10~14: 321/440 |
| Zhang ^48^ | 2009 |  | Serology (antibody) |  | 50~59: 60/96; 40~49: 123/168; 30~39: 151/199; 20~29: 58/89; 10~19: 53/104; 60~86: 41/62 |
| Zhang^49^ | 2009 |  | Serology (antibody) |  | 71~80: 56/103; 61~70: 255/445; 51~60: 274/453; 41~50: 184/330; 31~40: 161/282; 21~30: 107/225; 11~20: 19/54 |
| Fang^50^ | 2010 | 2008-03 to 2009-07 | Urea breath test |  | 60~69: 42/71; 50~59: 75/136; 40~49: 249/447; 30~39: 656/1251; 20~29: 740/1556; 10~19: 139/342 |
| Peng^51^ | 2010 | 2003 | Serology (antibody) |  | 9~10: 84/374; 7~8: 46/280; 5~6: 28/166; 3~4: 65/376; 13~15: 68/200; 11~12: 84/441; 0~2: 10/53 |
| Tong^52^ | 2010 | 2007-06 to 2010-06 | Urea breath test | Sex: Female | 31~50: 193/433 |
|  |  |  |  | Sex: Male | 31~50: 230/502 |
| Wang^53^ | 2010 | 2006-07 - 2009-07 | Urea breath test |  | 27~42: 138/213 |
| Wu ^54^ | 2010 | 2007-04 - 2008-10 | Urea breath test | Sex: Female | 50~59: 149/323; 40~49: 231/395; 30~39: 146/234; 20~29: 69/98; 60~86: 31/83 |
|  |  |  |  | Sex: Male | 50~59: 452/598; 40~49: 490/726; 30~39: 279/442; 20~29: 133/227; 60~86: 97/118 |
| Chen^55^ | 2011 | 2009 | Serology (antibody) |  | 50~59: 771/1646; 40~49: 1369/2682; 30~39: 1801/4049; 20~29: 849/2169; 60~81: 524/1147; 10~19: 98/253 |
| Chen ^56^ | 2011 | 2010-12 to 2011-10 | Urea breath test |  | 50~59: 50/108; 40~49: 101/198; 30~39: 87/194; 20~29: 62/156; 60~88: 39/87; 10~19: 23/59 |
| Deng ^57^ | 2011 | 2008-2010 | Serology (antibody) | Sex: Female, Year: 2008 | 50~59: 126/304; 40~49: 182/490; 30~39: 246/844; 20~29: 122/440; 60~82: 56/136 |
|  |  |  |  | Sex: Female, Year: 2009 | 50~59: 192/487; 40~49: 252/751; 30~39: 335/1148; 20~29: 119/701; 60~82: 87/243 |
|  |  |  |  | Sex: Female, Year: 2010 | 50~59: 209/574; 40~49: 318/1065; 30~39: 403/1266; 20~29: 380/1110; 60~82: 122/365 |
|  |  |  |  | Sex: Male, Year: 2008 | 50~59: 370/734; 40~49: 438/1024; 30~39: 472/1350; 20~29: 168/596; 60~82: 232/590 |
|  |  |  |  | Sex: Male, Year: 2009 | 50~59: 398/849; 40~49: 583/1583; 30~39: 533/1674; 20~29: 257/843; 60~82: 240/650 |
|  |  |  |  | Sex: Male, Year: 2010 | 50~59: 373/1125; 40~49: 728/2149; 30~39: 722/2109; 20~29: 388/1174; 60~82: 246/776 |
| Zou^58^ | 2011 | 2007 - 2008 | Serology (antibody) |  | 70~80: 47/62; 60~69: 99/148; 50~59: 182/251; 40~49: 254/357; 30~39: 111/149 |
| Qian ^59^ | 2011 | 2010.6-2010.12 | Serology (antibody) |  | 41~50: 501/1064; 31~40: 455/990; 51~60: 204/384 |
| Ran ^60^ | 2011 | 2009 | Serology (antibody) |  | 60~65: 70/220; 50~59: 140/335; 40~49: 194/620; 30~39: 162/632; 21~29: 85/381 |
| Chen ^61^ | 2012 | 2009 - 2010 | Serology (antibody) |  | 50~59: 101/191; 40~49: 297/641; 30~39: 148/359; 20~29: 33/212 |
| Hua^62^ | 2012 | 2009-08 to 2011-05 | Biopsy/Histology; Urea breath test |  | 60~69: 79/215; 50~59: 934/1593; 40~49: 1084/1712; 30~39: 586/691 |
| Huang^63^ | 2012 | 2008-12 to 2010-06 | Urea breath test |  | 60~69: 412/792; 50~59: 972/1714; 40~49: 1698/3091; 30~39: 1016/2030; 20~29: 184/412 |
| Li^64^ | 2012 | 2010-06-01 to 2011-06-01 | Serology (antibody) |  | 60~69: 17/167; 50~59: 79/465; 40~49: 119/827; 30~39: 83/508; 20~29: 47/210; >=70: 22/106 |
| Liu ^65^ | 2012 | 2009-2011 | Serology (antibody) |  | 60~78: 299/524 |
| Liu ^66^ | 2012 | 2011-05-2012-04 | Serology (antibody) |  | 50~59: 809/1971; 40~49: 1882/4350; 30~39: 1620/3968; 20~29: 788/2264; 60~78: 425/1079 |
| Sun ^67^ | 2012 | 2011.04-2011.07 | Urea breath test |  | 71~80: 318/475; 60~70: 364/555; 80~86: 76/120 |
| Wan ^68^ | 2012 | 2008-2010 | Urea breath test | Year of survey: 2008 | 40~60: 3679/6913 |
|  |  |  |  | Year of survey: 2009 | 40~60: 3236/6913 |
|  |  |  |  | Year of survey: 2010 | 40~60: 3236/6913 |
| Wang^69^ | 2012 | 2011- 2012 | Urea breath test |  | 60~69: 88/182; 50~59: 279/536; 40~49: 350/766; 30~39: 183/464; 20~29: 89/212 |
| Yan^70^ | 2012 | Feb 2010 - Jun 2011 | Urea breath test |  | 70~79: 12/53; 60~69: 21/106; 50~59: 229/503; 40~49: 747/952; 30~39: 434/573; 20~29: 240/670 |
| Zhao^71^ | 2012 | 2010.01-2010.07 | Urea breath test |  | 51~55: 37/62; 46~50: 44/92; 41~45: 62/136; 36~40: 54/120 |
| Zheng^72^ | 2012 |  | Serology (antibody) | City: Changle | 50~70: 27/58; 40~49: 47/90; 30~39: 37/76 |
|  |  |  |  | City: Fuan | 50~70: 22/68; 40~49: 30/98; 30~39: 15/63 |
| Zhou^73^ | 2012 | 2011 | Urea breath test |  | 60~85: 338/549; 40~59: 2774/4096 |
| Zhu^74^ | 2012 | 2010-2011 | Serology (antibody) |  | 31~40: 803/2211; 21~30: 492/1637; 71~80: 246/680 |
| Guo^75^ | 2013 | 2012-10 – 2013-03 | Serology (antibody); Urea breath test | Region: North Shaanxi | 30~39: 51/79; 40~49: 68/115; 50~59: 60/106; 60~69: 31/61 |
|  |  |  |  | Region: South Shaanxi | 50~59: 42/96; 60~69: 23/70 |
| Huang ^76^ | 2013 | 2011 | Serology (antibody) |  | 15~24: 227/461 |
| Mai ^77^ | 2013 | 2009-2012 | Serology (antibody) |  | 6~9: 79/231; 10~12: 119/253 |
| Wang^78^ | 2013 | 2012 | Urea breath test |  | 65~70: 250/441; 60~64: 245/421; 55~59: 230/434; 50~54: 190/334; 45~49: 210/365; 40~44: 199/362; 35~39: 119/213 |
| Yang ^79^ | 2013 | 2011 | Urea breath test |  | 50~59: 90/172; 40~49: 159/345; 22~39: 192/497 |
| Zhao^80^ | 2013 | 2010-06 to 2012-06 | Urea breath test |  | 7~10: 107/206; 4~6: 48/103; 11~14: 93/151 |
| Shen^81^ | 2013 | 2012 | Urea breath test |  | 36~50: 248/553 |
| Cui ^82^ | 2014 | 2007-09-2011-08 | Urea breath test |  | 50~59: 163/350; 70~85: 34/89; 60~69: 80/221 |
| Duan ^83^ | 2014 | 2013 | Urea breath test |  | 50~59: 1416/2572; 40~49: 1209/2474; 30~39: 733/1942; 20~29: 446/1851; 13~19: 376/1785; 60~87: 929/1899 |
| Liang ^84^ | 2014 | 2012 | Urea breath test |  | 31~50: 1700/2911; 20~30: 357/669; 51~74: 337/690; 16~19: 46/96 |
| Zhang^85^ | 2014 | 2010 | Serology (antibody) |  | 75~79: 322/384; 70~74: 408/476; 65~69: 389/497; 60~64: 343/395; >= 80: 211/254 |
| Tang ^86^ | 2014 | 2009-2012 | Urea breath test |  | 60~69: 412/792; 50~59: 972/1714; 40~49: 1698/3091; 30~39: 1016/2030; 20~29: 184/412; 70~95: 158/315 |
| Wang ^87^ | 2014 | 2009-10/2010-12 | Stool antigen test |  | 5: 8/82; 4: 6/89; 3: 2/89; 2: 1/112; 1: 1/81; 0: 5/188 |
| Wang ^88^ | 2014 | 2013 | Serology (antibody) |  | 61~70: 26/83; 51~60: 20/71; 41~50: 19/85; 31~40: 37/173; 21~30: 59/252; 11~20: 13/55; 71~80: 22/73 |
| Wei ^89^ | 2014 | 2012-2013 | Serology (antibody) |  | 61~70: 124/150; 51~60: 204/300; 41~50: 158/250; 31~40: 114/200 |
| Zhu^90^ | 2014 | 2009 - 2011 | Urea breath test |  | 60~69: 285/500; 50~59: 1374/2295; 40~49: 1508/2326; 30~39: 267/294 |
| Zhang ^91^ | 2014 | 2012 | Serology (antibody) |  | 6~7: 34/140; 5: 49/324; 4: 46/457; 3: 16/229 |
| Ding^92^ | 2015 | 2009-2011 | Stool antigen test |  | 0: 2/330; 7~9: 32/528; 4~6: 45/624; 16~18: 55/408; 13~15: 55/685; 10~12: 34/308; 1~3: 6/289; 0~1: 8/319 |
| Kan^93^ | 2015 | 2013 | Serology (antibody) |  | 17~20: 3/104 |
| Jiang ^94^ | 2015 | 2012-12_2013-11 | Serology (antibody) |  | 71~80: 42/127; 61~70: 147/438; 51~60: 309/912; 41~50: 420/1320; 31~40: 255/928; 21~30: 73/370 |
| Li ^95^ | 2015 | 2013-08 to 2014-12 | Serology (antibody) |  | 60~69: 110/415; 50~59: 273/1056; 40~49: 721/2372; 30~39: 537/2054; 20~29: 187/887; >=70: 65/231 |
| Xu^96^ | 2015 | 1986-1989 | Serology (antibody) |  | 45~64: 447/541 |
| Wang ^97^ | 2015 | 2013-01-01 to 2014-08-31 | Urea breath test |  | 40~59: 1448/3457 |
| Zhang ^98^ | 2015 | 2009-2013 | Serology (antibody) |  | 60~70: 555/1808; 50~59: 1051/3311; 40~49: 1367/4890; 30~39: 1051/3959; 20~29: 529/2479; 18~19: 11/59 |
| Chen ^99^ | 2016 | 2012-01_2015-10 | Urea breath test |  | 40~59: 2177/4830; 60~78: 719/1760; 14~19: 64/188 |
| Huang ^100^ | 2016 | 2014 | Serology (antibody); Urea breath test |  | 7~8: 24/99; 9~10: 82/216; 8~9: 40/174; 17~18: 72/160; 16~17: 183/377; 15~16: 125/314; 14~15: 28/87; 13~14: 27/86; 12~13: 38/104; 11~12: 31/131; 10~11: 46/193 |
| Ji ^101^ | 2016 | 2013-09 to 2015-09 | Serology (antibody) |  | 60~74: 102/391; 45~59: 357/1441 |
| Jia ^102^ | 2016 | 2008- 2012 | Serology (antibody) | control | 44.0~55.0: 478/961 |
| Li ^103^ | 2016 | 2013 | Serology (antibody) | Sex: Female | 50~60: 55/146; 40~49: 135/345; 30~39: 48/147; 20~29: 57/200 |
|  |  |  |  | Sex: Male | 50~60: 152/366; 40~49: 221/450; 30~39: 96/251; 20~29: 69/242 |
| Li ^104^ | 2016 | 2012-01-01 to 2015-01-31 | Urea breath test |  | 60~69: 337/715; 50~59: 1684/3771; 40~49: 1647/4348; 30~39: 264/811; 20~29: 16/79; 70~82: 40/128 |
| Pan ^105^ | 2016 | 2011-2013 | Urea breath test |  | 50~54: 22640/41685; 45~49: 25146/44081; 40~44: 22650/38485; 35~39: 15388/25882; 30~34: 8578/14399; 25~29: 11571/19438 |
| Tian ^106^ | 2016 | 2013-2014 | Urea breath test |  | >70: 82/88; 50~70: 77/106; 35~50: 111/178 |
| Wang ^107^ | 2016 | 2013-2014 | Urea breath test |  | 51~60: 443/843; 41~50: 896/1817; 31~40: 496/1103; 61~87: 200/387 |
| Wen ^108^ | 2016 | 2016 | Urea breath test | Sex: Female | 70~93: 12/89; 50~69: 122/370; 30~49: 198/622 |
|  |  |  |  | Sex: Male | 70~93: 33/88; 50~69: 408/1095; 30~49: 484/1168 |
| Yu ^109^ | 2016 | 2014-05 – 2015-07 | Urea breath test |  | 61~70: 90/179; 51~60: 527/997; 41~50: 708/1315; 31~40: 175/344; 71~96: 138/280 |
| Ayinuer ^110^ | 2017 | 2014-06 to 2015-05 | Urea breath test | Ethnicity: Han | 21~30: 96/296; 31~40: 219/578; 41~50: 221/496; 51~60: 162/350; 61~77: 33/98 |
|  |  |  |  | Ethnicity: Uyghur | 31~40: 61/85; 41~50: 75/112; 51~60: 30/52 |
| Cao ^111^ | 2017 | 2012-01 to 2015-12 | Serology (antibody) |  | 7~17: 61/311; 60~65: 62/114; 50~59: 260/470; 40~49: 474/868; 30~39: 337/642 |
| Li ^112^ | 2017 |  | Urea breath test |  | 61~70: 35/80; 51~60: 260/500; 41~50: 320/620; 31~40: 92/177; 81~94: 30/74 |
| Liu^113^ | 2017 | 2014 | Urea breath test |  | 35~44: 1089/3325; 25~34: 605/2037; 18~24: 75/288 |
| Lu ^114^ | 2017 | 2013 -2015 | Serology (antibody); Stool antigen test; Urea breath test3 |  | 7~10: 39/97; 4~6: 25/85; 1~3: 10/69 |
| Xu^115^ | 2017 | 2012-2016 | Serology (antibody) |  | 45~60: 1352/2847; >60: 2027/4338 |
| Qu ^116^ | 2017 | 2014-01-01 to 2015-12-31 | Urea breath test |  | 60~69: 1048/2724; 50~59: 2008/4896; 40~49: 3239/8108; 30~39: 1949/5249; 20~29: 784/2613; >=70: 532/1570 |
| Shan ^117^ | 2017 | 2010-2011 | Serology (antibody) |  | 65~74: 36/76; 55~64: 57/117; 45~54: 39/84; 35~44: 21/58; 75~87: 33/60 |
| Yu^118^ | 2017 | April 2013-June 2016 | Serology (antibody) |  | 18~24: 526/2497; 25~34: 3417/12991; 35~44: 3687/11798; 45~54: 3894/10880; 55~64: 3279/8744; 65~95: 1579/4389 |
| Zhang ^119^ | 2017 | 2015-07 – 2016-06 | Urea breath test |  | 60~85: 25/65; 40~59: 238/490 |
| Zhao^120^ | 2017 | 2009-2010 | Stool antigen test |  | 9~11: 17/151; 6~8: 17/198; 3~5: 8/155; 15~18: 49/375; 12~14: 34/256; 1~2: 2/51 |
| Hu^121^ | 2018 | July 2015 to December 2016 | Serology (antibody) |  | 50~59: 29/63; 40~49: 26/55 |
| Gao ^122^ | 2018 | 2016-10_2017-10 | Urea breath test |  | 76~85: 79/160; 66~75: 138/266; 56~65: 164/302; 46~55: 85/148; 35~45: 95/124 |
| Xu^123^ | 2018 | 2012-2015 | Serology (antibody) |  | 45~60: 1406/2993; >= 60: 2063/4453 |
| Meng ^124^ | 2018 | 2017 | Serology (antibody) |  | 46~60: 2682/9482; 30~45: 2730/11518; 60~85: 796/2538 |
| Su^125^ | 2018 | 2015-2016 | Urea breath test |  | 50~59: 1885/4101; 40~49: 2160/4473; >=60: 1267/3015 |
| Sun ^126^ | 2018 | 2016-09-01_2016-12-31 | Serology (antibody) |  | 70~93: 171/711; 60~69: 271/989; 50~59: 324/1140; 40~49: 146/418 |
| Wei ^127^ | 2018 | 2016 | Urea breath test |  | 50~59: 446/855; 40~49: 564/920; 30~39: 274/434 |
| Yu ^128^ | 2018 | 2014-05-05 to 2015-07-28 | Urea breath test | Sex: Female | 61~70: 60/125; 51~60: 107/195; 41~50: 198/387; 31~40: 70/138; 71~96: 62/131 |
|  |  |  |  | Sex: Male | 61~70: 71/148; 51~60: 420/802; 41~50: 510/928; 31~40: 105/206; 71~96: 77/152 |
| Zhou^129^ | 2018 | 2014 | Urea breath test |  | 9: 48/107; 8: 22/84; 10: 30/102 |
| Hou^130^ | 2019 | 2015 | Urea breath test |  | 75~84: 59/162; 65~74: 85/239; 85~100: 84/245 |
| Hu ^131^ | 2019 | 2016 - 2017 | Urea breath test |  | 45~59: 1460/3895; 60~86: 431/1181 |
| Shu^132^ | 2019 | 2015-2016 | Urea breath test | Sex: Female | 45~59: 339/1392 |
|  |  |  |  | Sex: Male | 45~59: 490/1622 |
| Zhang ^133^ | 2019 | 2017-2018 | Urea breath test |  | 31~40: 511/1434; 41~50: 443/1165; 51~60: 408/1071; 61~70: 194/549; 70~90: 51/167 |
| Gao^134^ | 2020 | January 2013 to September 2017 | Serology (antibody) |  | 0.5~3: 2113/6896 |
| Zhu^135^ | 2020 | 2017 | Urea breath test |  | >=80: 97/168; 75~79: 83/171; 70~74: 95/209; 65~69: 120/263; 60~64: 112/279 |
| Hu^136^ | 2020 | 2018 | Urea breath test |  | 9: 47/260; 8: 48/207; 7: 38/231; 6: 17/160; 12: 12/72; 11: 36/202; 10: 28/223 |
| Zhang^137^ | 2020 | 2017-2019 | Urea breath test |  | 71~80: 599/1247; 61~70: 1979/4103; 51~60: 4531/9710; 41~50: 5139/11123; 31~40: 3486/7884 |
| Wang^138^ | 2020 | 2018 | Urea breath test | Sex: Female | 52.23 ± 1.865: 22/62 |
|  |  |  |  | Sex: Male | 54.74 ± 3.441: 60/167 |
| Liu^139^ | 2021 | 2019 | Urea breath test |  | 60~69: 32/114; 50~59: 49/151; 40~49: 52/180; 70~87: 21/59 |
| Shen^140^ | 2021 | June 2018 to December 2019 | Urea breath test |  | 60~69: 27/120; >=70: 12/62 |
| Wang^141^ | 2021 | 2016-2017 | Stool antigen test |  | 0~3: 57/400 |
| Li^142^ | 2022 | 2020-2021 | Urea breath test |  | 20~29: 294/930; 30~39: 650/1942; 40~49: 444/1200; 50~59: 276/756; >=60: 88/179 |
| Feng ^143^ | 2022 | 2020 | Urea breath test |  | 45~59: 4369/11050 |
| Zhou^144^ | 2022 | 2014 | Urea breath test |  | 50~60: 67/358; >=60: 112/606 |
| Luo ^145^ | 2022 | 2021-08-2022-05 | Urea breath test |  | 30~40: 154/338; 40~50: 172/343; 50~60: 174/353; >=60: 49/105 |
| Shi ^146^ | 2022 | 2020 | Urea breath test |  | 41~60: 774/1360; >60: 243/491 |
| Wang ^147^ | 2022 | 2019-06-2020-06 | Urea breath test |  | 45~58: 452/990; 59~80: 328/646 |
| Xue ^148^ | 2022 | 2020-05-2020-10 | Urea breath test |  | 30~39: 312/1176; 40~49: 342/1385; 50~59: 275/1128; 60~65: 40/245 |
| Wu^149^ | 2022 | 2020 - 2021 | Urea breath test | Sex: Female | 50~59: 125/345; 40~49: 165/442; 30~39: 236/719; >=60: 52/97 |
|  |  |  |  | Sex: Male | 50~59: 151/411; 40~49: 279/758; 30~39: 414/1223; >=60: 39/93 |
| Zhou^150^ | 2022 | 2017 and 2022 | Urea breath test | 2017 | 44~59: 1315/2169 |
|  |  |  |  | 2022 | 44~59: 683/1577 |
| Chang ^151^ | 2023 | 2022 | Urea breath test |  | 40~59: 138/335; 60~74: 254/557 |
| Chen ^152^ | 2023 | 2020-2022 | Urea breath test |  | 30~49: 148/346; 50~69: 312/517; >=70: 17/64 |
| Chen ^153^ | 2023 | August 2022 to April 2023 | Urea breath test |  | 14~18: 12/58; 45~64: 622/1508; 65~93: 251/579 |
| Liao^154^ | 2023 | 2015-2016 | Serology (antibody) |  | 40~49: 249/743; 50~59: 233/677; 60~69: 182/542 |
| Jiang ^155^ | 2023 | 2022 | Urea breath test |  | 41~60: 191/383; 61~85: 49/136 |
| Zou^156^ | 2023 | 2009-2010, 2013-2014, 2019-2021 | Urea breath test | 2009-2010 | 20~29: 184/412; 30~39: 1016/2030; 40~49: 1698/3091; 50~59: 972/1714; 60~69: 412/792; >=70: 158/315 |
|  |  |  |  | 2013-2014 | 20~29: 637/1570; 30~39: 1213/3021; 40~49: 2074/4863; 50~59: 1583/3715; 60~69: 937/2329; >=70: 413/1027 |
|  |  |  |  | 2019-2021 | 20~29: 374/1463; 30~39: 1131/3843; 40~49: 1824/5836; 50~59: 1543/4773; 60~69: 618/1954; >=70: 93/327 |
| Lan ^157^ | 2023 | 2018-2022 | Serology (antibody) |  | 31~40: 4018/8602; 41~49: 2720/5824; 50~60: 2874/5780; 61~70: 1084/2285; 71~95: 371/933 |
| Liu ^158^ | 2023 | 2021-08-2023-05 | Urea breath test | Ethnicity: Han | 41~60: 16/55 |
|  |  |  |  | Ethnicity: Tibetan | 41~60: 29/64; >60: 31/62 |
| Tong ^159^ | 2023 |  | Urea breath test |  | 45~65: 91/277; >65: 27/97 |
| Wang ^160^ | 2023 | 2022 | Urea breath test |  | 45~59: 1489/4628; >=60: 645/2140 |
| Wang ^161^ | 2023 | 2020 | Serology (antibody) |  | 40~49: 25/104; 50~59: 45/191; 60~69: 17/98 |
| Wang ^162^ | 2023 | 2020-2021 | Urea breath test |  | >65: 1251/2287 |
| Xiao ^163^ | 2023 | 2021 | Urea breath test |  | 30~39: 2286/6086; 40~49: 1197/3105; 50~59: 1121/2804 |
| Liu^164^ | 2023 | 2014-2018 | Urea breath test | Sex: Female | 30~39: 4162/11220; 40~49: 5314/13008; 50~59: 3533/8642; 60~69: 1514/3892; >=70: 566/1619 |
|  |  |  |  | Sex: Male | 30~39: 5453/14050; 40~49: 7205/17306; 50~59: 5227/11664; 60~69: 2144/5126; >=70: 1023/2558 |
| Zhao ^165^ | 2023 | 2019 | Urea breath test |  | 20~29: 322/656; 30~39: 1164/2235; 40~49: 1883/3664; 50~59: 875/1560 |
| Zhou ^166^ | 2023 | 2021 | Urea breath test |  | 1-5: 122/703; 6-10: 293/1468; 11-15: 287/1277; 16-20: 245/860; 21-25: 362/1161; 26-30: 751/1871; 31-35: 1373/3230; 36-40: 1277/2991; 41-45: 1058/2431; 46-50: 1257/2636; 51-55: 1284/2820; 56-60: 1433/3120; 61-65: 1141/2520; 66-70: 1025/2281; 71-75: 431/1052; >=76: 286/703 |
| He ^167^ | 2024 | 2022-07--2023-04-30 | Serology (antibody) |  | 45~59: 403/1191; 60~72: 208/480 |
| Wang^168^ | 2024 | 2023 | PCR/string test |  | 18~24: 194/915; 25~29: 282/1047; 30~34: 411/1441; 35~39: 502/1682; 40~44: 465/1578; 45~49: 494/1758; 50~54: 495/1990; 55~60: 651/2491 |
| Tang ^169^ | 2024 | 2021-03 to 2023-03 | Urea breath test |  | 30~40: 35/135; 40~49: 56/182; 50~59: 87/219 |
| Wang ^170^ | 2024 | 2020 | Urea breath test |  | 30~40: 362/987; 40~49: 794/1655; 50~59: 1445/3055; 60~69: 1288/3025 |
| Wang ^171^ | 2024 | 2019-2021 | Urea breath test | Ethnicity: Han | 21~40: 2798/7797 |
|  |  |  |  | Ethnicity: Uyghur | 21~40: 3048/4318 |
| Wu ^172^ | 2024 | 2020-2022 | Urea breath test |  | 30~40: 50/118; 40~49: 102/252; 50~59: 59/173; 60~69: 44/133; 70~79: 22/56 |
| Yan^173^ | 2024 | 2015-2017 | Urea breath test |  | 40~49: 4067/8763; 50~59: 4741/10249; 60~69: 3636/8073 |
| Jiang ^174^ | 2025 | 2022-2023 | Serology (antibody) |  | 30~39: 450/615; 40~49: 610/855; 50~59: 844/1179; 60~90: 836/1234 |
| Li ^175^ | 2025 | 2023 | Urea breath test |  | 40~59: 497/1352; 60~79: 133/345 |
| Liu ^176^ | 2025 | 2022 | Urea breath test |  | 14: 52/153; 15: 58/168; 16: 51/159; 17: 57/166 |
| Sun ^177^ | 2025 | 2020-04-2023-04 | Urea breath test |  | 30~39: 37/154; 40~49: 62/209; 50~59: 98/256 |
| Tan ^178^ | 2025 | 2022-06-2023-06 | Serology (antibody) |  | 16~18: 73/280; 18~20: 206/691 |
| Wu ^179^ | 2025 | 2021 | Urea breath test |  | 41~60: 182/467 |
| Yan^180^ | 2025 | 2020-2023 |  | (14C-UBT) | 40~60: 1332/7934; 60~75: 308/1684 |
|  |  |  |  | (Serology) | 20~30: 486/1258; 30~40: 1184/2666; 40~50: 1780/3767; 50~60: 3258/6829; 60~75: 3111/6859 |
| Zhang ^181^ | 2025 | 2023 | Stool antigen test |  | ≤10: 64/670; 11~12: 133/398 |

### Table S8. Number of included studies and records by region and province in Mainland China

| **Region** | **Province** | **Study Count** | **Record Count** |
| --- | --- | --- | --- |
| **North** | Beijing | 16 | 72 |
|  | Tianjin | 2 | 24 |
|  | Hebei | 9 | 52 |
|  | Shanxi | 4 | 35 |
|  | Inner Mongolia | 5 | 37 |
| **Northeast** | Liaoning | 8 | 52 |
|  | Jilin | 6 | 37 |
|  | Heilongjiang | 4 | 33 |
| **East** | Shanghai | 17 | 107 |
|  | Jiangsu | 16 | 88 |
|  | Zhejiang | 11 | 63 |
|  | Anhui | 12 | 59 |
|  | Fujian | 7 | 46 |
|  | Jiangxi | 4 | 33 |
|  | Shandong | 15 | 82 |
| **Central** | Henan | 8 | 40 |
|  | Hubei | 8 | 48 |
|  | Hunan | 10 | 57 |
| **South** | Guangdong | 17 | 95 |
|  | Guangxi | 9 | 51 |
|  | Hainan | 6 | 34 |
| **Southwest** | Chongqing | 8 | 72 |
|  | Sichuan | 19 | 126 |
|  | Guizhou | 5 | 37 |
|  | Yunnan | 5 | 36 |
|  | Tibet | 1 | 16 |
| **Northwest** | Shaanxi | 11 | 66 |
|  | Gansu | 11 | 72 |
|  | Qinghai | 2 | 22 |
|  | Ningxia | 5 | 33 |
|  | Xinjiang | 9 | 59 |

Note: Studies and records covering multiple provinces were counted once in each relevant province; therefore, counts are not mutually exclusive and their sums may exceed the total numbers of included studies and records.

### Table S9. Risk of bias assessment for included studies used for meta-analysis and model training

| **First author** | **Publication year** | **Survey year** | **Detecting methods** | **Item1** | **Item2** | **Item3** | **Item4** |
| --- | --- | --- | --- | --- | --- | --- | --- |
| Forman^1^ | 1990 | 1983 | Serology (antibody) | 0 | 0 | 0 | 0 |
| Chen^2^ | 1993 |  | Serology (antibody) | 1 | 1 | 1 | 1 |
| Zhang^3^ | 1995 | 1992.12-1993.2 | Serology (antibody) | 0 | 1 | 1 | 1 |
| Liu^4^ | 1996 |  | Serology (antibody) | 1 | 1 | 1 | 1 |
| Ma^5^ | 1998 | 1994 | Serology (antibody) | 1 | 0 | 0 | 0 |
| Zhang^6^ | 1998 |  | Serology (antibody) | 1 | 1 | 0 | 1 |
| Tang^7^ | 1999 | 1998.11 | Serology (antibody) | 1 | 1 | 0 | 1 |
| Wong^8^ | 1999 | 1993-1994 | Serology (antibody) | 1 | 1 | 1 | 1 |
| Xu^9^ | 1999 |  | Serology (antibody) | 1 | 1 | 1 | 1 |
| Zhang^10^ | 1999 | 1996-1997 | Serology (antibody) | 1 | 0 | 0 | 1 |
| Chen^11^ | 2000 | 1998-1999 | Serology (antibody) | 1 | 1 | 0 | 1 |
| Liu^12^ | 2000 | 1998-09-1999-05 | Serology (antibody) | 1 | 1 | 0 | 1 |
| Brown^13^ | 2001 | 1997-1998 | Serology (antibody) | 1 | 0 | 0 | 0 |
| Zhou^14^ | 2001 |  | Serology (antibody) | 1 | 1 | 0 | 1 |
| Chen^15^ | 2002 | 1990 | Serology (antibody) | 1 | 1 | 1 | 1 |
| Niu^16^ | 2003 | 2001 | Serology (antibody) | 1 | 1 | 0 | 1 |
| Chen^17^ | 2003 | 1990 and 2001 | Serology (antibody) | 1 | 1 | 1 | 1 |
| Wang^18^ | 2003 | 2002 | Serology (antibody) | 1 | 1 | 1 | 1 |
| Xue^19^ | 2003 |  | Serology (antibody) | 1 | 1 | 0 | 1 |
| Zeng^20^ | 2003 |  | Serology (antibody) | 1 | 1 | 1 | 1 |
| Zhang^21^ | 2003 | 1999-2000 | Serology (antibody) | 1 | 0 | 0 | 1 |
| Li^22^ | 2004 | 2000-2001 | Serology (antibody) | 1 | 1 | 1 | 1 |
| Mei^23^ | 2004 |  | Stool antigen test; Urea breath test | 1 | 1 | 1 | 1 |
| Wang^24^ | 2004 | 2001-2002 | Serology (antibody) | 1 | 1 | 1 | 1 |
| Wang^25^ | 2004 | 2001.11-2002.10 | Serology (antibody) | 1 | 0 | 0 | 1 |
| Zhuang^26^ | 2004 | 2002-2004 | Serology (antibody) | 1 | 1 | 1 | 1 |
| Cao^27^ | 2005 |  | Serology (antibody); Urea breath test | 1 | 1 | 0 | 1 |
| Chen^28^ | 2005 | 2003-2004 | Serology (antibody); Stool antigen test; Urea breath test | 1 | 1 | 0 | 0 |
| Gu ^29^ | 2005 | 2002- 2004 | Serology (antibody) | 1 | 1 | 1 | 1 |
| Liu^30^ | 2006 | 2000- 2001 | Biopsy/Histology; Rapid urease test2 | 1 | 1 | 1 | 1 |
| Xiang^31^ | 2006 |  | Biopsy/Histology | 1 | 0 | 0 | 1 |
| Chen^32^ | 2007 | 2003 | Serology (antibody) | 1 | 1 | 0 | 1 |
| Lu^33^ | 2007 | 2005 | Serology (antibody) | 1 | 0 | 0 | 1 |
| Nie^34^ | 2007 | 2002 | Urea breath test | 1 | 1 | 0 | 1 |
| Xu ^35^ | 2007 |  | Serology (antibody) | 1 | 1 | 1 | 1 |
| Shi^36^ | 2008 | 2004-2005 | Serology (antibody); Urea breath test | 1 | 0 | 0 | 1 |
| Cheng^37^ | 2009 | 2003 | Urea breath test | 1 | 1 | 1 | 1 |
| Guo ^38^ | 2009 | 2006 | Serology (antibody) | 1 | 0 | 0 | 0 |
| He ^39^ | 2009 | 2007-2009 | Serology (antibody) | 1 | 1 | 0 | 1 |
| Li^40^ | 2009 | 2004 | Serology (antibody); Urea breath test | 1 | 0 | 0 | 1 |
| Wang ^41^ | 2009 | 2007-2008 | Serology (antibody) | 1 | 1 | 1 | 1 |
| Wu ^42^ | 2009 | 2005-2007 | Serology (antibody); Urea breath test | 1 | 0 | 0 | 1 |
| Xi^43^ | 2009 |  | Serology (antibody) | 1 | 1 | 1 | 1 |
| Yang^44^ | 2009 | 2006 | Serology (antibody) | 1 | 0 | 0 | 1 |
| Zeng ^45^ | 2009 | 2008 | Serology (antibody) | 1 | 1 | 1 | 1 |
| Zhang ^46^ | 2009 | 2006 | Stool antigen test | 1 | 1 | 0 | 1 |
| Zhang^47^ | 2009 | 2007- 2008 | Stool antigen test | 1 | 0 | 0 | 1 |
| Zhang ^48^ | 2009 |  | Serology (antibody) | 1 | 1 | 0 | 1 |
| Zhang^49^ | 2009 |  | Serology (antibody) | 1 | 1 | 1 | 1 |
| Fang^50^ | 2010 | 2008-03 to 2009-07 | Urea breath test | 1 | 1 | 0 | 1 |
| Peng^51^ | 2010 | 2003 | Serology (antibody) | 1 | 0 | 0 | 1 |
| Tong^52^ | 2010 | 2007-06 to 2010-06 | Urea breath test | 1 | 1 | 1 | 1 |
| Wang^53^ | 2010 | 2006-07 - 2009-07 | Urea breath test | 1 | 1 | 1 | 1 |
| Wu ^54^ | 2010 | 2007-04 - 2008-10 | Urea breath test | 1 | 1 | 1 | 1 |
| Chen^55^ | 2011 | 2009 | Serology (antibody) | 1 | 1 | 1 | 1 |
| Chen ^56^ | 2011 | 2010-12 to 2011-10 | Urea breath test | 1 | 1 | 1 | 1 |
| Deng ^57^ | 2011 | 2008-2010 | Serology (antibody) | 1 | 1 | 1 | 1 |
| Zou^58^ | 2011 | 2007 - 2008 | Serology (antibody) | 1 | 0 | 0 | 1 |
| Qian ^59^ | 2011 | 2010.6-2010.12 | Serology (antibody) | 1 | 1 | 1 | 1 |
| Ran ^60^ | 2011 | 2009 | Serology (antibody) | 1 | 1 | 0 | 1 |
| Chen ^61^ | 2012 | 2009 - 2010 | Serology (antibody) | 1 | 1 | 1 | 1 |
| Hua^62^ | 2012 | 2009-08 to 2011-05 | Biopsy/Histology; Urea breath test | 1 | 1 | 0 | 1 |
| Huang^63^ | 2012 | 2008-12 to 2010-06 | Urea breath test | 1 | 1 | 1 | 1 |
| Li^64^ | 2012 | 2010-06-01 to 2011-06-01 | Serology (antibody) | 1 | 1 | 1 | 1 |
| Liu ^65^ | 2012 | 2009-2011 | Serology (antibody) | 1 | 1 | 1 | 1 |
| Liu ^66^ | 2012 | 2011-05-2012-04 | Serology (antibody) | 1 | 1 | 1 | 1 |
| Sun ^67^ | 2012 | 2011.04-2011.07 | Urea breath test | 1 | 1 | 1 | 1 |
| Wan ^68^ | 2012 | 2008-2010 | Urea breath test | 1 | 1 | 1 | 1 |
| Wang^69^ | 2012 | 2011- 2012 | Urea breath test | 1 | 1 | 1 | 1 |
| Yan^70^ | 2012 | Feb 2010 - Jun 2011 | Urea breath test | 1 | 1 | 1 | 1 |
| Zhao^71^ | 2012 | 2010.01-2010.07 | Urea breath test | 1 | 1 | 1 | 1 |
| Zheng^72^ | 2012 |  | Serology (antibody) | 1 | 0 | 0 | 1 |
| Zhou^73^ | 2012 | 2011 | Urea breath test | 1 | 1 | 1 | 1 |
| Zhu^74^ | 2012 | 2010-2011 | Serology (antibody) | 1 | 1 | 0 | 1 |
| Guo^75^ | 2013 | 2012-10 – 2013-03 | Serology (antibody); Urea breath test | 1 | 1 | 0 | 1 |
| Huang ^76^ | 2013 | 2011 | Serology (antibody) | 1 | 1 | 1 | 1 |
| Mai ^77^ | 2013 | 2009-2012 | Serology (antibody) | 1 | 1 | 1 | 1 |
| Wang^78^ | 2013 | 2012 | Urea breath test | 1 | 0 | 0 | 1 |
| Yang ^79^ | 2013 | 2011 | Urea breath test | 1 | 1 | 1 | 1 |
| Zhao^80^ | 2013 | 2010-06 to 2012-06 | Urea breath test | 1 | 1 | 1 | 1 |
| Shen^81^ | 2013 | 2012 | Urea breath test | 1 | 1 | 1 | 1 |
| Cui ^82^ | 2014 | 2007-09-2011-08 | Urea breath test | 1 | 1 | 1 | 1 |
| Duan ^83^ | 2014 | 2013 | Urea breath test | 1 | 1 | 1 | 1 |
| Liang ^84^ | 2014 | 2012 | Urea breath test | 1 | 1 | 1 | 1 |
| Zhang^85^ | 2014 | 2010 | Serology (antibody) | 1 | 0 | 0 | 1 |
| Tang ^86^ | 2014 | 2009-2012 | Urea breath test | 1 | 1 | 1 | 1 |
| Wang ^87^ | 2014 | 2009-10/2010-12 | Stool antigen test | 1 | 1 | 1 | 1 |
| Wang ^88^ | 2014 | 2013 | Serology (antibody) | 1 | 1 | 0 | 1 |
| Wei ^89^ | 2014 | 2012-2013 | Serology (antibody) | 1 | 0 | 0 | 0 |
| Zhu^90^ | 2014 | 2009 - 2011 | Urea breath test | 1 | 0 | 0 | 0 |
| Zhang ^91^ | 2014 | 2012 | Serology (antibody) | 1 | 0 | 0 | 1 |
| Ding^92^ | 2015 | 2009-2011 | Stool antigen test | 0 | 0 | 0 | 1 |
| Kan^93^ | 2015 | 2013 | Serology (antibody) | 1 | 1 | 1 | 1 |
| Jiang ^94^ | 2015 | 2012-12_2013-11 | Serology (antibody) | 1 | 1 | 1 | 1 |
| Li ^95^ | 2015 | 2013-08 to 2014-12 | Serology (antibody) | 1 | 1 | 0 | 1 |
| Xu^96^ | 2015 | 1986-1989 | Serology (antibody) | 1 | 0 | 0 | 0 |
| Wang ^97^ | 2015 | 2013-01-01 to 2014-08-31 | Urea breath test | 1 | 1 | 1 | 1 |
| Zhang ^98^ | 2015 | 2009-2013 | Serology (antibody) | 1 | 1 | 1 | 1 |
| Chen ^99^ | 2016 | 2012-01_2015-10 | Urea breath test | 1 | 1 | 1 | 1 |
| Huang ^100^ | 2016 | 2014 | Serology (antibody); Urea breath test | 1 | 1 | 1 | 0 |
| Ji ^101^ | 2016 | 2013-09 to 2015-09 | Serology (antibody) | 1 | 1 | 1 | 1 |
| Jia ^102^ | 2016 | 2008- 2012 | Serology (antibody) | 1 | 1 | 1 | 1 |
| Li ^103^ | 2016 | 2013 | Serology (antibody) | 1 | 1 | 1 | 1 |
| Li ^104^ | 2016 | 2012-01-01 to 2015-01-31 | Urea breath test | 1 | 1 | 1 | 1 |
| Pan ^105^ | 2016 | 2011-2013 | Urea breath test | 1 | 0 | 0 | 1 |
| Tian ^106^ | 2016 | 2013-2014 | Urea breath test | 1 | 1 | 1 | 1 |
| Wang ^107^ | 2016 | 2013-2014 | Urea breath test | 1 | 1 | 1 | 1 |
| Wen ^108^ | 2016 | 2016 | Urea breath test | 1 | 1 | 1 | 1 |
| Yu ^109^ | 2016 | 2014-05 – 2015-07 | Urea breath test | 1 | 1 | 1 | 1 |
| Ayinuer ^110^ | 2017 | 2014-06 to 2015-05 | Urea breath test | 1 | 1 | 1 | 1 |
| Cao ^111^ | 2017 | 2012-01 to 2015-12 | Serology (antibody) | 1 | 1 | 0 | 1 |
| Li ^112^ | 2017 |  | Urea breath test | 1 | 1 | 1 | 1 |
| Liu^113^ | 2017 | 2014 | Urea breath test | 1 | 1 | 1 | 1 |
| Lu ^114^ | 2017 | 2013 -2015 | Serology (antibody); Stool antigen test; Urea breath test | 1 | 1 | 1 | 1 |
| Xu^115^ | 2017 | 2012-2016 | Serology (antibody) | 1 | 1 | 1 | 1 |
| Qu ^116^ | 2017 | 2014-01-01 to 2015-12-31 | Urea breath test | 1 | 1 | 1 | 1 |
| Shan ^117^ | 2017 | 2010-2011 | Serology (antibody) | 1 | 1 | 1 | 1 |
| Yu^118^ | 2017 | April 2013-June 2016 | Serology (antibody) | 1 | 1 | 1 | 1 |
| Zhang ^119^ | 2017 | 2015-07 – 2016-06 | Urea breath test | 1 | 1 | 1 | 1 |
| Zhao^120^ | 2017 | 2009-2010 | Stool antigen test | 1 | 1 | 0 | 1 |
| Hu^121^ | 2018 | July 2015 to December 2016 | Serology (antibody) | 1 | 1 | 0 | 1 |
| Gao ^122^ | 2018 | 2016-10_2017-10 | Urea breath test | 1 | 1 | 1 | 1 |
| Xu^123^ | 2018 | 2012-2015 | Serology (antibody) | 1 | 1 | 1 | 1 |
| Meng ^124^ | 2018 | 2017 | Serology (antibody) | 1 | 1 | 1 | 1 |
| Su^125^ | 2018 | 2015-2016 | Urea breath test | 1 | 1 | 1 | 1 |
| Sun ^126^ | 2018 | 2016-09-01_2016-12-31 | Serology (antibody) | 1 | 1 | 0 | 1 |
| Wei ^127^ | 2018 | 2016 | Urea breath test | 1 | 1 | 1 | 1 |
| Yu ^128^ | 2018 | 2014-05-05 to 2015-07-28 | Urea breath test | 1 | 1 | 1 | 1 |
| Zhou^129^ | 2018 | 2014 | Urea breath test | 1 | 1 | 1 | 1 |
| Hou^130^ | 2019 | 2015 | Urea breath test | 1 | 1 | 1 | 1 |
| Hu ^131^ | 2019 | 2016 - 2017 | Urea breath test | 1 | 1 | 1 | 1 |
| Shu^132^ | 2019 | 2015-2016 | Urea breath test | 1 | 1 | 0 | 1 |
| Zhang ^133^ | 2019 | 2017-2018 | Urea breath test | 1 | 1 | 1 | 0 |
| Gao^134^ | 2020 | January 2013 to September 2017 | Serology (antibody) | 1 | 1 | 1 | 1 |
| Zhu^135^ | 2020 | 2017 | Urea breath test | 1 | 0 | 0 | 0 |
| Hu^136^ | 2020 | 2018 | Urea breath test | 1 | 1 | 0 | 1 |
| Zhang^137^ | 2020 | 2017-2019 | Urea breath test | 1 | 1 | 1 | 1 |
| Wang^138^ | 2020 | 2018 | Urea breath test | 1 | 1 | 1 | 1 |
| Liu^139^ | 2021 | 2019 | Urea breath test | 1 | 1 | 1 | 1 |
| Shen^140^ | 2021 | June 2018 to December 2019 | Urea breath test | 1 | 1 | 1 | 1 |
| Wang^141^ | 2021 | 2016-2017 | Stool antigen test | 1 | 0 | 0 | 0 |
| Li^142^ | 2022 | 2020-2021 | Urea breath test | 1 | 1 | 1 | 1 |
| Feng ^143^ | 2022 | 2020 | Urea breath test | 1 | 1 | 1 | 1 |
| Zhou^144^ | 2022 | 2014 | Urea breath test | 1 | 1 | 1 | 1 |
| Luo ^145^ | 2022 | 2021-08-2022-05 | Urea breath test | 1 | 1 | 0 | 0 |
| Shi ^146^ | 2022 | 2020 | Urea breath test | 1 | 1 | 1 | 1 |
| Wang ^147^ | 2022 | 2019-06-2020-06 | Urea breath test | 1 | 1 | 0 | 0 |
| Xue ^148^ | 2022 | 2020-05-2020-10 | Urea breath test | 1 | 1 | 0 | 0 |
| Wu^149^ | 2022 | 2020 - 2021 | Urea breath test | 1 | 1 | 1 | 1 |
| Zhou^150^ | 2022 | 2017 and 2022 | Urea breath test | 1 | 1 | 0 | 1 |
| Chang ^151^ | 2023 | 2022 | Urea breath test | 1 | 0 | 0 | 0 |
| Chen ^152^ | 2023 | 2020-2022 | Urea breath test | 1 | 1 | 1 | 1 |
| Chen ^153^ | 2023 | August 2022 to April 2023 | Urea breath test | 1 | 0 | 0 | 0 |
| Liao^154^ | 2023 | 2015-2016 | Serology (antibody) | 1 | 0 | 0 | 0 |
| Jiang ^155^ | 2023 | 2022 | Urea breath test | 1 | 1 | 1 | 1 |
| Zou^156^ | 2023 | 2009-2010, 2013-2014, 2019-2021 | Urea breath test | 1 | 1 | 1 | 1 |
| Lan ^157^ | 2023 | 2018-2022 | Serology (antibody) | 1 | 1 | 1 | 1 |
| Liu ^158^ | 2023 | 2021-08-2023-05 | Urea breath test | 1 | 1 | 1 | 1 |
| Tong ^159^ | 2023 |  | Urea breath test | 1 | 1 | 0 | 1 |
| Wang ^160^ | 2023 | 2022 | Urea breath test | 1 | 1 | 1 | 1 |
| Wang ^161^ | 2023 | 2020 | Serology (antibody) | 1 | 1 | 1 | 1 |
| Wang ^162^ | 2023 | 2020-2021 | Urea breath test | 1 | 1 | 1 | 1 |
| Xiao ^163^ | 2023 | 2021 | Urea breath test | 1 | 1 | 1 | 1 |
| Liu^164^ | 2023 | 2014-2018 | Urea breath test | 1 | 1 | 1 | 1 |
| Zhao ^165^ | 2023 | 2019 | Urea breath test | 1 | 1 | 1 | 0 |
| Zhou ^166^ | 2023 | 2021 | Urea breath test | 0 | 0 | 1 | 1 |
| He ^167^ | 2024 | 2022-07--2023-04-30 | Serology (antibody) | 1 | 1 | 1 | 1 |
| Wang^168^ | 2024 | 2023 | PCR/string test | 1 | 1 | 1 | 1 |
| Tang ^169^ | 2024 | 2021-03 to 2023-03 | Urea breath test | 1 | 1 | 1 | 1 |
| Wang ^170^ | 2024 | 2020 | Urea breath test | 1 | 0 | 0 | 0 |
| Wang ^171^ | 2024 | 2019-2021 | Urea breath test | 1 | 1 | 1 | 1 |
| Wu ^172^ | 2024 | 2020-2022 | Urea breath test | 1 | 1 | 1 | 0 |
| Yan^173^ | 2024 | 2015-2017 | Urea breath test | 0 | 0 | 0 | 0 |
| Jiang ^174^ | 2025 | 2022-2023 | Serology (antibody) | 1 | 0 | 0 | 1 |
| Li ^175^ | 2025 | 2023 | Urea breath test | 1 | 1 | 1 | 1 |
| Liu ^176^ | 2025 | 2022 | Urea breath test | 1 | 0 | 0 | 0 |
| Sun ^177^ | 2025 | 2020-04-2023-04 | Urea breath test | 1 | 1 | 1 | 1 |
| Tan ^178^ | 2025 | 2022-06-2023-06 | Serology (antibody) | 1 | 1 | 0 | 1 |
| Wu ^179^ | 2025 | 2021 | Urea breath test | 1 | 1 | 1 | 1 |
| Yan^180^ | 2025 | 2020-2023 |  | 1 | 1 | 1 | 1 |
| Zhang ^181^ | 2025 | 2023 | Stool antigen test | 1 | 1 | 1 | 1 |

a: read (score 1) indicates high risk, while read (score 0) indicates low risk.

### Table S10. Results of meta-analysis of corrected prevalence.

| **Stratification method** | **Prevalence type** | **Period/birth cohort** | **Age group** | **Number of studies** | **Prevalence (%, 95% CI)** | **I^2^ (%)** | **Tau^2^** |
| --- | --- | --- | --- | --- | --- | --- | --- |
| Age-period | Seroprevalence | 1980-1989 | 20-39 | 1 | 61.0 (53.2 - 68.3) |  |  |
|  |  |  | 40-59 | 4 | 69.4 (60.5 - 77.0) | 72.9 | 0.12 |
|  |  |  | 60-100 | 1 | 71.3 (62.0 - 79.1) |  |  |
|  |  | 1990-1999 | 0-9 | 12 | 41.8 (32.6 - 51.5) | 71.8 | 0.33 |
|  |  |  | 10-19 | 16 | 52.4 (43.2 - 61.5) | 81.1 | 0.46 |
|  |  |  | 20-39 | 11 | 60.6 (47.6 - 72.2) | 92 | 0.7 |
|  |  |  | 40-59 | 20 | 68.4 (62.3 - 74.0) | 71.4 | 0.25 |
|  |  |  | 60-100 | 10 | 55.0 (41.7 - 67.6) | 84.7 | 0.64 |
|  |  | 2000-2009 | 0-9 | 23 | 16.8 (11.7 - 23.5) | 75.5 | 0.67 |
|  |  |  | 10-19 | 23 | 28.0 (19.6 - 38.3) | 92.2 | 1.19 |
|  |  |  | 20-39 | 49 | 42.7 (36.4 - 49.1) | 96.5 | 0.8 |
|  |  |  | 40-59 | 47 | 55.9 (50.1 - 61.4) | 96.2 | 0.6 |
|  |  |  | 60-100 | 26 | 56.8 (48.6 - 64.8) | 93.4 | 0.67 |
|  |  | 2010-2023 | 0-9 | 6 | 22.6 (21.1 - 24.3) | 45.5 | 0 |
|  |  |  | 10-19 | 15 | 21.4 (14.6 - 30.1) | 86.9 | 0.6 |
|  |  |  | 20-39 | 33 | 25.4 (19.2 - 32.9) | 98.6 | 1.02 |
|  |  |  | 40-59 | 50 | 38.4 (32.8 - 44.4) | 98.1 | 0.72 |
|  |  |  | 60-100 | 33 | 34.8 (28.0 - 42.4) | 96.4 | 0.77 |
|  | Current-infection prevalence | 2000-2009 | 0-9 | 14 | 22.6 (12.1 - 38.2) | 92.7 | 1.71 |
|  |  |  | 10-19 | 17 | 34.2 (22.7 - 47.9) | 93.9 | 1.37 |
|  |  |  | 20-39 | 13 | 52.3 (45.0 - 59.5) | 93 | 0.27 |
|  |  |  | 40-59 | 29 | 51.4 (47.6 - 55.3) | 94.2 | 0.17 |
|  |  |  | 60-100 | 12 | 52.9 (43.1 - 62.5) | 91 | 0.44 |
|  |  | 2010-2023 | 0-9 | 14 | 21.9 (13.0 - 34.4) | 93.8 | 1.28 |
|  |  |  | 10-19 | 18 | 22.5 (15.6 - 31.2) | 93.1 | 0.86 |
|  |  |  | 20-39 | 64 | 41.1 (36.9 - 45.4) | 99.4 | 0.5 |
|  |  |  | 40-59 | 124 | 44.6 (42.3 - 47.0) | 99.1 | 0.28 |
|  |  |  | 60-100 | 87 | 41.1 (38.2 - 44.1) | 95.5 | 0.3 |
| Age-birth-cohort | Seroprevalence | 1900-1919 | 60-100 | 4 | 45.3 (30.5 - 61.0) | 71.7 | 0.31 |
|  |  | 1920-1939 | 40-59 | 7 | 68.1 (59.1 - 76.0) | 71.6 | 0.19 |
|  |  |  | 60-100 | 33 | 49.4 (41.0 - 57.8) | 94.2 | 0.89 |
|  |  | 1940-1959 | 20-39 | 5 | 63.5 (46.6 - 77.7) | 82.1 | 0.53 |
|  |  |  | 40-59 | 62 | 57.5 (52.2 - 62.7) | 96.4 | 0.65 |
|  |  |  | 60-100 | 33 | 43.6 (35.2 - 52.5) | 96.4 | 0.98 |
|  |  | 1960-1979 | 10-19 | 2 | 52.4 (31.2 - 72.7) | 79.4 | 0.32 |
|  |  |  | 20-39 | 59 | 42.8 (37.0 - 48.9) | 97.8 | 0.84 |
|  |  |  | 40-59 | 52 | 41.8 (36.0 - 47.8) | 98.2 | 0.77 |
|  |  | 1980-1999 | 0-9 | 31 | 26.3 (19.8 - 34.0) | 82.1 | 0.83 |
|  |  |  | 10-19 | 45 | 35.5 (28.6 - 43.0) | 91.4 | 1.03 |
|  |  |  | 20-39 | 30 | 26.7 (19.5 - 35.4) | 98.3 | 1.22 |
|  |  | 2000-2023 | 0-9 | 10 | 17.2 (11.8 - 24.4) | 51.4 | 0.21 |
|  |  |  | 10-19 | 7 | 14.8 (10.1 - 21.2) | 49.6 | 0.15 |
|  | Current-infection prevalence | 1900-1919 | 60-100 | 1 | 74.6 (68.0 - 80.3) |  |  |
|  |  | 1920-1939 | 60-100 | 27 | 46.4 (40.2 - 52.7) | 89.9 | 0.4 |
|  |  | 1940-1959 | 40-59 | 37 | 49.1 (45.2 - 52.9) | 95.5 | 0.22 |
|  |  |  | 60-100 | 71 | 40.5 (37.4 - 43.6) | 95.6 | 0.28 |
|  |  | 1960-1979 | 20-39 | 30 | 51.8 (44.9 - 58.7) | 98.7 | 0.58 |
|  |  |  | 40-59 | 115 | 45.0 (42.6 - 47.4) | 99.2 | 0.28 |
|  |  | 1980-1999 | 0-9 | 9 | 19.1 (15.4 - 23.5) | 40.1 | 0.06 |
|  |  |  | 10-19 | 25 | 29.5 (20.2 - 40.9) | 95.4 | 1.55 |
|  |  |  | 20-39 | 46 | 38.1 (34.3 - 42.1) | 99.3 | 0.32 |
|  |  |  | 40-59 | 1 | 32.1 (29.0 - 35.4) |  |  |
|  |  | 2000-2023 | 0-9 | 19 | 23.9 (13.2 - 39.3) | 94.7 | 2.21 |
|  |  |  | 10-19 | 10 | 23.7 (17.6 - 31.1) | 81.6 | 0.3 |
|  |  |  | 20-39 | 1 | 20.3 (16.8 - 24.4) |  |  |

### Table S11. Results of meta-analysis of reported prevalence

| Stratification method | Prevalence type | Period/birth cohort | Age group | Number of studies | Prevalence (%, 95% CI) | I^2^ (%) | Tau^2^ |
| --- | --- | --- | --- | --- | --- | --- | --- |
| Age-period | Seroprevalence | 1980-1989 | 20-39 | 1 | 55.8 (50.7 - 60.7) |  |  |
|  |  |  | 40-59 | 5 | 66.4 (55.3 - 75.9) | 95.3 | 0.27 |
|  |  |  | 60-100 | 1 | 62.5 (56.7 - 68.0) |  |  |
|  |  | 1990-1999 | 0-9 | 13 | 36.7 (25.4 - 49.7) | 96.2 | 0.91 |
|  |  |  | 10-19 | 16 | 50.2 (44.3 - 56.1) | 87.5 | 0.19 |
|  |  |  | 20-39 | 11 | 55.1 (47.1 - 62.9) | 93.2 | 0.27 |
|  |  |  | 40-59 | 20 | 63.0 (58.2 - 67.5) | 91.1 | 0.18 |
|  |  |  | 60-100 | 11 | 54.9 (44.8 - 64.7) | 92.1 | 0.44 |
|  |  | 2000-2009 | 0-9 | 30 | 22.9 (19.9 - 26.0) | 88.6 | 0.2 |
|  |  |  | 10-19 | 24 | 35.7 (30.5 - 41.3) | 93.9 | 0.32 |
|  |  |  | 20-39 | 50 | 45.9 (41.0 - 51.0) | 97.8 | 0.51 |
|  |  |  | 40-59 | 47 | 55.1 (51.0 - 59.2) | 97.4 | 0.32 |
|  |  |  | 60-100 | 26 | 55.0 (49.5 - 60.5) | 95.2 | 0.31 |
|  |  | 2010-2023 | 0-9 | 9 | 21.2 (14.6 - 29.8) | 95.2 | 0.45 |
|  |  |  | 10-19 | 16 | 30.4 (24.1 - 37.5) | 92.3 | 0.38 |
|  |  |  | 20-39 | 33 | 33.2 (28.8 - 38.0) | 98.9 | 0.36 |
|  |  |  | 40-59 | 51 | 40.6 (36.8 - 44.6) | 98.6 | 0.33 |
|  |  |  | 60-100 | 39 | 44.5 (37.2 - 52.0) | 98.3 | 0.89 |
|  | Current-infection prevalence | 2000-2009 | 0-9 | 20 | 11.7 (6.7 - 19.4) | 95.9 | 1.68 |
|  |  |  | 10-19 | 17 | 32.4 (23.8 - 42.3) | 96.8 | 0.77 |
|  |  |  | 20-39 | 13 | 51.4 (44.2 - 58.5) | 94.2 | 0.26 |
|  |  |  | 40-59 | 29 | 50.2 (46.4 - 54.0) | 94.7 | 0.16 |
|  |  |  | 60-100 | 12 | 50.0 (42.0 - 57.9) | 90.3 | 0.29 |
|  |  | 2010-2023 | 0-9 | 18 | 14.6 (8.5 - 23.8) | 96.6 | 1.64 |
|  |  |  | 10-19 | 18 | 25.7 (19.6 - 33.0) | 95.2 | 0.54 |
|  |  |  | 20-39 | 64 | 41.9 (38.0 - 45.9) | 99.4 | 0.43 |
|  |  |  | 40-59 | 124 | 45.0 (42.9 - 47.2) | 99.2 | 0.23 |
|  |  |  | 60-100 | 87 | 42.3 (39.5 - 45.2) | 95.9 | 0.28 |
| Age-birth-cohort | Seroprevalence | 1900-1919 | 60-100 | 4 | 48.4 (34.9 - 62.1) | 85.1 | 0.27 |
|  |  | 1920-1939 | 40-59 | 8 | 63.9 (55.4 - 71.6) | 92.7 | 0.24 |
|  |  |  | 60-100 | 37 | 53.7 (47.1 - 60.2) | 97.5 | 0.65 |
|  |  | 1940-1959 | 20-39 | 5 | 57.5 (46.9 - 67.4) | 86.7 | 0.21 |
|  |  |  | 40-59 | 62 | 55.8 (51.9 - 59.6) | 97.9 | 0.38 |
|  |  |  | 60-100 | 36 | 46.1 (39.4 - 52.9) | 97.9 | 0.68 |
|  |  | 1960-1979 | 10-19 | 2 | 50.1 (36.1 - 64.0) | 79.5 | 0.14 |
|  |  |  | 20-39 | 59 | 45.8 (41.4 - 50.2) | 98.5 | 0.47 |
|  |  |  | 40-59 | 53 | 43.2 (39.3 - 47.3) | 98.7 | 0.36 |
|  |  | 1980-1999 | 0-9 | 37 | 28.3 (23.8 - 33.4) | 94 | 0.5 |
|  |  |  | 10-19 | 47 | 39.5 (35.1 - 44.1) | 95.1 | 0.4 |
|  |  |  | 20-39 | 31 | 34.3 (29.1 - 39.9) | 98.9 | 0.46 |
|  |  | 2000-2023 | 0-9 | 15 | 19.6 (15.2 - 25.0) | 95.6 | 0.33 |
|  |  |  | 10-19 | 7 | 26.7 (22.9 - 30.9) | 67.8 | 0.05 |
|  | Current-infection prevalence | 1900-1919 | 60-100 | 1 | 57.7 (53.0 - 62.4) |  |  |
|  |  | 1920-1939 | 60-100 | 27 | 46.2 (40.7 - 51.9) | 90.8 | 0.33 |
|  |  | 1940-1959 | 40-59 | 37 | 48.6 (45.0 - 52.3) | 96 | 0.19 |
|  |  |  | 60-100 | 71 | 41.9 (38.9 - 45.0) | 96 | 0.27 |
|  |  | 1960-1979 | 20-39 | 30 | 51.5 (45.0 - 57.9) | 98.8 | 0.51 |
|  |  |  | 40-59 | 115 | 45.3 (43.1 - 47.5) | 99.2 | 0.23 |
|  |  | 1980-1999 | 0-9 | 9 | 16.7 (13.4 - 20.6) | 53.2 | 0.08 |
|  |  |  | 10-19 | 25 | 30.1 (23.0 - 38.4) | 97.4 | 0.84 |
|  |  |  | 20-39 | 46 | 39.0 (35.5 - 42.6) | 99.4 | 0.26 |
|  |  |  | 40-59 | 1 | 29.5 (27.3 - 31.8) |  |  |
|  |  | 2000-2023 | 0-9 | 29 | 12.0 (7.2 - 19.1) | 97.1 | 2.18 |
|  |  |  | 10-19 | 10 | 26.1 (20.7 - 32.4) | 84.1 | 0.2 |
|  |  |  | 20-39 | 1 | 21.2 (18.7 - 24.0) |  |  |

### Table S12. Priors, results, and performance of four competing models

| **Models** | **Competing assumptions** | **Prior** | **Convergence (maximum rank-normalized R-hat)** | **Expected log predictive density (standard error)** | **Effective number of parameters (standard error)** |
| --- | --- | --- | --- | --- | --- |
| 1 | Constant rate from infection to symptoms + Piecewise-linear $\alpha_{c}$ | $\beta_{0}$ ~ normal(2.0, 2.0);  $m_{k}^{raw}$~ normal(0, 2.0);  log(γ) ~ normal(-1, 1);  $\rho_{logit\_fmax}$~ normal(logit(0.1), 2);  t_50_ ~ Uniform(1990,2023)  omega ~ lognormal(log(0.48), 0.4);  ϕ ~ lognormal(log(20), 1.0);  z_study ~ normal(0, 1);  $\tau$ ~ normal(0, 0.5); | Yes (1.0044) | -4009 (49) | 156(13) |
| 2 | Constant rate from infection to symptoms + I-spline $\alpha_{c}$ | $\alpha_{hi}$~ beta(6, 3);  $\alpha_{lo}$~ beta(3, 6);  w ~ uniform on simplex (implicit)  log(γ) ~ normal(-1, 1);  $\rho_{logit\_fmax}$~ normal(logit(0.1), 2);  t_50_ ~ Uniform(1990, 2023)  omega ~ lognormal(log(0.48), 0.4);  ϕ ~ lognormal(log(20), 1.0);  z_study ~ normal(0, 1);  $\tau$ ~ normal(0, 0.5); | Yes (1.0013) | -4012(50) | 157(14) |
| 3 | Infection time-dependent rate + Piecewise-linear $\alpha_{c}$ | $\beta_{0}$ ~ normal(2.0, 2.0);  $m_{k}^{raw}$~ normal(0, 2.0);  log(γ) ~ normal(-1, 1);  $\rho_{logit\_fmax}$~ normal(logit(0.5), 2);  t_50_ ~ Uniform(1990,2023)  omega ~ lognormal(log(0.48), 0.4);  ϕ ~ lognormal(log(20), 1.0);  z_study ~ normal(0, 1);  $\tau$ ~ normal(0, 0.5); | Yes (1.0015) | -4017(49) | 152(13) |
| 4 | Infection time-dependent rate + I-spline $\alpha_{c}$ | $\alpha_{hi}$~ beta(6, 3);  $\alpha_{lo}$~ beta(3, 6);  w ~ uniform on simplex (implicit)  log(γ) ~ normal(-1, 1);  $\rho_{logit\_fmax}$~ normal(logit(0.5), 2);  t_50_ ~ Uniform(1990, 2023)  omega ~ lognormal(log(0.48), 0.4);  ϕ ~ lognormal(log(20), 1.0);  z_study ~ normal(0, 1);  $\tau$ ~ normal(0, 0.5); | Yes (1.0012) | -4018(49) | 152(13) |

## Figures

### Figure S2. Age-period and age-cohort patterns of reported *Helicobacter pylori* prevalence in China, 1980-2023.

Panels A and B show pooled age-period-specific seroprevalence and current-infection prevalence, respectively. Panels C and D show corresponding pooled age-cohort-specific estimates. Lines represent pooled prevalence trends for each survey period (A, B) or birth cohort (C, D). Points indicate pooled estimates and error bars represent 95% confidence intervals. Point sizes are proportional to the number of studies contributing to each estimate.

### Figure S3. ****Comparison between observed and model-predicted**** Helicobacter pylori A) seroprevalence, B) current-infection prevalence, and C) ever-infection prevalence.

Panels show the comparison between observed data (red) and Bayesian model predictions (blue) across age groups and survey periods. Observed values represent prevalence reported in individual studies, with 95% binomial confidence intervals. Size of red points is proportional to the sample size of observed records. Model estimates show posterior medians and 95% credible intervals from the Bayesian catalytic model.

### Figure S4. ****Comparison between observed and model-predicted**** Helicobacter pylori ****for the validation target****^12^

Red points and error bars represent observed data and corresponding 95% confidence intervals. Blue points and error bars indicate estimates of Bayesian model and corresponding 95% credible intervals.

### ****Figure S5. Posterior correlations among parameters in Model 4****

Cells show Pearson correlation coefficients calculated across joint posterior draws. Red indicates positive correlation, blue negative correlation, and white values close to zero. Numerical values are displayed for |r|≥0·50 and on the diagonal. α_hi and α_lo denote the asymptotes for the earliest and latest cohorts; γ the age-related acquisition parameter; ρ_max the maximum annual treatment hazard; t₅₀ the midpoint year of treatment scale-up; ω the seroreversion rate; ϕ the beta-binomial concentration parameter; and τ_study the between-study standard deviation.

### Figure S6. Estimates of *Helicobacter pylori* dynamics from the alternative Bayesian catalytic model

Panel A shows posterior median *Helicobacter pylori* force of infection, where colors indicate the FOI (% per year), and white contour lines mark FOI levels (labelled). Panel B shows posterior median current-infection prevalence, where colors show the proportion infected, and white contour lines mark prevalence levels (labelled). In panel C and D, lines represent posterior medians of current-infection prevalence and shaded ribbons indicate 95% credible intervals.

### Figure S7. Estimates of *Helicobacter pylori* associated disease burden from the alternative Bayesian catalytic model

Panels A and B show median PAFs (solid lines) with 95% credible intervals (shaded ribbons) for gastric cancer (GC) by subsite, and peptic ulcer disease (PUD) by subtype. Panels C and D show median numbers (solid lines) with 95% uncertainty (shaded ribbons) of cases attributable to H. pylori for GC and PUD. Gray line shows total cases. Colored solid/dashed lines show attributable cases/PAF under different scenarios of subtype composition. PAF, population attributable fraction; GC, gastric cancer; PUD, peptic-ulcer disease; DU, duodenal ulcer; GU, gastric ulcer; NC, non-cardia.

## References

1.	Forman D, Sitas F, Newell D, others. Geographic association of Helicobacter pylori antibody prevalence and gastric cancer mortality in rural China. *Int J Cancer*. 1990;46:608-611. doi:10.1002/ijc.2910460410

2.	Chen JY, Zhu ZH, Shen X, Jiang LR, Zhang JG. Clinical observation on the detection of anti Helicobacter pylori antibody in 328 children. *J Clin Pediatr*. 1993;11(04):04.

3.	Zhang WD, Wu Y, Yang HT, et al. Seroepidemiological survey of Helicobacter pylori infection in natural population in the area of gastric cancer. *Chin J Dig*. 1995;15(05):05.

4.	Liu WZ, Xiao SD, Jiang SJ, others. Seroprevalence of Helicobacter pylori infection in medical staff in Shanghai. *Scand J Gastroenterol*. 1996;31:749-752. doi:10.3109/00365529609010346

5.	J-l M, W-c Y, Gail MH, others. Helicobacter pylori infection and mode of transmission in a population at high risk of stomach cancer. *Int J Epidemiol*. 1998;27:570-573. doi:10.1093/ije/27.4.570

6.	Zhang L, Ma JJ, Pan KF, et al. Studies on infection of Helicobacter pylori and their subtypes cagA+ and hspA+ in population living in areas with high and low incidence of gastric cancer. *Chin J Prev Med*. 1998;32(02):02.

7.	Tang GD, Tang XH, Chen ZN, Han P, Wei ZP, Chen JC. Helicobacter pylori infection in the aged in longevity area of Bama Country, Guangxi. *J Guangxi Med Univ*. 1999;16(06):06.

8.	Wong B, Lam S, Ching C, others. Differential Helicobacter pylori infection rates in two contrasting gastric cancer risk regions of South China. *J Gastroenterol Hepatol*. 1999;14:120-125. doi:10.1046/j.1440-1746.1999.01823.x

9.	Xu CD, Zhou JY, Xue Y, et al. Seroepidemiology of Helicobacter pylori infection among asymptomatic children. *Chin J Pedia*. 1999;37(07):07.

10.	Zhang XH, Sun XM, Zhao WY, others. Seroepidemiological study on Helicobactor pylori infection in rural adult residents. *Chin J Epidemiol*. 1999;20(4):212-214.

11.	Chen GG. Investigation of Helicobacter pylori infection in children and adolescents in Zhoushan Island. *Pract Prev Med*. 2000;7(05):05.

12.	Liu L, Zhang YH, Huang P, et al. An epidemiological survey of Helicobacter pylori infection in natural population of Hunan provincial capital. *Chin J Mod Med*. 2000;10(04):04.

13.	Brown LM, Thomas TL, Ma JL, others. Helicobacter pylori infection in rural China: Exposure to domestic animals during childhood and adulthood. *Scand J Infect Dis*. 2001;33:686-691. doi:10.1080/00365540110026845

14.	Zhou JY, Xue Y, Xu CD. Study on the influence of asymptomatic Helicobacter pylori infection on the growth and development of suburban children. *J Clin Pediatr*. 2001;19(03):03.

15.	Chen S, Xiao S, Liu W, Xu W, Pan Y. Comparison of seroepidemiology of Helicobacter pylori in Shanghai urban area during 1990 and 2001. *Chin J Gastroenterol*. 2002;7(03):03.

16.	Niu HB. Seroepidemiology of Helicobacter pylori infection among preschool children and risk factors for infection. *Pract Prev Med*. 2003;10(01):01.

17.	Chen S, Xiao S. Seroepidemiological comparison of Helicobacter pylori infection rates in Shanghai urban districts in 1990 and 2001. *Chin J Dig Dis*. 2003;4:40-44. doi:10.1046/j.1443-9573.2003.00111.x

18.	Wang M, Rao SL, Ruan GT, Hu S, Xu JZ. Epidemiological study on infectious status of Helicobacter pylori in children in Haikou City. *China Trop Med*. 2003;3(05):05.

19.	Xue Y, Ruan S. The effect of asymptomatic Helicobacter pylori infection on hemoglobin in children. *J Xinjiang Med Univ*. 2003;26(02):02.

20.	Zeng ZR, Hu PJ, Hu S, others. Association of interleukin 1B gene polymorphism and gastric cancers in high and low prevalence regions in China. *Gut*. 2003;52:1684-1689. doi:10.1136/gut.52.12.1684

21.	Zhang LX, Zhang L, Zhang NX, et al. The analysis of Helicobacter pylori infection on cytotoxin associated gene A in fection in children under 14 years old. *Chin J Coal Ind Med*. 2003;6(10):10.

22.	Li HQ, Li Q, Liu YM. Epidemiological investigation of HP infection in children in Baoji City. *Chin J Contemp Pediatr*. 2004;6(05):05.

23.	Mei BR, Qian J, Tang GR, Zhang J, Xie GQ. Epidemiological investigation of Helicobacter pylori infection and its correlative factors in children in Wuxi area. *J Clin Pediatr*. 2004;22(10):10.

24.	Wang LL. [Association of HLA-DQA1 loci with Helicobacter pylori infection in children of Guangxi Zhuang, Mulam nationalities, China and central Jing nationality, Vietnam]. *Zhonghua Er Ke Za Zhi Chin J Pediatr*. 2004;42(8):8.

25.	Wang XF, Wang KX, Li ZP. Investigation on Helicobacter pylori infection and its related diseases in Huainan city. *J Trop Dis Parasitol*. 2004;2(02):02.

26.	Zhuang ML, Huang LP, Li RJ, Bei GP. Study on the relationship between iron deficiency, iron deficiency anemia and Helicobacter pylori infection in island children. *Chin Prev Med*. 2004;5(06):06.

27.	Cao P, Lu QM, Zhang XL, et al. Epidemiological study of Helicobacter pylori infection in Tianzhu and Lanzhou areas. *Chin J Gastro Hepa*. 2005;14(02):02.

28.	Chen SY, Liu TS, Fan XM, et al. Epidemiological study of Helicobacter pylori infection and its risk factors in Shanghai. *Natl Med J China*. 2005;85(12):12.

29.	Gu JL, Li BC, Fang N. An Analysis of Helicobacter pylori infection among 300 children in Kaifeng. *Chin J Sch Health*. 2005;26(07):07.

30.	Liu B, Fan YJ, Wang ML, others. Genetic polymorphisms in glutathione S-transferases T1, M1 and P1 and susceptibility to reflux esophagitis. *Esophagus*. 2006;19:477-481. doi:10.1111/j.1442-2050.2006.00609.x

31.	Xiang P, Yang WG, Li K, Zhou MH, Li XF, Zhou JD. Epidemiological investigation of Helicobacter pylori infection in adults of different nationalities in Xiangxi, Hunan. *Pract Prev Med*. 2006;13(01):01.

32.	Chen J, Bu XL, Wang QY, Hu PJ, Chen MH. Decreasing seroprevalence of Helicobacter pylori infection during 1993-2003 in Guangzhou, southern China. *Helicobacter*. 2007;12(02):02. doi:10.1111/j.1523-5378.2007.00487.x

33.	Lu P, Zhou L, Zhang YH. Epidemic study on Helicobacter pylori among old people and their families in Guiyang. *Chin J Public Health*. 2007;23(03):03.

34.	Nie XR, Zhou T, Pan KF, others. The associations of serum gastrin level with Helicobacter pylori infection. *Chin J Prev Med*. 2007;41Suppl:87-89.

35.	Xu LF, Chen L, Wang KX, Li ZP. Investigation of Helicobacter pylori infection in college students. *Chin J Zoonoses*. 2007;23(12):12.

36.	Shi R, Xu S, Zhang H, others. Prevalence and risk factors for Helicobacter pylori infection in Chinese populations. *Helicobacter*. 2008;13:157-165. doi:10.1111/j.1523-5378.2008.00586.x

37.	Cheng H, Hu F, Zhang L, others. Prevalence of Helicobacter pylori infection and identification of risk factors in rural and urban Beijing, China. *Helicobacter*. 2009;14:128-133. doi:10.1111/j.1523-5378.2009.00668.x

38.	Guo XW, Wang J, Feng H, Ablickwood XAY, Liao QS. The epidemiological lunestiqation of Helicobacter pylori infection in the region of Aksn, Xinjiang. *J Xinjiang Med Univ*. 2009;32(06):06.

39.	He JQ, Xia SH, Yu . H. G., et al. Toxigenic cobacter pylori infection among adult population in Futian district, Shenzhen: an epidemiological investigation. *Chin J Nosocomiol*. 2009;19(24):24.

40.	Li L, Zhu FC, Mao XH, et al. Investigation on Helicobacter pylori infection in rural children in Ganyu county. *Chin J Public Health*. 2009;25(05):05.

41.	Wang JF, Dang MF. Analysis of the infection rate of Helicobacter pylori in 5710 healthy people in Tongchuan area. *Lab Med Clin*. 2009;6(10):10.

42.	Wu XF, Cai WW, Miao XP, Wang ZY. Epidemiological analysis of multiple factors of Helicobacter pylori infection in people aged hundred years in Hainan island. *China Trop Med*. 2009;9(05):05.

43.	Xi XY, Li SW. Epidemiological investigation of Helicobacter pylori infection in primary school students. *Med J West China*. 2009;21(02):02.

44.	Yang L, Zhou L, Zhang YH, Lei JJ. Epidemiological investigation and multi-factors analysis on the situation of Helicobacter pylori infection in Zhenning Country of Guizhou Province. *Chin J Gastroenterol Hepatol*. 2009;18(02):02.

45.	Zeng FL, Xiang L. Epidemiological survey on Helicobacter pylori infection in Chongqing city residents undergoing physical examination. *J Chongqing Med Univ*. 2009;34(02):02.

46.	Zhang DH, Zhou LY, Lin SR, others. [Epidemiology of Helicobacter pylori infection in Shandong and Beijing areas.]. *Zhonghua Nei Ke Za Zhi*. 2009;48(12):12.

47.	Zhang LH, Zhou YN, Zhang ZY, others. [Epidemiological study on status of Helicobacter pylori in children and teenagers in Wuwei city, Gansu province]. *Zhonghua Yi Xue Za Zhi*. 2009;89(38):38.

48.	Zhang TZ, Yuan JX, Xu YJ, Fan HM, Cheng JS. The studies on the status of Helicobacter pylori infection analysis and the factors ralative to infection in rural residents. *China J Mod Med*. 2009;19(22):22.

49.	Zhang Y, Zheng WB, Huang MY, Kou YM, Yang N, Han SJ. An analysis of Helicobacter pylori infection in Chengdu. *West China Med J*. 2009;24(08):08.

50.	Fang L, Wu Y, Lin YS, Zou B, Zhong LJ. Analysis of relative factors of Helicobacter pylori infection in physical examination in Shenzhen. *J Rare Uncommon Dis*. 2010;17(06):06.

51.	Peng SY, Qing CH. Survey and analysis of related factors of Helicobacter pylori infections in children in Changsha. *Chin J Mod Med*. 2010;20(03):03.

52.	Tong NS, Shi RC. Analysis on the results of Helicobacter pylori infection in 2239 physical examinees. *Labeled Immunoass Clin Med*. 2010;17(04):04.

53.	Wang J, Ding CS, Gao SY. Helicobacter pylori infection in children is associated with family members. *Chin J Clin Lab Sci*. 2010;28(04):04.

54.	Wu YL, Ye H, Li AP. Analysis of detection results of Helicobacter pylori infection in physical examination population. *Chin J Health Care Med*. Published online 2010:02.

55.	Chen S, Yang G, Wu ZC, Li M. Seroepidemiological analysis of Helicobacter pylori infection in physical examination population in Foshan area. *Guangdong Med J*. 2011;32(07):07.

56.	Chen XH, Li YH, Ren K, Zhang Y. Helicobacter pylori infection in health check group. *China Pr Med*. 2011;6(35):35.

57.	Deng XJ, Qu XY, Xiang L, Guo L. Epidemiological survey on Helicobacter pylori infection in the residents undergoing physical examination in the three years. *Chin J Lab Diagn*. 2011;15(12):12.

58.	Zou D, He J, Ma X, others. Helicobacter pylori infection and gastritis: the Systematic Investigation of gastrointestinaL diseases in China (SILC). *J Gastroenterol Hepatol*. 2011;26:908-915. doi:10.1111/j.1440-1746.2010.06608.x

59.	Qian M, Yuan JJ, Lin YY. Analysis of Helicobacter pylori infection status of 2840 physical examinees in Zhuhai. *Lab Med Clin*. 2011;8(22):22.

60.	Ran L, Deng XJ, Qu XY, You JR, Wang YH, Luo R. A study on the infection of Helicobacter pylori among occupational population in Chongqing and relationship between Helicobacter pylori and BMI. *Chin Gen Pract*. 2011;14(06):06.

61.	Chen XF. Analysis of HP infection among health examination participants in Bao’an district of Shenzhen City. *J Chengdu Med Coll*. 2012;7(03):03.

62.	Hua ZL, Zhu YC, Guo GP, et al. An analysis of Helicobacter pylori infection in high incidence area of upper digestive tract cancer in Yangzhou city. *China Cancer*. 2012;21(09):09.

63.	Huang Y, Xu Y, Zeng L, others. Investigation on the H. pylori infection and risk factors among physical examinees in Chengdu. *Mod Prev Med*. 2012;39:3494-3497.

64.	Li J, Yuan Y, Huang Y, Hu JG, Zheng ZJ. Analysis of Helicobacter pylori infection rate among healthy population. *J Bengbu Med Coll*. 2012;37(11):11.

65.	Liu QD, Zhang SH, Huang HK. 524 cases of elderly healthy physical examinees and provide the basis for the healthy physical examinees. *China Pr Med*. 2012;7(24):24.

66.	Liu Y, Wang J, Xing RQ, others. Analysis investigation on infection status and the related factors of Helicobacter pylori in health adult group in Xi’an area. *J Mod Lab Med*. 2012;27(79-82):79-82.

67.	Sun S, Feng F. Study on the positive results of 758 cases of 14C urea breath test in Suzhou. *Lab Med Clin*. 2012;9(10):10.

68.	Wan HY, Liu JS, Tian Y, Zhang WY. Investigation on Helicobacter pylori infection in middle-age people undergoing physical examination in Minhang district of Shanghai. *J Pr Med*. 2012;28(10):10.

69.	Wang H, Huang JF, Zhao HY, Liang XY. Investigation of Helicobacter pylori infection in physical examination people. *Chin J Health Stat*. 2012;29(05):05.

70.	Yan H, Qiu XG. Analysis of Helicobacter pylori infections in 2857 healthy persons receiving physical examination. *Chin J Nosocomiol*. 2012;22(02):02.

71.	Zhao WD, Jing Y, Zhang G, Liu HL, Chen QC. Investigation on Helicobacter pylori infection status of middle-aged and young people in shengli oil field. *Mod Prev Med*. 2012;39(01):01.

72.	Zheng KC, Aoki K, Li XQ, others. Serum pepsinogens, gastrin-17 and Helicobacter pylori antibody in the residents of two cities in china with distinct mortality rates of gastric cancer. *Tohoku J Exp Med*. 2012;228:289-294. doi:10.1620/tjem.228.289

73.	Zhou H, Chen ZH, Wang YQ, Zhu XL, Liu YX. Analysis of the influencing factors of Helicobacter pylori infection in healthy people. *Pract Prev Med*. 2012;19(11):11.

74.	Zhu QF, Yu . Q., Zhao X, Chen ZH, Xu HX. The study on information of Helicobacter pylori infection in healthy people in Suzhou. *Lab Med Clin*. 2012;9(18):18.

75.	Guo XY, Wang T, Dong L, Shi HT. Epidemiological survey on Helicobacter pylori infection among rural population of South and North of Shaanxi Province. *J Hainan Med Univ*. 2013;19(10):10.

76.	Jiang H, Yang QW, Liu P, Wang QY, Ye ZW, Zhu ZJ. Analysis of 2808 cases of Helicobacter pylori infection. *Labeled Immunoass Clin Med*. 2013;20(02):02.

77.	Mai YK, Liang Z, Liang LQ, He D, Zhao ZJ. Study on Helicobacter pylori and its correlative factors in school-age children in Gaoyao city. *Natl Med Front China*. 2013;8(04):04.

78.	Wang H, Li HP, Huang SP. Helicobacter pylori infection and its influencing factors among peasants in northern Jiangsu province. *Chin J Public Health*. 2013;29(01):01.

79.	Yang XJ, Wang GJ, Wu JX, Gong MY. Analysis of detection results of Helicobacter pylori in physical examination people. *Chin J Lab Diagn*. 2013;17(07):07.

80.	Zhao D, Wang SJ, Zhang J, Zhang BJ. Epidemiological investigation of Helicobacter pylori infection in children in Xinxiang areas. *J Xinxiang Med Univ*. 2013;30(03):03.

81.	Shen Z, Ye Q, Liu Y, others. Helicobacter pylori infection is associated with the presence of thyroid nodules in the euthyroid population. *PLoS One*. 2013;8:e80042. doi:10.1371/journal.pone.0080042

82.	Cui WH, Li CH. Characteristics of Helicobacter pylori infection in middle-age and elderly people in Yanbian area. *Chin J Gerontol*. 2014;34(10):10.

83.	Duan CL, Tang L. Analysis of 13C urea breath test results of Mongolian and Han nationality in Baotou, Neimenggu. *Chin Pr Med*. 2014;39(09):09.

84.	Liang FR, Jia YX, Dong HX. Analysis of Helicobacter pylori infections detected by 14C urea breath test. *Chin J Nosocomiol*. 2014;24(10):10.

85.	Zhang M, Zhou YZ, Li XY, others. Seroepidemiology of Helicobacter pylori infection in elderly people in the Beijing region, China. *World J Gastroenterol*. 2014;20:3635. doi:10.3748/wjg.v20.i13.3635

86.	Tang HR, Fan YJ, Liu S. Helicobacter pylori infection and associated risk factors in Chengdu. *J Sichuan Univ Med Sci Edi*. 2014;45(05):05.

87.	Wang LY, Mao M, Wang ZL, Gao S. Investigation of Helicobacter pylori infection in children in Chengdu City. *J Prev Med Inf*. 2014;30(05):05.

88.	Wang T, Wang YP, Ren YY, et al. Analysis of survey of AHeqi County with Khalkhas of Helicobacter pylori. *Chin J Lab Diagn*. 2014;18(04):04.

89.	Wei ZH, Feng SH, Zhang ZF, et al. Epidemiological investigation of Helicobacter pylori infection in Zhuang population in Chongzuo rural area. *Chin Med Equip*. 2014;11(S1):S1.

90.	Zhu Y, Zhou X, Wu J, others. Risk factors and prevalence of Helicobacter pylori infection in persistent high incidence area of gastric carcinoma in Yangzhong city. *Gastroenterol Res Pr*. 2014;2014. doi:10.1155/2014/481365

91.	Zhang XJ, Huang YH, Yang L, et al. Epidemiology investigation on the Helicobacter pylori infection of pre-school children in Shenyang. *Chin J Child Health Care*. 2014;22(04):04.

92.	Ding Z, Zhao S, Gong S, et al. Prevalence and risk factors of Helicobacter pylori infection in asymptomatic Chinese children: a prospective, cross-sectional, population-based study. *Aliment Pharmacol Ther*. 2015;42(08):08. doi:10.1111/apt.13364

93.	Kan BH, Sun LH, Wu CH. Analysis of results of Helicobacter pylori infection in physical examination people. *Int J Lab Med*. 2015;36(21):21.

94.	Jiang FQ, Hao CY, Lu XY, et al. Helicobacter pylori infection in healthy population in Dalian city. *J Dalian Med Univ*. 2015;37(02):02.

95.	Li XL, Dai GZ, Huang CH. Analysis of the related factors of Helicobacter pylori infection in the physical examination population in Chenzhou area. *Int J Lab Med*. 2015;36(19):19.

96.	Xu L, Qu YH, Chu XD, others. Urinary levels of N-nitroso compounds in relation to risk of gastric cancer: findings from the shanghai cohort study. *PLoS One*. 2015;10. doi:10.1371/journal.pone.0117326

97.	Wang Q, Chu NJ, Chen TT, Jia DM. Analysis on the status of Helicobacter pylori infection in adults in Yunnan plateau area. *J Kunming Med Univ*. 2015;36(10):10.

98.	Zhang YP, Xiong L, He JT, Zhang L, Wang JH, Huang L. Analysis of Helicobacter pylori infection in physical examination population in Xi’an. *Int J Lab Med*. 2015;36(01):01.

99.	Chen L, Jia XM, Wang SX. Analysis of 8809 cases of Helicobacter pylori in Beijing miyun area. *Mod Dig Interv*. 2016;21(02):02.

100.	Huang J, Zhou Y, Huang Y, others. [Prevalence of Helicobacter pylori infection among children aged 7 to 18 years old: a school-based cross-sectional study in Minhang district of Shanghai in 2014]. *Zhonghua Er Ke Za Zhi Chin J Pediatr*. 2016;54(7):7.

101.	Ji N, Gu YN. Prevalence of Helicobacter pylori infection among the healthy asymptomatic population. *Labeled Immunoass Clin Med*. 2016;23(03):03.

102.	Jia ZF, Wang LZ, Cao XY, others. CD24 genetic variants contribute to overall survival in patients with gastric cancer. *World J Gastroenterol*. 2016;22:2373. doi:10.3748/wjg.v22.i7.2373

103.	Li GY, Su GL. The relationship of Helicobacter pylori infection and FPG, lipid, glucose, uric acid, PLT, PDW and MPV in serum among health population. *Int J Lab Med*. 2016;37(18):18.

104.	Li H, Zhang ZF, Lu R, Jiang Y, Li Y. Prevalence of Helicobacter pylori infections among physical examination population in Hangzhou area. *Chin J Nosocomiol*. 2016;26(09):09.

105.	Pan KF, Zhang L, Gerhard M, others. A large randomised controlled intervention trial to prevent gastric cancer by eradication of Helicobacter pylori in Linqu county, China: Baseline results and factors affecting the eradication. *Gut*. 2016;65:9-18. doi:10.1136/gutjnl-2015-309197

106.	Tian Y, Cui XM, Qi SN, Zhang ZH. Analysis of Helicobacter pylori infection of physical examination population in Beijing Yongwai Region from 2013-2014. *China Med Her*. 2016;13(06):06.

107.	Wang HK, Yao P. Regression analysis of risk factors for HP infection in Uyghur, Kazak and Han nationality people. *China Pr Med*. 2016;11(25):25.

108.	Wen LN, Liu L. Analysis of test results of Helicobacter pylori in 3733 physical examinees. *Int J Lab Med*. 2016;37(24):24.

109.	Yu . X. H., Zhao HZ, Li XY, Gao J, He YG, Sun YR. Survey of Helicobacter pylori infection in physical examination population in Lanzhou, Gansu province. *Chin J Gastroenterol*. 2016;21:687-689.

110.	Ayinuer AHM, Kaisa NYZ, Gao F. Helicobacter pylori infection healthy uyghur and Han population in Xinjiang. *J Trop Med*. 2017;17(10):10.

111.	Cao C, Zhou XH, Hu GY, Qin XS, Li GZ, Huang ZS. Investigation of the status quos of Helicobacter pylori infection in people of the area bordering Yunnan, Guizhou and Guangxi. *J Pr Med*. 2017;33(11):11.

112.	Li XY, Zhao HZ, Yu . X. H., He YG, Tao H, Wang W. Investigation and analysis of Helicobacter pylori infection in different occupational healthy population in Lanzhou. *Chin J Gastroenterol Hepatol*. 2017;26(01):01.

113.	Liu J, Wang Y, Zhao Q, others. Prevalence and risk factors for Helicobacter pylori infection in southwest China: a study of health examination participants based on 13C-urea breath test. *Turk J Med Sci*. 2017;47:1456-1462. doi:10.3906/sag-1605-149

114.	Lu LJ, Jia HF, Cheng SX, Yu . D. L., Chen T. Status of Helicobacter pylori infection in children and its effect on nutritional status and physical development. *Pr Prev Med*. 2017;24(07):07.

115.	Xu MY, Cao B, Yuan BS, others. Association of anaemia with Helicobacter pylori infection: a retrospective study. *Sci Rep*. 2017;7:1-7. doi:10.1038/s41598-017-13955-3

116.	Qu SJ, Bai XS, Chen LH, others. Helicobacter pylori among people receiving health check-ups. *Chin J Geriatr*. 2017;36:214-218.

117.	Shan JH, Bai XJ, Han LL, others. Changes with aging in gastric biomarkers levels and in biochemical factors associated with Helicobacter pylori infection in asymptomatic Chinese population. *World J Gastroenterol*. 2017;23:5945. doi:10.3748/wjg.v23.i32.5945

118.	Yu . X., Yang X, Yang T, others. Decreasing prevalence of Helicobacter pylori according to birth cohorts in urban China. *Turk J Gastroenterol*. 2017;28:94-97. doi:10.5152/tjg.2017.16557

119.	Zhang XH, Chen HH, Yang G. Analysis of the Helicobacter pylori infection status among health check-up population. *Chin J Soc Med*. 2017;34(03):03.

120.	Zhao S, Xu XW, Li AH. Epidemiology of Helicobacter pylori infection in asymptomatic children in Beijing areas. *Chin J Pract Pediatr*. 2017;32(10):10.

121.	Hu A, Li L, Hu C. Zhang DM, et al. Serum concentrations of 15 elements among Helicobacter pylori-infected residents from Lujiang County with high gastric cancer risk in Eastern China. *Biol Trace Elem Res*. 2018;186(01):01. doi:10.1007/s12011-018-1283-4

122.	Gao YR, He QF, Wang LJ, Chen N, Chen Y. Research on detection value of 14C-urea breath test for Helicobacter pylori infection in health check-up population of Wuchang District. *Chin J Nosocomiol*. 2018;28(19):19.

123.	Xu MY, Ma JH, Yuan BS, Yin J, Liu L, Lu QB. Association between Helicobacter pylori infection and gallbladder diseases: A retrospective study. *J Gastroenterol Hepatol*. 2018;33(06):06. doi:10.1111/jgh.14054

124.	Meng FN, Sun L. Analysis of Helicobacter pylori infection in healthy population in Chongqing area. *J Mod Lab Med*. 2018;33(05):05.

125.	Su Z, Fei SJ, Wang K. Analysis of Helicobacter pylori infection and its related factors in physical examination people in Xuzhou. *Lab Med Clin*. 2018;15(21):21.

126.	Sun HX, Zou Z, Huang YP, et al. Epidemiological survey on Helicobacter pylori infection among Shanghai suburb residents. *Shanghai J Prev Med*. 2018;30(04):04.

127.	Wei WZ, Liu YR, Wen XH, et al. The relationship between Helicobacter pylori infection and hyperhomocysteinemia in healthy sybjects. *Chin J Health Care Med*. 2018;20(01):01.

128.	Yu . X. H., Ma J, Zhang JC, et al. Features analysis of HP infection of healthy adults. *West J Tradit Chin Med*. 2018;31(11):11.

129.	Zhou Y, Ye Z, Huang J, others. High prevalence and low spontaneous eradication rate of Helicobacter pylori infection among schoolchildren aged 7–12 years. *Acta Paediatr Stockh*. 2018;107:1624-1628. doi:10.1111/apa.14387

130.	Hou B, Zhang M, Liu M, others. Association of active Helicobacter pylori infection and anemia in elderly males. *BMC Infect Dis*. 2019;19:1-9. doi:10.1186/s12879-019-3849-y

131.	Hu L, Wan ZC, Lv YM, Yang SB. Correlation of Helicobacter pylori infection with serum bilirubin. *Chin J Gastroenterol*. 2019;24(01):01.

132.	Shu L, Zheng PF, Zhang XY, others. Dietary patterns and Helicobacter pylori infection in a group of Chinese adults ages between 45 and 59 years old: An observational study. *Medicine (Baltimore)*. 2019;98. doi:10.1097/MD.0000000000014113

133.	Zhang LL, Li NN, Zou HM, others. Current Status and Influencing Factors of Helicobacter pylori Infection in Health Examination Population and Outpatients in Baoshan District, Shanghai. *Chin J Gastroenterol*. 2019;24:326-330.

134.	Gao T, Zhao M, Zhang C, others. Association of Helicobacter pylori infection with vitamin D deficiency in infants and toddlers. *Am J Trop Med Hyg*. 2020;102:541. doi:10.4269/ajtmh.19-0523

135.	Zhu HM, Li BY, Tang Z, others. Epidemiological investigation of Helicobacter pylori infection in elderly people in Beijing. *World J Clin Cases*. 2020;8:2173. doi:10.12998/wjcc.v8.i11.2173

136.	Hu J, Wang X, Chua EG, others. Prevalence and risk factors of Helicobacter pylori infection among children in Kuichong Subdistrict of Shenzhen City, China. *PeerJ*. 2020;8:e8878. doi:10.7717/peerj.8878

137.	Zhang J, Chen Y, Chen W, others. Persistent infection of Helicobacter pylori affects weight loss in obese population compared with persistent negative: A case-control study based on healthy Chinese. *Helicobacter*. 2020;25:e12697. doi:10.1111/hel.12697

138.	Wang JW, Dong FX, Su H, others. H Pylori is related to osteoporosis but only in premenopausal female: a cross-sectional study. *BMC Musculoskelet Disord*. 2020;21:1-9. doi:10.1186/s12891-020-03586-7

139.	Liu H, Fei C, Zhang J. Associations of Serum Pepsinogens and Helicobacter Pylori Infection with High- Sensitivity C-Reactive Protein in Medical Examination Population. *Lab Med*. 2021;52:57-63. doi:10.1093/labmed/lmaa042

140.	Shen L, Bian R, Wang W, others. Association of Helicobacter pylori infection with colorectal adenoma in the Chinese urban population: A cross-sectional study. *Microb Pathog*. 2021;158:105111. doi:10.1016/j.micpath.2021.105111

141.	Wang X, Shu X, Li Q, others. Prevalence and risk factors of Helicobacter pylori infection in Wuwei, a high-risk area for gastric cancer in northwest China: An all-ages population-based cross-sectional study. *Helicobacter*. 2021;26:e12810. doi:10.1111/hel.12810

142.	Li C, Yue J, Ding Z, et al. Prevalence and predictors of Helicobacter pylori infection in asymptomatic individuals: a hospital-based cross-sectional study in Shenzhen, China. *Postgrad Med*. 2022;134(7):686-692. doi:10.1080/00325481.2022.2085950

143. Feng PL, Liu RN, Li K, Qin KX, Li JB, Xu XH. Study on the relationship between Helicobacter pylori infection and overweight/obesity, blood lipids, blood glucose, and non-alcoholic fatty liver disease in adult health examination populations. Modern Preventive Medicine. 2022;49(08):1375-1378.
144. Zhou J, Wang X, Liu K, Chen K. Association between Helicobacter pylori infection and the risk of type 2 diabetes mellitus based on a middle-aged and elderly Chinese population. Endocr J. 2022;69(7):839-846. doi:10.1507/endocrj.EJ21-0591
145. Luo P, Pu K, Yang GD. Analysis of current status and related influencing factors of Helicobacter pylori infection in the Nanchong region. Modern Digestion & Intervention. 2022;27(08):951-955.
146. Shi E, Zhang Y. Correlation analysis between Helicobacter pylori infection and 14 types of food intolerance in adult health examination populations in the Qingdao region. Modern Medicine. 2022;50(07):853-860.
147. Wang YP, Ma PP, Tian DD. Analysis of current status and influencing factors of Helicobacter pylori infection among health examination populations in the Zhengzhou region. South China Journal of Preventive Medicine. 2022;48(08):1003-1005+1009.
148. Xue JH, You AJ, Xu ZY, Wang XY, Tao Q, Yu HP. Study on the relationship between Helicobacter pylori infection and lifestyle and risk factors among occupational health examination populations in the Shanghai region. Modern Preventive Medicine. 2022;49(03):404-410.
149. Wu Y, Zeng H, Zhang M, others. Sex-Specific Risk Factors Associated with Helicobacter pylori Infection Among Individuals Undergoing Health Examinations in China. Int J Gen Med. 2022;15: 5861-5868. doi:10.2147/IJGM.S367142
150. Zhou Y, Deng Y, You Y, others. Prevalence and risk factors of Helicobacter pylori infection in Ningxia, China: comparison of two cross-sectional studies from 2017 and 2022. Am J Transl Res. 2022;14(9):9.
151. Chang J, Feng Y, Cao R, Zhu K. Study on the current status of Helicobacter pylori infection and its association with lifestyle among residents in the Xi'an region. South China Journal of Preventive Medicine. 2023;49(10):1233-1238.
152. Chen LZ, Cai YH, Zhang JJ, Chen YF, Lin ZQ, Wang CR. Correlation analysis between non-alcoholic fatty liver disease and Helicobacter pylori infection. Chinese Journal of Pathogen Biology. 2023;18(12):1434-1437+1442. doi:10.13350/j.cjpb.231213
153. Chen RX, Zhang DY, Zhang X, et al. A survey on Helicobacter pylori infection rate in Hainan Province and analysis of related risk factors. BMC Gastroenterol. 2023;23(1):338. doi:10.1186/s12876-023-02973-3
154. Liao F, Zhu Z, Zhu S, et al. Investigation of the prevalence and risk factors of Helicobacter pylori infection and the value of different gastric cancer screening methods in a low-risk region of gastric cancer in China. Ann Med. 2023;55(2):2243988. doi:10.1080/07853890.2023.2243988
155. Jiang JJ, Liu HX, Shen WJ, Pei JL, Gu FY, Gao XY. Analysis of Helicobacter pylori infection detection results and comparison of methods in adult healthy populations. Labeled Immunoassays and Clinical Medicine. 2023;30(05):782-785+816.
156. Zou JC, Wen MY, Huang Y, Chen XZ, Hu JK. Helicobacter pylori infection prevalence declined among an urban health check-up population in Chengdu, China: a longitudinal analysis of multiple cross-sectional studies. Front Public Health. 2023;11:1128765. doi:10.3389/fpubh.2023.1128765
157. Lan QZ, Hao YY, Gao H, Cheng SQ, Li BQ. A cross-sectional study on the status of Helicobacter pylori infection among health examination populations. Chinese Medicine. 2023;18(09):1328-1332.
158. Liu HQ, Yang J, Wang R, et al. A comparative study on Helicobacter pylori infection status among Tibetan and Han families in Southwest China. Chinese Journal of Gastroenterology. 2023;28(10):577-583.
159. Tong J, Chen XH, Wang YM, et al. Analysis of Helicobacter pylori infection status and related risk factors among household residents in Luzhou. Modern Digestion & Intervention. 2023;28(01):23-27.
160. Wang G, Zhang ZY, Mei YP, Wang YS. Analysis of Helicobacter pylori 13C urea breath test and immunotyping results among health examination populations in the Nanjing region. Labeled Immunoassays and Clinical Medicine. 2023;30(01):57-60+169.
161. Wang R, Liu MZ, Zhang JT, Liu N, Wang JH, Song YH. Investigation on the prevalence and influencing factors of Helicobacter pylori infection among rural populations in Gongli Town, Fuping County, Shaanxi Province. Shaanxi Medical Journal. 2023;52(09):1264-1267+1271.
162. Wang XC, Cao GS, Zhu ZD, Zheng XM. Analysis of Helicobacter pylori infection status and influencing factors among health examination populations in Danzhou City, 2020–2021. Modern Preventive Medicine. 2023;50(12):2259-2264. doi:10.20043/j.cnki.MPM.202210199
163. Xiao Y, Li JX, Liao J, et al. Study on the association between Helicobacter pylori infection and blood lipid levels. Modern Preventive Medicine. 2023;50(10):1903-1907. doi:10.20043/j.cnki.MPM.202209577
164. Liu Y, Shuai P, Chen W, Liu Y, Li D. Association between Helicobacter pylori infection and metabolic syndrome and its components. Front Endocrinol. 2023;14:1188487. doi:10.3389/fendo.2023.1188487
165. Zhao YM, Wu JJ, He JN, et al. Analysis of current status and related factors of Helicobacter pylori infection among health examination populations. Journal of Medical Pest Control. 2023;39(05):439-443+447.
166. Zhou XZ, Lyu NH, Zhu HY, et al. Large-scale, national, family-based epidemiological study on Helicobacter pylori infection in China: the time to change practice for related disease prevention. Gut. 2023;72(5):855-869. doi:10.1136/gutjnl-2022-328965
167. He L. Study on factors associated with Helicobacter pylori infection and immunological typing among community health examination populations. Public Health and Preventive Medicine. 2024;35(02):83-86.
168. Wang L, Li Z, Tay CY, Marshall BJ, Gu B. Multicentre, cross-sectional surveillance of Helicobacter pylori prevalence and antibiotic resistance to clarithromycin and levofloxacin in urban China using the string test coupled with quantitative PCR. Lancet Microbe. 2024;5(6):e512-e513. doi:10.1016/S2666-5247(24)00027-2
169. Tang J, Ning LY, Leng BS. Analysis of current Hp infection status and related influencing factors among 610 young and middle-aged health examination individuals. Practical Preventive Medicine. 2024;31(08):992-995.
170. Wang XX, Zhang T, Qin W, Luo H, Luo XM. Investigation of Helicobacter pylori infection status and its influencing factors among rural populations in Kunshan City. Shanghai Medical Journal. 2024;47(05):289-296. doi:10.19842/j.cnki.issn.0253-9934.2024.05.007
171. Wang ZF, Cao W, Yang XM, Hu CQ. Analysis of current status and related factors of Helicobacter pylori infection among health examination populations in the Kashgar region, Xinjiang. Journal of Clinical Internal Medicine. 2024;41(08):550-553.
172. Wu HM, Zhao XL. Risk factors for Helicobacter pylori infection among health examination populations and their correlation with serum PGI, PGII, and G-17. China Medical Herald. 2024;21(08):78-81. doi:10.20047/j.issn1673-7210.2024.08.18
173. Yan X, Zeng H, Li H, et al. The current infection with Helicobacter pylori and association with upper gastrointestinal lesions and risk of upper gastrointestinal cancer: Insights from multicenter population-based cohort study. Int J Cancer. 2024;155(7):1203-1211. doi:10.1002/ijc.34998
174. Jiang Q, Liu WD, Hui WJ, et al. Prevalence and risk factors of Helicobacter pylori infection in Xinjiang Uygur Autonomous Region: A cross-sectional study of all age groups. World J Gastroenterol. 2025;31(21). doi:10.3748/wjg.v31.i21.106033
175. Li Q, Shen J, Shi WQ, Lin N, Hou SQ. Analysis of current status and risk factors of Helicobacter pylori infection among health examination populations in the Chuzhou region, Anhui Province. Chinese Journal of General Practice. 2025;23(05):765-767+870. doi:10.16766/j.cnki.issn.1674-4152.003996
176. Liu JZ, Gao JC, Zhang WH, Xu YL, Liu JL. Analysis of Helicobacter pylori infection status and influencing factors among adolescents in the Cangzhou region. South China Journal of Preventive Medicine. 2025;51(01):114-117.
177. Sun B, Guo YN, Dong MM, Fang LJ, Li XJ. Analysis of current status and related influencing factors of Helicobacter pylori infection among young and middle-aged health examination populations. Chinese Journal of Pathogen Biology. 2025;20(04):481-485. doi:10.13350/j.cjpb.250414
178. Qin FD, Wu FL, Wei ZM, Ban LS, Huang R, Guo XW. Current status of Helicobacter pylori infection among middle school students in the Huanjiang Maonan ethnic area of Guangxi. Journal of Tropical Medicine. 2025;25(04):545-549.
179. Wu YT, Deng YH, Tang YY, Liu J, Ma LK, Hu SJ. Analysis of current status and related factors of Helicobacter pylori infection in the Yinchuan region of Ningxia in 2021. Translational Medicine Journal. 2025;14(02):30-34+62.
180. Yan L, He Q, Peng X, et al. Prevalence of Helicobacter pylori infection in the general population in Wuzhou, China: a cross-sectional study. Infect Agent Cancer. 2025;20(1):1. doi:10.1186/s13027-024-00632-0
181. Zhang J, Yuan W, Xing Y, Ma X, Xuan F. Helicobacter pylori infection, clarithromycin-resistant genes and CYP2C19 gene polymorphisms in asymptomatic school-age children in Baigou New Town, Baoding City, Hebei Province. Pak J Med Sci. 2025;41(2):484. doi:10.12669/pjms.41.2.9838
