# Supplementary material for: Trends in Helicobacter pylori prevalence, force of infection, and attributable disease burden in China: a systematic review and Bayesian modelling study
Source: Lancet Reg Health West Pac. 2026 Sep 10;74:101979. doi: 10.1016/j.lanwpc.2026.101979 (PMC13584135; doi:10.1016/j.lanwpc.2026.101979)
Supplement: Supplementary file 2 [file mmc2.docx]

**摘要**

**背景**
幽门螺杆菌感染是胃癌和消化性溃疡病的主要感染性病因。中国是全球感染人数最多的国家之一，但其长期年龄别感染动态变化及归因疾病负担尚未得到充分阐明。

**方法**
我们系统检索并综述了报告中国幽门螺杆菌感染率的研究，提取年龄别感染率数据。通过构建贝叶斯模型，整合现症感染率、血清阳性率及曾感染率数据，在考虑研究异质性与诊断误分类的基础上，估计1990-2023年的年龄别感染力与感染率。进而将模型估计的感染率与已发表的相对风险及全国病例数相结合，估算幽门螺杆菌感染的人群归因分值，以及可归因于该感染的胃癌和消化性溃疡病病例数。

**结果**
贝叶斯模型共纳入181项研究，覆盖所有年龄组。1980年后出生队列的儿童期感染力明显低于更早出生队列，提示儿童期感染风险已有所减少。然而，人群感染率仅从1990年的43.1%（95%可信区间：39.1%–47.4%）缓慢下降至2023年的36.3%（33.2%–39.6%）。2023年，非贲门胃癌和贲门胃癌的人群归因分值分别为70.1%（95%不确定性区间：51.4%–82.4%）和49.4%（20.5%–70.7%）。约有34万–37万例胃癌及16万–21万例消化性溃疡病可归因于幽门螺杆菌感染。

**解释**
中国的幽门螺杆菌感染率及其归因疾病负担仍然较高。其下降速度较既往研究提示的更为缓慢，我们的研究结果支持采取更为积极的幽门螺杆菌防控策略。
